# Base‐Mediated Scalable Synthesis of Polybenzothiazoles: Fused‐Heterocycle‐Engineered Recovery of Precious Metals

**DOI:** 10.1002/advs.202506580

**Published:** 2025-08-13

**Authors:** Hongjie Zhou, Xiaoqiang An, Tianshu Zhang, Mingran Li, Lingru Kong, Huachun Lan, Huijuan Liu, Jiuhui Qu

**Affiliations:** ^1^ Center for Water and Ecology State Key Laboratory of Iron and Steel Industry Environmental Protection School of Environment Tsinghua University Beijing China

**Keywords:** heterocycle‐mediated coordination, multicomponent polymerization, polybenzothiazoles, precious metal recovery, sulfur‐based synthesis

## Abstract

Sulfur‐containing fused heterocyclic polybenzothiazoles are promising materials with advanced functionalities, yet their synthesis has long been constrained by substrate limitations and scalability challenges. Here, a base‐mediated multicomponent polymerization strategy using readily available elemental sulfur, aromatic diamines, and aromatic dialdehydes is developed to synthesize unprecedented polybenzothiazoles with scalability. By efficient alkaline activation of substrates through nucleophilic sulfurization‐cyclization cascades, this method enables economically viable kilogram‐scale production in a one‐pot process with high yields (73–98%) and monomer universality, including previously incompatible electron‐deficient aromatic amines. The resulting polybenzothiazoles unlock their long‐overlooked potential in precious metal recovery, demonstrating selective, rapid, and efficient extraction (>99%) of gold (Au), palladium (Pd), and platinum (Pt) from ultra‐trace concentrations (1 ppb) to complex matrices including surface water, e‐waste, and spent catalyst leachates. Mechanistic studies reveal that the synergistic nitrogen (N)/sulfur (S) participation and π‐conjugation in their fused heterocycles govern metal coordination selectivity and redox stability. This work establishes a practical yet versatile platform to advance polybenzothiazoles from synthesis to resource utilization, highlighting their transformative role in addressing critical challenges through adaptive material design and precious metal recovery.

## Graphical abstract

Polybenzothiazoles with scalability and tunability are synthesized from base‐mediated multicomponent polymerization of sulfur, aromatic diamines, and dialdehydes, addressing previous synthetic limitations. The fused heterocyclic S‐ and N‐containing polymers reveal their capability to recover precious metals Au, Pd, and Pt from complex waste streams with high efficiency, selectivity, robustness, and adaptability.

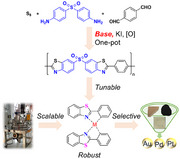

## 1 Introduction

Sulfur‐containing fused heterocyclic polymers represent an important class of functional polymers combining robust polymer frameworks with dynamic electronic properties.^[^ ^1^, ^2^ ^]^ The interplay between conjugated fused rings and sulfur‐rich backbones endows these polymers with attractive properties, such as high thermal and chemical stability, exceptional optoelectronic properties, and unique metal‐coordination activities.^[^ ^3^, ^4^, ^5^, ^6^ ^]^ As a prototype, polybenzothiazoles have garnered broad interest across fields including organic optoelectronics, catalytic engineering, protective coatings, and energy storage systems, positioning them as versatile platforms with multifunctionality.^[^ ^7^, ^8^, ^9^, ^10^ ^]^ Recently, heterocycle‐driven precious metal recovery highlights a revolutionary potential in sustainable resource recycling, exemplified by polycarbenes, polypyrrolidines, and porphyrin‐phenazine polymers.^[^ ^11^, ^12^, ^13^ ^]^ Whereas prevalent coordinating motifs (e.g., amine/thiol functionalities) are restricted by monodentate flexibility and redox vulnerability, fused heterocycles offer programmable multidentate architectures and rigid scaffolds that enable stabilization of high‐valent metal species with redox resilience.^[^ ^14^, ^15^ ^]^ However, the practical deployment of these polymers faces a critical dilemma: reconciling high‐performance heterocyclic materials with economic scalability remains challenging due to synthetic complexity and constrained monomer accessibility.

**Figure 1:**
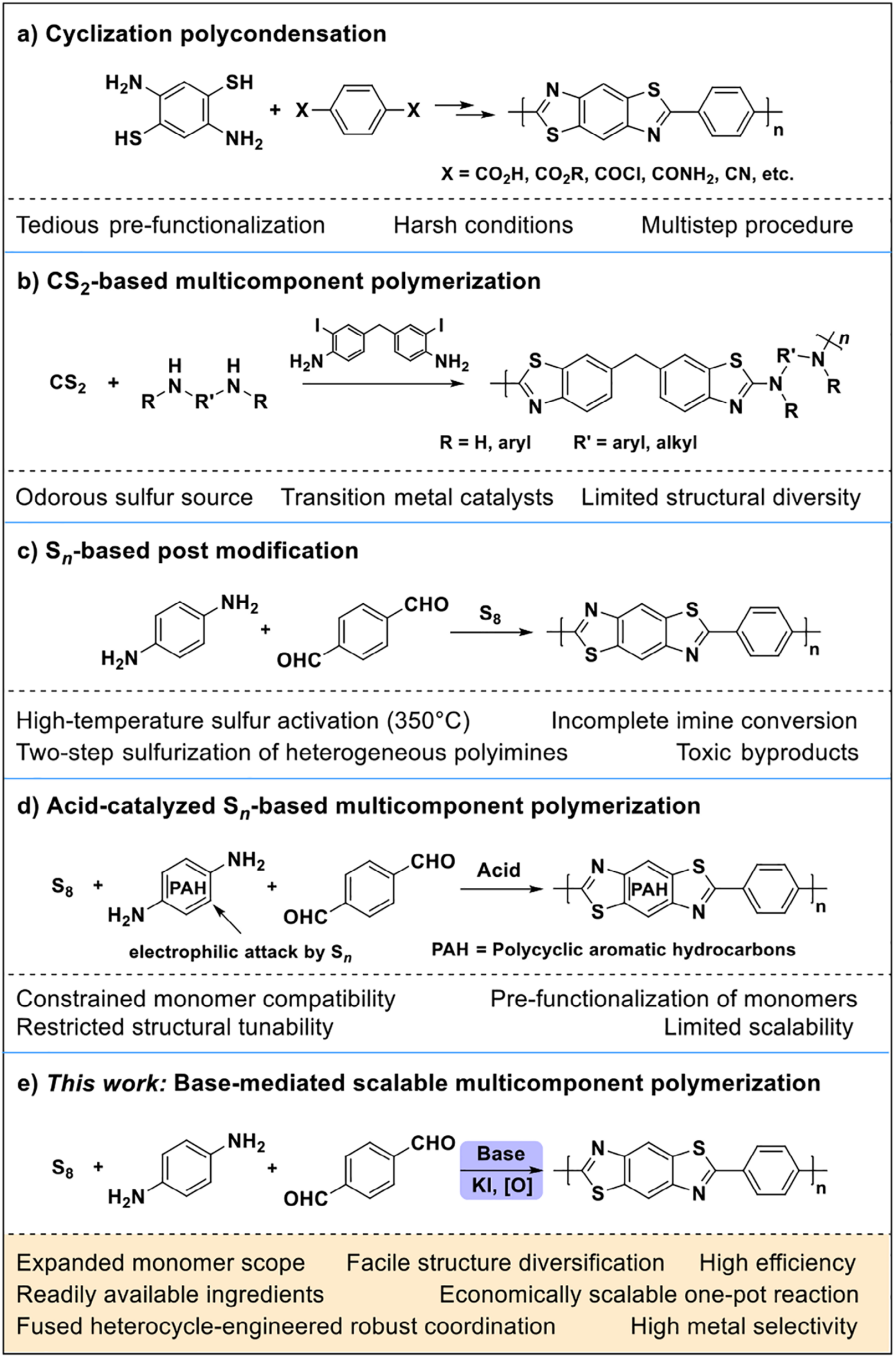
Comparison of a–d) previous construction methods toward polybenzothiazoles with e) the new method developed in this work.

The synthetic challenges of polybenzothiazoles stem from inherent limitations in existing methodologies. Prevailing C–C coupling strategies, including cross‐coupling polymerization and direct/oxidative C–H arylation polymerization,^[^ ^16^, ^17^, ^18^ ^]^ rely on the use of precious/transition metal catalysts, and can only introduce pre‐constructed heterocycle building blocks into conjugated backbones. The structural diversity is further restricted by tedious pre‐functionalization procedures of monomers. Cyclization polycondensation of bis(*o*‐aminobenzenethiol) monomers enables in situ benzothiazole formation in the polymer backbone (**Figure** **1**a), however, the cumbersome preparation of such monomers hinders the tunability of polymer structure.^[^ ^19^, ^20^ ^]^ Moreover, this method typically involves multi‐step procedures with high reaction temperatures (350–400 °C). Recent advances in multicomponent polymerizations (MCPs) using carbon disulfide (CS_2_), diamines, and bis(2‐iodoaniline)s enable polybenzothiazole synthesis,^[^ ^21^ ^]^ but they still require transition metal catalysts, additional steps for iodoaniline diversification, and malodorous sulfur sources (Figure 1b). MCPs utilizing elemental sulfur (S_8_) offer promising alternatives for synthesizing sulfur‐containing polymers.^[^ ^22^ ^]^ For instance, the S_8_‐mediated oxidative cyclization of imines has succeeded in constructing benzothiazole rings,^[^ ^23^, ^24^ ^]^ yet their application has rarely been extended to polybenzothiazoles, with reported examples confined to thiazole‐linked covalent organic frameworks (COFs). The post‐modification of imine‐linked frameworks with S_8_ demands high‐temperature activation (350 °C) for heterogeneous sulfurization, while still suffering from incomplete imine conversion and toxic byproducts (Figure 1c).^[^ ^25^ ^]^ Acid‐catalyzed MCP approaches following electrophilic sulfurization pathways are constrained to electron‐rich polycyclic aromatic diamine monomers,^[^ ^26^ ^]^ with an inherent incompatibility with readily available and versatile aniline substrates, elevating costs from pre‐functionalization procedures while posing challenges to structural tunability and scalability (Figure 1d). Aniline substrates, particularly electron‐deficient ones, have shown widespread inefficacy in benzothiazole construction under existing neutral/acidic conditions.^[^ ^27^, ^28^ ^]^ These cumulative limitations have stalled progress: despite the theoretical promise of polybenzothiazoles for precious metal recovery with sulfur‐rich fused heterocycles, their exploration in this context remains absent. A facile, straightforward, and efficient polymerization method is urgently needed to produce multifunctional polybenzothiazoles with economic scalability and readily tunable structures toward broader industrial application scenarios.

Herein, we present a paradigm‐shifting base‐mediated MCP strategy of sulfur/diamine/dialdehyde to efficiently synthesize unprecedented polybenzothiazoles (Figure 1e). This approach leverages the alkaline activation of S_8_ and low‐reactivity aromatic diamines, coupled with iodide/oxidant‐assisted sulfurization/cyclization in a one‐pot, transition‐metal‐free reaction. Compared to previous methods, this method expands the substrate scope to include readily accessible diamines and dialdehydes to synthesize versatile polybenzothiazoles. Facile scalability with high yields and cost‐effective kilogram‐scale production capacity was achieved. Building on this synthetic strategy, we advanced the application of polybenzothiazoles to efficient and selective precious metal (Au, Pd, and Pt) recovery through robust heterocycle‐metal coordination. Structure‐property correlations reveal facile tunability through rational motif engineering to enhance functional practicality.

## 2 Results and Discussion

**Figure 2:**
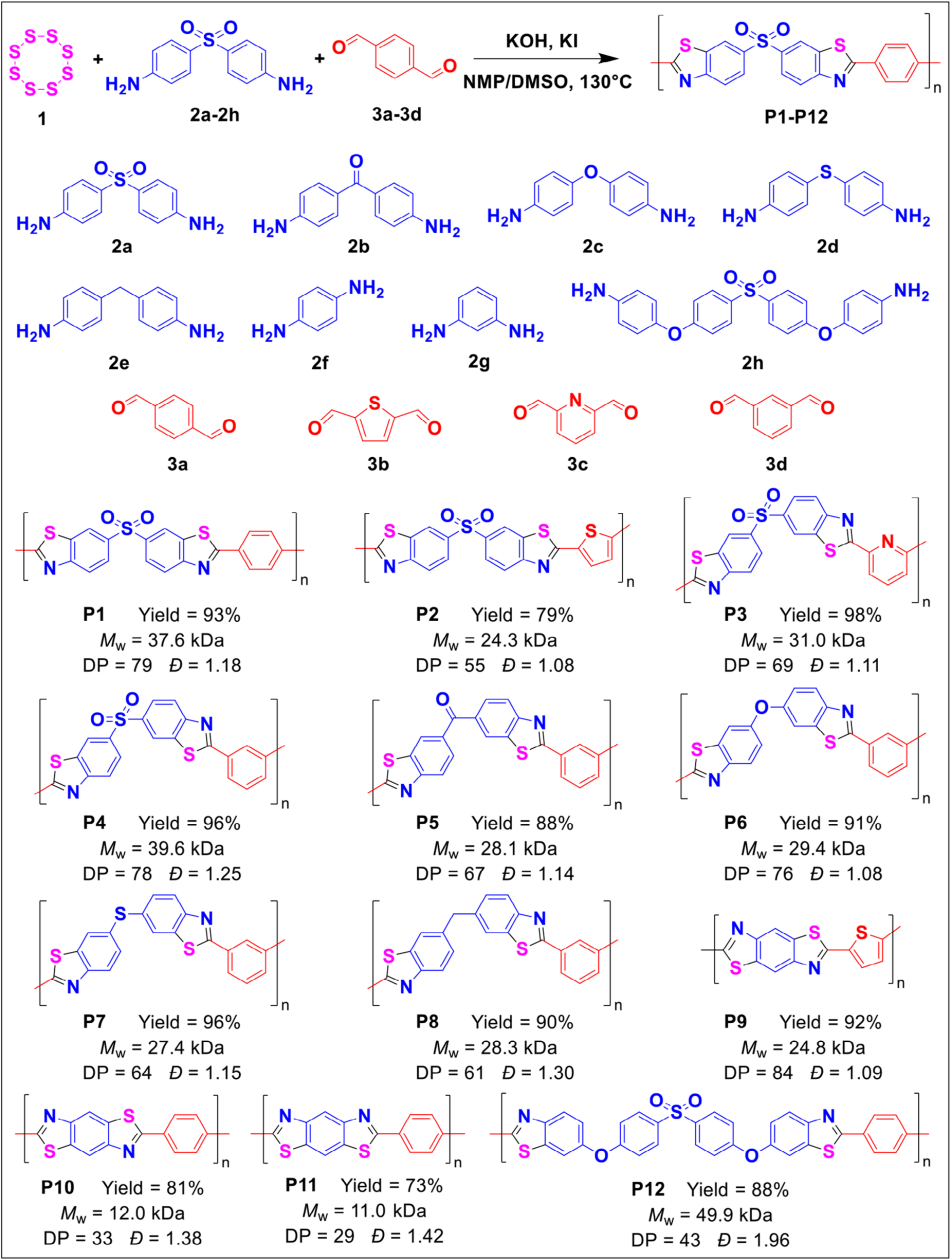
Synthesis of polybenzothiazoles **P1**–**P12** via base‐mediated multicomponent polymerization of elemental sulfur (**1**), aromatic diamines (**2a**–**2h**), and aromatic dialdehydes (**3a**–**3d**). Carried out at 130 °C in NMP/DMSO (v/v = 3:1) for 24 h under air with a general monomer and additive loading ratio of 1/8[S_8_]:[**2a**–**2h**]:[**3a**–**3d**]:[KOH]:[KI] at 4.0:2.0:1.0:2.0:0.2. Isolated yields were calculated based on the dialdehyde monomers. *M*_w_ and *M*_n_ were determined by gel permeation chromatography (GPC) in dimethylformamide (DMF) with soluble fractions of the polymer products. Degree of polymerization (DP) is determined by dividing *M*_n_ of the polymer by the molecular weight of its repeating unit. Polymer dispersity (*Đ*) = *M*_w_/*M*_n_.

All monomers and additives used for polybenzothiazole synthesis were restricted to readily accessible commercial sources to facilitate cost‐effectiveness and scalability. To validate the viability of our strategy, we selected sublimed sulfur (**1**), electron‐deficient 4,4′‐diaminodiphenyl sulfone (**2a**), and terephthalaldehyde (**3a**) as model reactants to investigate the reaction conditions (**Figure** **2**). Initial attempts without base under 120 °C after 12 hours in dimethyl sulfoxide (DMSO) yielded only 16% polymer, accompanied by a substantial presence of intermediate non‐sulfurized polyimines and uncyclized polythioamides (NH─C═S). The introduction of various organic and inorganic bases, including Na_2_CO_3_, KOH, K_2_CO_3_, Cs_2_CO_3_, KF, CsF, *t*‐BuOK, triethylamine (Et_3_N), 1,8‐diazabicyclo[5.4.0]undec‐7‐ene (DBU) and 1,4‐diazabicyclo[2.2.2]octane (DABCO), significantly improved the polymer yields (Table S1, Supporting Information). Weak bases, however, could result in incomplete conversion, whereas KOH yielded the best results eliminating the intermediates to deliver polybenzothiazole in 57% yield. A proper concentration of KOH (1 m) was also found important: lower concentrations still hindered effective reaction progression, while excess addition reduced polymer molecular weight (*M*_w_) and yield (Table S2, Supporting Information), likely due to the Cannizzaro side reactions of aldehydes.^[^ ^29^ ^]^ Through screening a series of polar solvents (Table S3, Supporting Information), a 25% DMSO (v/v) in N‐methylpyrrolidone (NMP) was found optimal, leveraging DMSO's dual role as a high‐polarity solvent and an in‐situ oxidant for oxidative aromatization.^[^ ^26^ ^]^ Further optimization of monomer loading ratios revealed that excess amine and sulfur ([S_8_]:[**2a**]:[**3a**] = 4.0:2.0:1.0) enhanced polymerization efficiency (Table S4, Supporting Information), which aligns with the reactivity enhancement of these two monomers under alkaline conditions.^[^ ^30^ ^]^ Adjusting monomer concentration ([**3a**] = 0.5 m, Table S5, Supporting Information) and maintaining the reaction at 130 °C for 24 h until yield and *M*_w_ plateaued (Table S6, Supporting Information) eventually culminated in the isolation of **P1** as a crimson‐red solid with a yield of 93% (*M*_w_ of 37.6 kDa, a degree of polymerization (DP) of 79, and a dispersity (*Đ*) of 1.18).

A series of aromatic diamines (**2a**–**2h**) and aromatic dialdehydes (**3a**–**3d**) were successfully polymerized to afford polybenzothiazoles **P1**–**P12** in 73–98% yields (Table S7 and Figure S1, Supporting Information). Notably, combination involving previously challenging monomers, such as electron‐poor dianilines (**2a**) paired with electron‐rich dialdehyde (**3b**), achieved polymer yields of 79%. Furthermore, reactive monomers prone to rapid gelation (due to low‐solubility Schiff‐base formation, as exemplified in Figure S2, Supporting Information) managed to sustain near‐homogeneous polymerization by proper elevation of base concentrations. Owing to the enhanced solubility of nonlinear intermediates,^[^ ^31^ ^]^ isophthalaldehyde (**3d**) also afforded polymers with higher *M*_w_s and yields compared to terephthalaldehyde (**3a**). Moreover, rigid‐rod polymers (e.g., **P10**, **P11**) attained favorable yields despite their insolubility in organic solvents.^[^ ^32^ ^]^ These results underscore the broad synthesis diversity and efficiency of this MCP strategy, extending to previously incompatible substrates.

**Figure 3:**
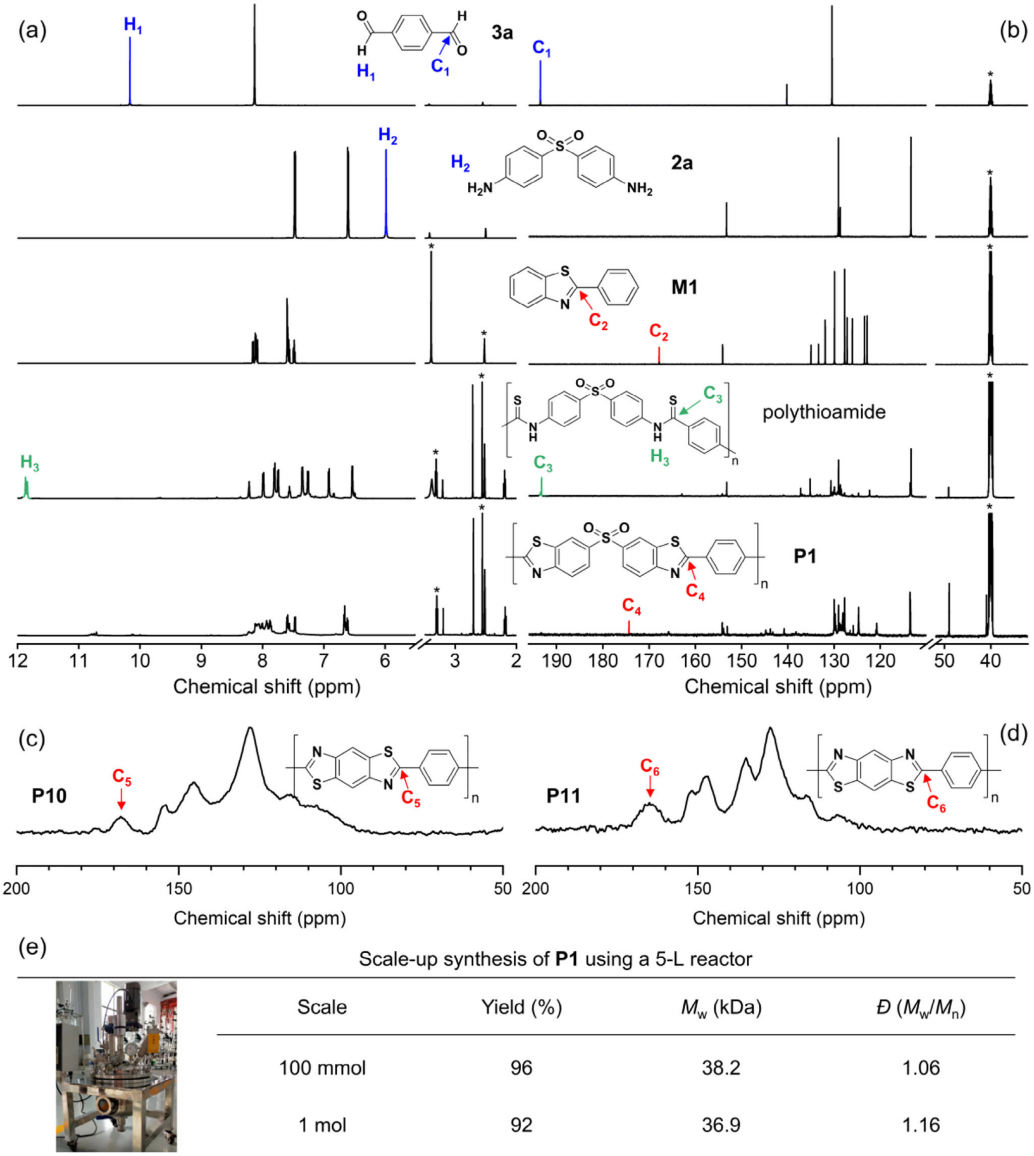
a) ^1^H NMR spectra and b) ^13^C NMR spectra of **3a**, **2a**, **M1**, intermediate polythioamide, and **P1** in DMSO‐*d*_6_. The solvent peaks were marked with asterisks. c,d) ^13^C solid‐state NMR spectra of c) **P10** and d) **P11**. e) Scale‐up synthesis of **P1** using a 5‐L reactor with 100 mmol and 1 mol of **3a**.

The polymer structures were characterized via ^1^H/^13^C nuclear magnetic resonance (NMR), infrared spectroscopy (IR), and X‐ray photoelectron spectroscopy (XPS), with comparisons to a synthesized model compound **M1** (2‐phenylbenzothiazole) validated by NMR, IR, XPS, and matrix‐assisted laser desorption/ionization time of flight mass spectrometry high‐resolution mass spectra (MALDI‐TOF MS, Figure S3, Supporting Information). Exemplarily, the ^1^H NMR spectrum of **P1** shows disappearance of the ─CHO protons at δ 10.17 from **3a** and the ─NH_2_ protons at δ 5.99 from **2a** (**Figure** **3**a). The ^13^C NMR spectrum of **P1** exhibited a distinct benzothiazole carbon resonance at δ 174.31 (Figure 3b), absent in precursors.^[^ ^21^ ^]^ IR analysis further confirmed benzothiazole formation, with **P1** displaying a newly observed characteristic C–S absorption at 619 cm^−1^, closely aligned with **M1** (623 cm^−1^, Figure S4a, Supporting Information). For polymers **P2**–**P12**, ^13^C NMR spectra (Figure S5, Supporting Information) consistently revealed benzothiazole carbons at δ 165.36–174.32 matching **M1** (δ 167.80), corroborated by ^13^C solid‐state NMR of insoluble **P10**/**P11** (Figure 3c,d), and IR peaks near 620 cm^−1^ (Figure S4b–d, Supporting Information). Notably, no residual ^1^H/^13^C NMR signals corresponding to thioamides (δ 11–13/190–210) were detected,^[^ ^31^ ^]^ confirming complete sulfur conversion to benzothiazoles. XPS analysis reinforced the structural identification (Figure S6, Supporting Information): S 2p binding energies for representative polybenzothiazoles (S 2p_1/2_ at 163.82–164.14 eV) matched **M1** (164.11 eV) and differed from polythioamides (163.07 eV). Besides, the sulfone group (─SO_2─_) of **P1** was also verified by a distinct S 2p signal at 167.91/169.11 eV.

With experimental observations and literature supports, a suggested synthetic mechanism is presented in Scheme S1 (Supporting Information). The base has three critical functions: i) S_8_ ring‐opening to polysulfide anions (**A**), ii) activation of aromatic amines for imine (**B**) formation with aldehydes through enhanced nucleophilicity, and iii) enabling **A** to execute a Willgerodt‐Kindler‐type nucleophilic attack on the imine carbon of **B**.^[^ ^33^, ^34^, ^35^ ^]^ This strategic attack site disrupts the planar conjugation of **B**, generating sulfurized intermediate (**C)** with improved solubility–a non‐negligible distinction from electrophilic sulfurization that preserve conjugation through aniline *ortho* C–H substitution (Scheme S2, Supporting Information). KI facilitates the sulfur transfer to benzothioamides (**D**) and cyclization to benzothiazolines (**E**),^[^ ^36^, ^37^ ^]^ while DMSO aids the final aromatization to benzothiazoles as an in‐situ oxidant in a one pot reaction. This strategy addresses two key limitations in electrophilic approaches: it eliminates the substrate constraints of S*_n_*‐mediated electrophilic systems (particularly for electron‐deficient dianilines) and avoids intermediate solubility loss during chain elongation.

To prove the scalability of this approach, synthesis of **P1** using MCP of **1**, **2a**, and **3a** at 100‐fold and 1000‐fold scales in a custom 5‐L reactor was conducted, achieving 96% yield (*M*_w_ = 38.2 kDa) and 92% yield (374.7 g, *M*_w_ = 36.9 kDa), respectively (Figure 3e). An economic assessment based on the 1000‐fold scale production indicated a cost of approximately $0.12 per gram of **P1** (Table S8, Supporting Information), underscoring the industrial viability of this method.

The thermal stability and morphological stability of these polybenzothiazoles were then evaluated via thermogravimetric analysis (TGA) and differential scanning calorimetry (DSC). TGA revealed good thermal stability of these polymers, with decomposition temperatures at 5 wt.% weight loss (*T*_d_) ranging from 221 to 376 °C, and substantial char yields of 32–67% at 800 °C (Figure S7a, Supporting Information), possibly attributed to the presence of rich fused heterocyclic moieties. These polymers exhibited tunable glass transition temperatures (*T*_g_) of 90–136 °C (Figure S7b, Supporting Information), likely due to the non‐conjugated segments introduced into the polymer backbone. This structural feature also contributes to good solubility of these non‐conjugated polymers in common organic solvents (Figure S8, Supporting Information), facilitating their processability.

These water‐insoluble polybenzothiazoles (Figure S9, Supporting Information) revealed their applicability for precious metal recovery from aqueous media. To unravel structure‐function relationships, three representative polybenzothiazoles spanning distinct structural motifs—**P1** (sulfone‐modified), **P8** (methylene‐modified), and **P10** (fully conjugated)—were introduced into aqueous solutions containing Au^3+^ (AuCl_3_), Pd^2+^ (PdCl_2_), and Pt^4+^ (PtCl_4_). Comparative kinetic studies revealed critical architectural influences: compared to fully conjugated **P10**, **P1** and **P8** incorporating non‐conjugated segments exhibited accelerated kinetics (Figure S10 and Table S9, Supporting Information), possibly due to alleviated intermolecular aromatic stacking and improved metal‐ion accessibility. Notably, sulfone‐containing **P1** exhibited the fastest kinetics, attributable to its enhanced hydrophilicity, supported by water contact angle measurements (37° for **P1** vs 50°–80° for **P6–P8** with counterparts, Figure S11, Supporting Information). This shows how our synthetic strategy can reconcile an inherent conflict between synthetic feasibility and functionality: while strong electron‐withdrawing groups (e.g., sulfone) in dianilines are typically avoided in conventional synthesis methods due to their incompatibility with electrophilic sulfurization pathways, their strategic integration here aligns with an application need for polarity‐driven hydrogen bonding and enhanced hydrophilicity for aqueous metal recovery.^[^ ^38^, ^39^ ^]^ The optimal polymer **P1** achieved Langmuir maximum capacities of 634 mg g^−1^ for Au, 234 mg g^−1^ for Pd, and 179 mg g^−1^ for Pt (Figure S12 and Table S10, Supporting Information). These values notably exceed those of most reported precious metal recovery materials (Table S11, Supporting Information), particularly for non‐reductive Pd and Pt systems, suggesting the critical role of benzothiazole‐metal binding interactions.

**Figure 4:**
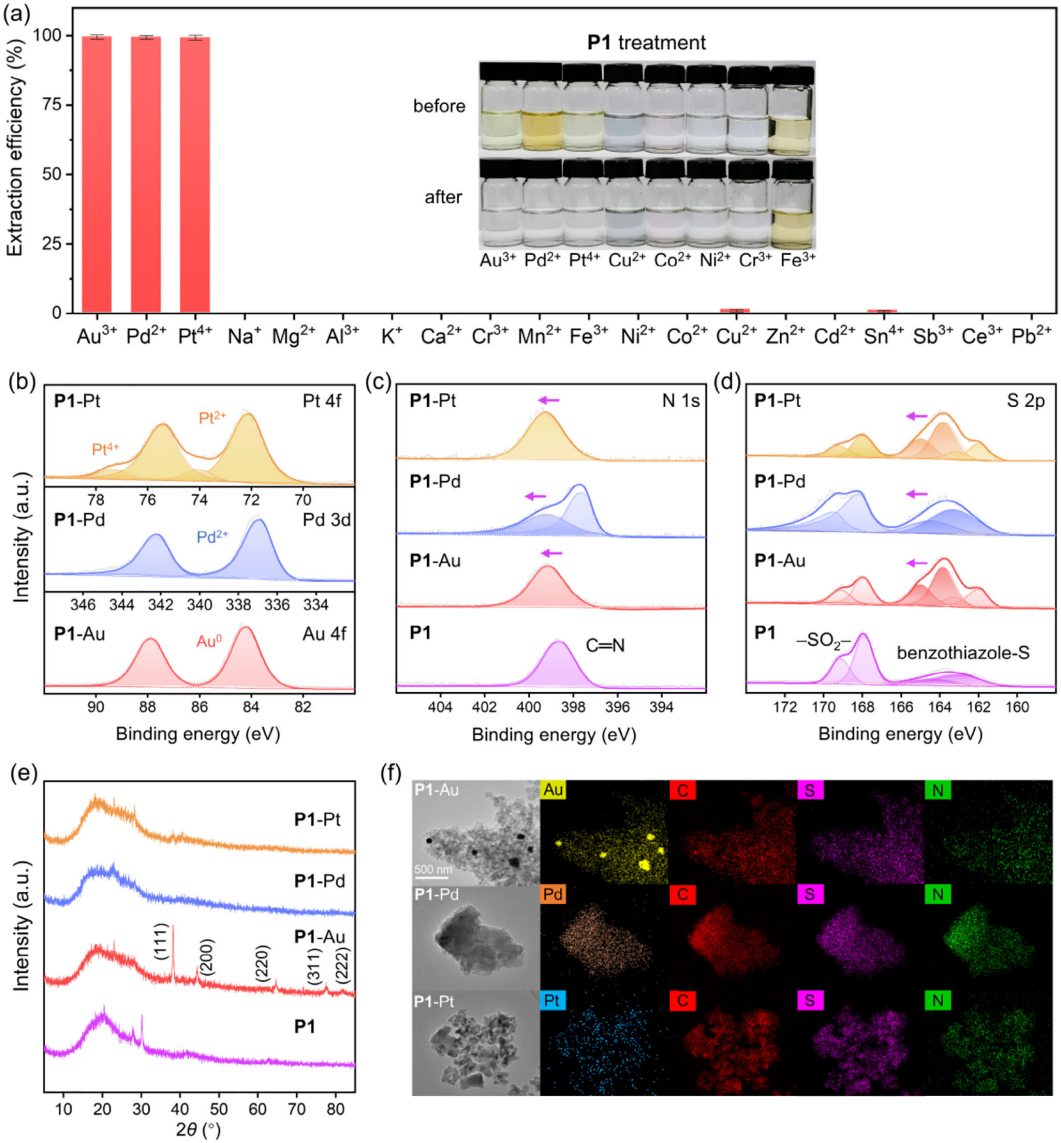
Precious metal extraction by **P1**. a) Selective extraction of precious metals by **P1** (1 g L^−1^) in aqueous solutions with 20 mixed metal ions (10 ppm). Inset: Eight colored metal ion solutions (100 ppm) before and after the treatment with **P1**. b–d) XPS spectra of post‐extraction **P1**‐metal complexes: b) Au 4f, Pd 3d, Pt 4f, c) N 1s, and d) S 2p spectra. e) Powder XRD spectra. f) STEM mapping.

The metal selectivity of **P1** was then examined using an acidic solution containing 20 metal ions (10 ppm each, **Figure** **4**a). Intriguingly, the polymer exhibited an ultrahigh selectivity for Au^3+^, Pd^2+^, and Pt^4+^ with >99% efficiency, while uptake of other metals remained below 1.0%. Additional tests in colored single‐ion solutions (100 ppm) confirmed >99% extraction efficiency of precious metals, with negligible color changes observed for other metal solutions (inset of Figure 4a; Figure S13, Supporting Information). Even at trace metal concentrations (0.1 ppm), **P1** retained 99% recovery efficiency for precious metals and <0.5% for 17 coexisting metals (Figure S14, Supporting Information). Furthermore, in a challenging simulated environment using Yangtze River water spiked with ultra‐trace precious metals (1 ppb) and 10 000‐fold excess (10 ppm) of Cu^2+^, Fe^3+^, and Ni^2+^, **P1** maintained high extraction efficiency (>97%) and selectivity for precious metals (Figure S15, Supporting Information). These results collectively demonstrate the targeted precious metal extraction capability of **P1** across a wide metal concentration range (1 ppb to 100 ppm) in complex water matrices.

The influence of pH on extraction performance was evaluated. **P1** maintained high extraction efficiencies (>99% for Au^3+^, >98% for Pd^2+^, and >90% for Pt^4+^) across a broad pH range of 0.1–9.0 (Figure S16, Supporting Information). The observed reduction in efficiency under alkaline conditions correlates with increasingly negative zeta potentials (Figure S17, Supporting Information), suggesting electrostatic repulsion between the charged polymer surface and metal chloroanions, which is not uncommon in other reported materials.^[^ ^40^, ^41^ ^]^ Zeta potential analysis revealed an isoelectric point at pH 6–7, aligning with the basic character of benzothiazole motifs. Under acidic conditions, progressively positive zeta potentials (+41.4 ± 0.5 mV at pH 2) suggest protonation at benzothiazole N‐sites,^[^ ^42^ ^]^ which enhances extraction efficiency in typical acidic industrial waste streams through favorable electrostatic interactions.

The solubility of these polybenzothiazoles in organic solvents enables versatile application in liquid‐phase extraction and membrane‐based separation.^[^ ^43^ ^]^ A prototype application of **P1** was shown by fabricating a porous membrane via phase inversion (from a 4 wt.% **P1** casting solution with polyvinylpyrrolidone and polyethersulfone in dimethylacetamide (DMAc)). Surface/cross‐sectional SEM revealed a macropore‐dominated architecture (Figure S18a,b, Supporting Information), while mercury intrusion porosimetry quantified 47.63% interstitial porosity and revealed hierarchical pore organization (Figure S18c,d, Supporting Information). This structure combines macroporous networks for enhanced permeability with micro‐/mesopores that enable surface‐area‐driven extraction. Single‐pass dead‐end filtration studies under varying feed concentrations, flux levels, and durations (Figure S19, Supporting Information) demonstrated high extraction efficiency and selectivity for trace metal recovery (>98% for Au^3+^, Pd^2+^, Pt^4+^ at 100 ppb) with a flux of 240 L m^−2^ h^−1^ bar^−1^, enabling rapid separation of precious metals from dilute streams. However, efficiency declined at elevated feed concentrations (1–10 mg L^−1^) or higher fluxes (e.g., operating under 1 bar transmembrane pressure) due to insufficient hydraulic residence time, thus implying the inherent performance‐permeability trade‐off and indicating the need for multi‐stage operation to enhance applicability.^[^ ^44^ ^]^

The extraction mechanism of **P1** was then systematically deciphered through complementary spectroscopic and computational approaches. Post‐extraction XPS analysis of **P1**‐metal complexes revealed characteristic Au 4f, Pd 3d, and Pt 4f signals (Figure S20, Supporting Information). Deconvoluted spectra (Figure 4b–d) identified Au^0^ (84.17/87.85 eV), Pd^2+^ (336.89/342.18 eV), and mixed Pt^4+^/Pt^2+^ states (74.02/77.34 eV and 72.07/75.37 eV), indicating combined metal ion coordination and reduction behaviors. This observation was corroborated by X‐ray diffraction (XRD) and scanning transmission electron microscopy (STEM) analyses (Figure 4e,f): crystalline Au(0) nanoparticles contrasted with amorphous Pd/Pt species, confirming ionic adsorption without metallic crystallization for latter two systems. Critical evidence for benzothiazole‐mediated coordination emerged from ligand‐centric XPS spectral shifts. The benzothiazole N 1s component increased from 398.62 eV (free **P1**) to 399.12–399.25 eV upon metal binding, while S 2p_1/2_ elevation from 164.14 eV to 164.41–164.92 eV established both heteroatoms as coordination participants.^[^ ^45^ ^]^

**Figure 5:**
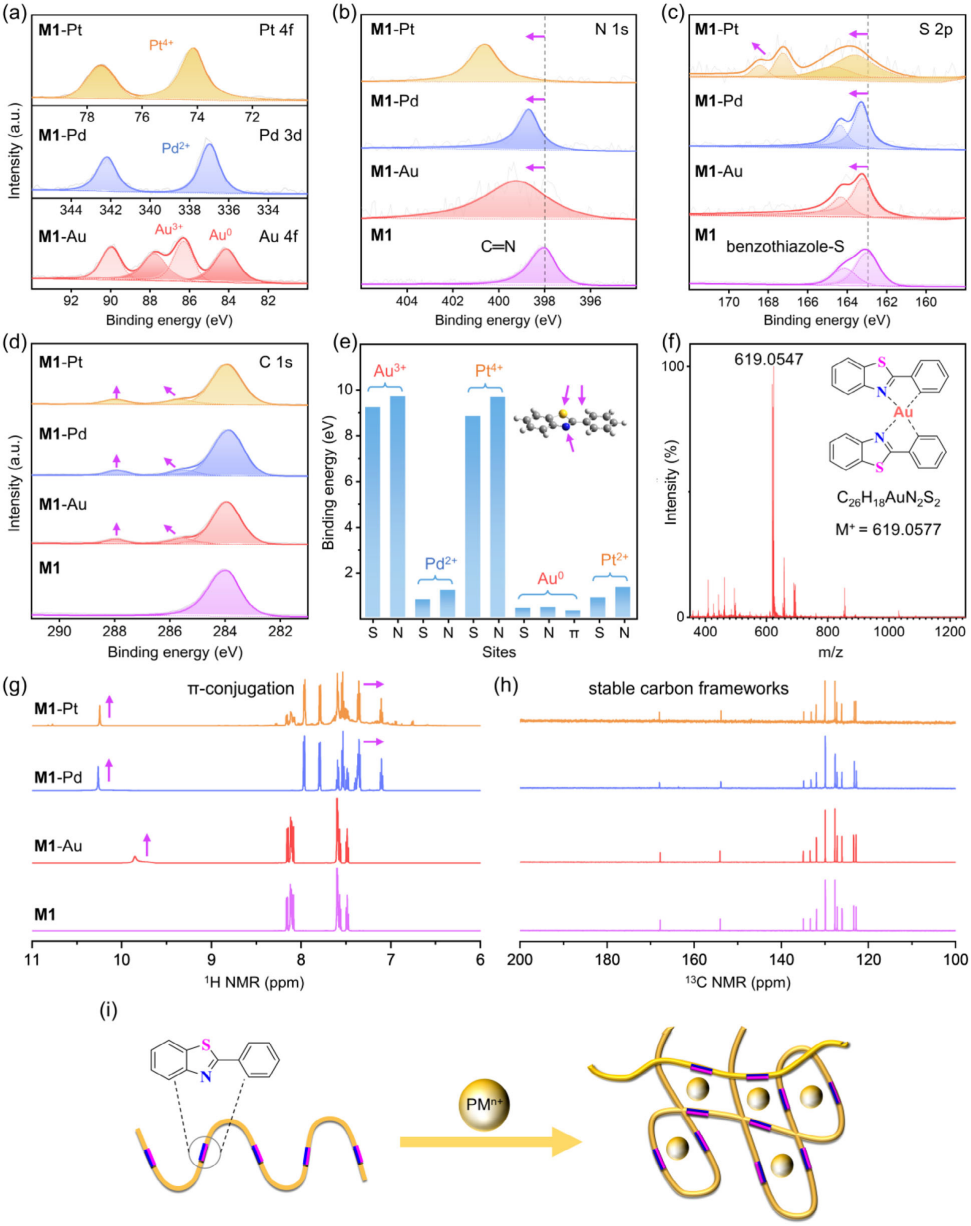
Precious metal coordination of benzothiazole. a–d) XPS of **M1**‐metal complexes (**M1**‐Au, **M1**‐Pd, and **M1**‐Pt): a) Au 4f, Pd 3d, Pt 4f, b) N 1s, c) S 2p, and d) C 1s spectra. e) Metal binding energies on potential sites of **M1** by DFT calculations. f) MALDI‐TOF MS of **M1**‐Au revealing 2:1 ligand‐to‐metal stoichiometry. g) ^1^H NMR and h) ^13^C NMR spectra in DMSO‐*d*_6_. i) Schematic illustration for precious metal ion (PM^n+^) extraction by polybenzothiazoles.

To elucidate the coordination chemistry of benzothiazole, the model compound **M1** was reacted with AuCl_3_, PdCl_2_, and PtCl_4_ in methanol‐water solutions, respectively. XPS analysis of the isolated **M1**‐metal complexes confirmed metal‐specific redox trends (**Figure** **5**a), showing Au^3+^/Au^0^ pairs (86.27/89.96 eV and 84.12/87.76 eV), Pd^2+^ (336.95/342.17 eV), and Pt^4+^ states (74.11/77.48 eV). The N 1s binding energy significantly increased by +1.21 (Au), +0.68 (Pd), and +2.57 (Pt) (Figure 5b), while S 2p_1/2_ exhibited moderate shifts from 164.10 to 164.31 eV (Au), 164.35 eV (Pd), and 164.70 eV (Pt), with Pt^4+^ coordination inducing a high‐oxidation‐state sulfur species (167.25/168.41 eV) (Figure 5c). Emerging C 1s signals at ∼285.5 and ∼287.9 eV suggest π‐mediated interactions in precious metal binding (Figure 5d).^[^ ^46^, ^47^ ^]^ Density functional theory (DFT) calculations quantitatively rationalized these observations (Figure 5e), demonstrating stronger metal binding energies at N sites vs S sites. Notably, Au species exhibit more comparable energies at N/S sites compared to Pd and Pt, reflecting a preference of Au for sulfur, with reduced Au^0^ stabilization via π conjugation (−0.28 eV).^[^ ^48^ ^]^ Moreover, while Au^3+^ and Pt^4+^ exhibited substantial binding energies, Pd^2+^ showed weaker binding, aligning with its attenuated XPS shifts. These results contrast with the typical coordination inertness of sulfur in thiazoles, suggesting that extended electron delocalization in 2‐phenylbenzothiazole may allow sulfur to act as an auxiliary coordination site for stabilizing high‐valent metal ions, while its softer Lewis character could facilitate metal selectivity modulation.^[^ ^49^, ^50^ ^]^

Critical insights emerged from MALDI‐TOF MS and NMR studies. The mass spectra unveiled another metal‐dependent coordination characteristic. The **M1**‐Au measured at 619.0547 g/mol corresponds to a complex containing two **M1** molecules and one Au atom (Figure 5f), while revealing complete dechlorination through ligand exchange. A similar 2:1 stoichiometry was observed for **M1**‐Pt (exp. 618.0667 vs calcd. 618.0632, Figure S21a, Supporting Information), while **M1**‐Pd adopted a unique 2:2 configuration (exp. 636.9082 vs calcd. 636.9058, Figure S21b, Supporting Information), suggesting compensatory dinuclear coordination to overcome weaker single‐site binding affinity. This configuration might be related to Pd–Pd metallophilic interactions induced by the specific coplanar structure of 2‐phenylbenzothiazole.^[^ ^51^, ^52^ ^]^ New downfield‐shifted ^1^H NMR signals (δ 9.40–10.26, Figure 5g), accompanied by distinct perturbations in benzothiazole proton resonances, confirmed π‐assisted coordination. Concurrently, the fully preserved ^13^C NMR framework shown in Figure 5h underscored the fused heterocycle's exceptional redox resilience, with no oxidative ring‐opening observed despite vigorous electron transfer.

Overall, these results establish benzothiazole as a multifunctional chemoresponsive scaffold, redefining the coordination versatility of heterocyclic ligands through a tripartite mechanism: N serves as the primary σ‐coordination anchor, S fine‐tunes selectivity via soft Lewis acid‐base interactions, and the extended π‐conjugation buffers electron transfer while enabling non‐covalent stabilization. The efficient extraction of precious metal ions by **P1** stems from synergistic mechanisms involving hydrophilicity (sulfone‐enhanced), electrostatic interactions, chemical reduction, Lewis acid‐base pairing, and coordination stoichiometry. The extraction begins with protonated N‐sites in benzothiazole motifs electrostatically attracting metal chloroanions under acidic conditions, followed by ion exchange and ligand exchange with deprotonation/dechlorination,^[^ ^11^, ^41^ ^]^ ultimately leading to metal‐N chelation. The observed Au > Pd > Pt hierarchy reflects intrinsic metal differences. For Au^3+^, its higher reduction potential drives reduction and aggregation to Au^0^ nanoclusters,^[^ ^53^, ^54^ ^]^ and its softer Lewis acidity strengthens sulfur interactions. The monovalent [AuCl_4_]^−^ also favors ion exchange over divalent Pd/Pt species.^[^ ^55^ ^]^ Pd and Pt remain ionic during extraction, while the larger ionic radius of [PtCl_6_]^2−^ over [PdCl_4_]^2−^ reduces charge density,^[^ ^56^, ^57^ ^]^ weakening electrostatic attraction. Additionally, Pt binds in 2:1 ligand‐to‐metal stoichiometry, whereas Pd adopts dinuclear 2:2 coordination to enhance its extraction capacity. This synergistic interplay drives spontaneous polymer crosslinking via multinuclear complexation (Figure 5i), with metal‐specific interactions and redox‐coordination pathways enabling exceptional hierarchical metal affinity and selectivity of polybenzothiazoles.

**Figure 6:**
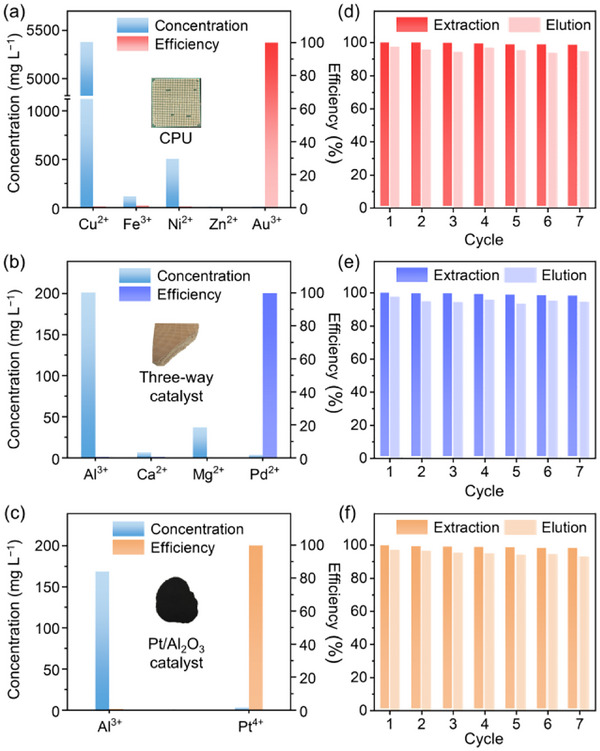
Precious metal recovery from real‐world wastes using **P1** (0.5 g L^−1^). a–c) Concentration and extraction efficiency for aqua regia‐based leachates of a) CPUs, b) three‐way catalysts, and c) Pt/Al_2_O_3_ catalysts. d–f) Extraction‐elution cycles in the leachates of d) CPUs, e) three‐way catalysts, and f) Pt/Al_2_O_3_ catalysts, using an acidic thiourea solution containing 0.05 m thiourea with 5 wt.% HCl for metal elution.

Given the oxidizing nature of high‐valent precious metal ions, the chemical robustness of this heterocycle‐mediated coordination proves important for sustainable metal recovery, particularly in real‐world leaching systems containing aggressive oxidants.^[^ ^58^ ^]^ When employing an uncyclized polythioamide precursor (structurally analogous to **P1**) in concentrated precious metal solutions (3 g L^−1^), extensive desulfurization was observed, as evidenced by powder XRD patterns revealing distinct amorphous‐to‐crystalline sulfur phase transitions (Figure S22, Supporting Information). This starkly contrasts with **P1**’s structural integrity under identical conditions (Figure 4e), which demonstrates polybenzothiazoles’ superior redox resistance. Such stability underpins **P1**’s outstanding recyclability, retaining >98% Au^3+^ extraction efficiency over 11 cycles (Figure S23, Supporting Information, using an acidic thiourea solution containing 0.05 m thiourea with 5 wt.% HCl as the eluent). To further validate real‐world practicality, **P1** was employed to recover targeted precious metals from typical industrial waste sources, including aqua regia‐based leachates of discarded central processing units (CPUs), spent Pd‐containing three‐way catalysts, and spent Pt/Al_2_O_3_ catalysts. Despite high competing metals loads (e.g., Cu from CPUs and Al from catalysts), the polymer achieved >99% extraction efficiency for Au, Pd, and Pt (**Figure** **6**a–c), with distribution coefficients (*K*_d_) for these precious metals exceeding 4.7×10^4^‐fold higher than other competing metals (Table S12, Supporting Information). Critically, stable cycling‐regeneration performance realized in these real leachate systems further underscores its recyclability. The extraction efficiency for precious metals remained above 98% over seven consecutive cycles, while the elution efficiency remained above 93% (Figure 6d–f). Practical metal retrieval was realized by pyrolyzing **P1**‐metal complexes (600–1000 °C in air), yielding >90% pure precious metals as examined by scanning electron microscopy/energy‐dispersive X‐ray spectroscopy (SEM‐EDS, Figure S24, Supporting Information).

## 3 Conclusion

We present a base‐mediated MCP strategy to synthesize polybenzothiazoles with scalable, economical, and structurally tunable advantages. By leveraging alkaline‐activated sulfur and commercially available dianilines, this method circumvents conventional limitations of narrow substrate scope, harsh conditions, and pre‐functionalization procedures, facilely achieving industrially relevant production scales. The resulting polybenzothiazoles exhibit promising precious metal recovery capabilities, with ultrahigh selectivity, rapid kinetics, and robustness across various practical streams. A facile decoupling of conjugation through polar motifs further enhanced functionality, while synergistic electrostatic attraction, N/S coordination, and π‐mediated interactions drive robust and selective benzothiazole‐metal binding, redefining polybenzothiazoles as multifunctional chemoresponsive platforms. This study bridges the gap between economically scalable polymer synthesis and sustainable resource utilization, positioning polybenzothiazoles as pivotal materials for advancing circular economy goals through innovative molecular design and high‐performance metal recovery technologies.

## Conflict of Interest

The authors declare no conflict of interest.

## Author Contributions

H.L. conceived the research. H.Z. carried out all the experiments and data analysis. T.Z. carried out density functional theory (DFT) calculations. M.L. and L.K. helped carry out characterization of materials. H.Z. and X.A. wrote the manuscript. H.L., H.L., and J.Q. directed the project. H.Z., X.A., M.L., L.K., and H.L. revised the manuscript

## Supporting information



Supporting Information

## Data Availability

The data that support the findings of this study are available in the supplementary material of this article.

## References

[1] J. Luke , E. J. Yang , C. Labanti , S. Y. Park , J.‐S. Kim , Nat. Rev. Mater. 2023, 8, 839.

[2] X. H. Zhang , P. Theato , Sulfur‐Containing Polymers, Wiley‐VCH, Hoboken NJ 2021, p. 9783527346707.

[3] D. Fan , D. Wang , J. Zhang , X. Fu , X. Yan , D. Wang , A. Qin , T. Han , B. Z. Tang , J. Am. Chem. Soc. 2024, 146, 17270. 38863213 10.1021/jacs.4c03889

[4] Y. Cui , Q. Fan , H. Feng , T. Li , D. Y. Paraschuk , W. Ma , H. Yan , Energy Environ. Sci. 2024, 17, 8954.

[5] X. Wu , Y. Jiang , N J. Rommelfanger , F. Yang , Qi Zhou , R. Yin , J. Liu , Sa Cai , W. Ren , A. Shin , K S. Ong , K. Pu , G. Hong , Nat. Biomed. Eng. 2022, 6, 754. 35314800 10.1038/s41551-022-00862-w PMC9232843

[6] R. Li , M. Zafar , D. Danovich , V. Subramaniyan , F. Tibika , Y. Tulchinsky , Angew. Chem., Int. Ed. 2024, 63, 202314997. 10.1002/anie.202314997 38009835

[7] M. Deng , J. Chakraborty , G. Wang , K. S. Rawat , L. Bourda , J. Sun , I. Nath , Y. Ji , P. Geiregat , V. Van Speybroeck , X. Feng , P. Van Der Voort , J. Am. Chem. Soc. 2025, 147, 10219. 39992283 10.1021/jacs.4c15825

[8] B. Zhang , H. Gao , Y. Kang , X. Li , Q. Li , P. Zhai , D. Hildebrandt , X. Liu , Y. Wang , S. Qiao , Adv. Sci. 2024, 11, 2308535. 10.1002/advs.202308535 PMC11095168 38454537

[9] G. Wang , S. Yang , Na Y Kang , M. Lu , B. Hua , H. Wei , J. Kang , W. Tang , Y. M. Lee , J. Membr. Sci. 2023, 668, 121239.

[10] Q.‐X. Ma , L. Xu , Y. Fan , L. Wang , J.‐N. Xu , J. Zhao , X.‐B. Chen , Small 2024, 20, 2406912.39324225

[11] X. Li , Y.‐L. Wang , J. Wen , L. Zheng , C. Qian , Z. Cheng , H. Zuo , M. Yu , J. Yuan , R. Li , W. Zhang , Y. Liao , Nat. Commun. 2023, 14, 263. 36650177 10.1038/s41467-023-35971-w PMC9845340

[12] J. Shi , S.‐Q. Peng , B. Kuang , S. Wang , Y. Liu , J.‐X. Zhou , X. Li , M.‐H. Huang , Adv. Mater. 2024, 36, 2405731. 10.1002/adma.202405731 38857110

[13] Y. Hong , D. Thirion , S. Subramanian , M. Yoo , H. Choi , H. Y. Kim , J. F. Stoddart , C. T. Yavuz , Proc. Natl. Acad. Sci. USA 2020, 117, 16174. 32571947 10.1073/pnas.2000606117 PMC7368251

[14] L. Rocchigiani , M. Bochmann , Chem. Rev. 2021, 121, 8364. 32966741 10.1021/acs.chemrev.0c00552

[15] Y. Haketa , K. Yamasumi , H. Maeda , Chem. Soc. Rev. 2023, 52, 7170. 37795542 10.1039/d3cs00581j

[16] J.‐R. Pouliot , F. Grenier , J. T. Blaskovits , S. Beaupré , M. Leclerc , Chem. Rev. 2016, 116, 14225. 27809495 10.1021/acs.chemrev.6b00498

[17] J. Qian , X. Zhang , Y. Jia , H. Xu , B. Pan , Environ. Sci. Technol. 2025, 59, 1060. 39761191 10.1021/acs.est.4c10073

[18] H. Xiong , Q. Lin , Y. Lu , D. Zheng , Y. Li , S. Wang , W. Xie , C. Li , X. Zhang , Y. Lin , Z.‐X. Wang , Q. Shi , T. J. Marks , H. Huang , Nat. Mater. 2024, 23, 695. 38287128 10.1038/s41563-023-01794-9

[19] P. J. Waller , Y. S. AlFaraj , C. S. Diercks , N. N. Jarenwattananon , O. M. Yaghi , J. Am. Chem. Soc. 2018, 140, 9099. 29999317 10.1021/jacs.8b05830

[20] G. Wang , S. Yang , N. Y. Kang , B. Hua , M. Lu , H. Wei , J. Kang , W. Tang , Y. M. Lee , Macromolecules 2023, 56, 5546.

[21] D. Liu , H. Zhang , L. Zhang , J. Wang , Z. Chang , H. Cong , Polym. Chem. 2024, 15, 2408.

[22] T.‐J. Yue , W.‐M. Ren , X.‐B. Lu , Chem. Rev. 2023, 123, 14038. 37917384 10.1021/acs.chemrev.3c00437

[23] X. Che , J. Jiang , F. Xiao , H. Huang , G.‐J. Deng , Org. Lett. 2017, 19, 4576. 28817291 10.1021/acs.orglett.7b02168

[24] X. Zhu , Y. Yang , G. Xiao , J. Song , Y. Liang , G. Deng , Chem. Commun. 2017, 53, 11917. 10.1039/c7cc07366f 29044251

[25] F. Haase , E. Troschke , G. Savasci , T. Banerjee , V. Duppel , S. Dörfler , M M. J. Grundei , A M. Burow , C. Ochsenfeld , S. Kaskel , B V. Lotsch , Nat. Commun. 2018, 9, 2600. 29968723 10.1038/s41467-018-04979-y PMC6030076

[26] K. Wang , Z. Jia , Y. Bai , X. Wang , S. E. Hodgkiss , L. Chen , S. Y. Chong , X. Wang , H. Yang , Y. Xu , F. Feng , J. W. Ward , A. I. Cooper , J. Am. Chem. Soc. 2020, 142, 11131. 32475114 10.1021/jacs.0c03418

[27] T. B. Nguyen , Adv. Synth. Catal. 2020, 362, 3448.

[28] J. Kim , K. Oh , Adv. Synth. Catal. 2020, 362, 3576.

[29] B. Chatterjee , D. Mondal , S. Bera , Beilstein J. Org. Chem. 2024, 20, 1376. 38919603 10.3762/bjoc.20.120 PMC11196959

[30] W. Cao , F. Dai , R. Hu , B. Z. Tang , J. Am. Chem. Soc. 2020, 142, 978. 31841620 10.1021/jacs.9b11066

[31] Y. Hu , L. Zhang , Z. Wang , R. Hu , B. Z. Tang , Polym. Chem. 2023, 14, 2617.

[32] X.‐D. Hu , S. E. Jenkins , B. G. Min , M. B. Polk , S. Kumar , Macromol. Mater. Eng. 2003, 288, 823.

[33] T. Guntreddi , R. Vanjari , K. N. Singh , Org. Lett. 2014, 16, 3624. 24978059 10.1021/ol501482g

[34] T. Kanbara , Y. Kawai , K. Hasegawa , H. Morita , T. Yamamoto , J. Polym. Sci., Part A: Polym. Chem. 2001, 39, 3739.

[35] Y. Huang , Y. Yu , R. Hu , B. Z. Tang , J. Am. Chem. Soc. 2024, 146, 14685. 38717074 10.1021/jacs.4c02155

[36] J. Jiang , G. Li , F. Zhang , H. Xie , G.‐J. Deng , Adv. Synth. Catal. 2018, 360, 1622.

[37] M. Singh , P. Vaishali , A. K. Paul , V. Singh , Org. Biomol. Chem. 2020, 18, 4459. 10.1039/d0ob00888e 32490470

[38] X. Wang , L. Chen , S. Y. Chong , M. A. Little , Y. Wu , W.‐H. Zhu , R. Clowes , Y. Yan , M. A. Zwijnenburg , R. S. Sprick , A. I. Cooper , Nat. Chem. 2018, 10, 1180. 30275507 10.1038/s41557-018-0141-5

[39] W. Zhang , X. Luo , H. Tang , Z. Tang , F. Huang , Q. Wan , G. Yu , H. Yang , J. Mater. Chem. A 2025, 13, 4746.

[40] F. Li , J. Zhu , P. Sun , M. Zhang , Z. Li , D. Xu , X. Gong , X. Zou , A. K. Geim , Y. Su , H.‐M. Cheng , Nat. Commun. 2022, 13, 4472. 35918342 10.1038/s41467-022-32204-4 PMC9345893

[41] S. S. Shin , Y. Jung , S. Jeon , S.‐J. Park , S.‐J. Yoon , K.‐W. Jung , J.‐W. Choi , J.‐H. Lee , Nat. Commun. 2024, 15, 3889. 38719796 10.1038/s41467-024-48090-x PMC11079046

[42] L. MacDonald , D. Zhang , A. Karamalidis , Resour. Conserv. Recycl. 2024, 205, 107590.

[43] A. Zupanc , J. Install , M. Jereb , T. Repo , Angew. Chem., Int. Ed. 2023, 62, 202214453. 10.1002/anie.202214453 PMC10107291 36409274

[44] Z. Wang , X. Luo , Z. Song , K. Lu , S. Zhu , Y. Yang , Y. Zhang , W. Fang , J. Jin , Nat. Commun. 2022, 13, 4169. 35853846 10.1038/s41467-022-31575-y PMC9296620

[45] T. Ye , X. Zhang , W. Xu , H. Li , K. Tang , Chem. Eng. J. 2024, 498, 155695.

[46] J. Jiang , J. Kou , Q. Wu , L. Chen , Geng , G. Shan , C. Sun , Z. Su , X. Wang , Angew. Chem., Int. Ed. 2025, 64, 202410665. 10.1002/anie.202410665 39825671

[47] H. Jia , N. Yao , Y. Jin , L. Wu , J. Zhu , W. Luo , Nat. Commun. 2024, 15, 5419. 38926414 10.1038/s41467-024-49834-5 PMC11208516

[48] D. Yang , Y. Wu , Z. Yuan , C. Zhou , Y. Dai , X. Wan , Y. Zhu , Y. Yang , Sci. China Chem. 2024, 67, 806.

[49] L. M. T. Frija , A. J. L. Pombeiro , M. N. Kopylovich , Coord. Chem. Rev. 2016, 308, 32.

[50] J. Stenger‐Smith , I. Chakraborty , P. K. Mascharak , J. Inorg. Biochem. 2018, 185, 80. 29800748 10.1016/j.jinorgbio.2018.05.003

[51] M. Loni , Y. Balmohammadi , R. Dadgar Yeganeh , K. Imani , B. Notash , A. Bazgir , New J. Chem. 2021, 45, 3290.

[52] Q. Wan , J. Yang , W.‐P. To , C.‐M. Che , *Proc. Natl. Acad. Sci*. USA 2021, 118, 2019265118.

[53] P. R. Zalupski , R. McDowell , G. Dutech , Solvent Extr. Ion Exch. 2014, 32, 737.

[54] Y. Su , A. Berbille , X.‐F. Li , J. Zhang , M. PourhosseiniAsl , H. Li , Z. Liu , S. Li , J. Liu , L. Zhu , Z. L. Wang , Nat. Commun. 2024, 15, 4196. 38760357 10.1038/s41467-024-48407-w PMC11101412

[55] W. Wei , C.‐W. Cho , S. Kim , M.‐H. Song , J. K. Bediako , Y.‐S. Yun , J. Mol. Liq. 2016, 216, 18.

[56] W. Wei , D. H. K. Reddy , J. K. Bediako , Y.‐S. Yun , Chem. Eng. J. 2016, 289, 413.

[57] S. Lin , J. K. Bediako , C.‐W. Cho , M.‐H. Song , Y. Zhao , J.‐A. Kim , J.‐W. Choi , Y.‐S. Yun , Chem. Eng. J. 2018, 345, 337.

[58] S. Mir , N. Dhawan , Resour. Conserv. Recycl. 2022, 178, 106027.

