# Supplementary material for: Base‐Mediated Scalable Synthesis of Polybenzothiazoles: Fused‐Heterocycle‐Engineered Recovery of Precious Metals
Source: Adv Sci (Weinh). 2025 Aug 13;12(35):e06580. doi: 10.1002/advs.202506580 (PMC12462968; doi:10.1002/advs.202506580)
Supplement: Supplementary file 1 — Supporting Information [file ADVS-12-e06580-s001.pdf]

ADVANCED SCIENCE
Open Access

Supporting Information

for *Adv. Sci.*, DOI 10.1002/advs.202506580

Base-Mediated Scalable Synthesis of Polybenzothiazoles: Fused-Heterocycle-Engineered Recovery of Precious Metals

Hongjie Zhou, Xiaoqiang An, Tianshu Zhang, Mingran Li, Lingru Kong, Huachun Lan, Huijuan Liu and Jiuhui Qu*

Supporting Information

Base-Mediated Scalable Synthesis of Polybenzothiazoles: Fused-Heterocycle-Engineered Recovery of Precious Metals

Hongjie Zhou, Xiaoqiang An, Tianshu Zhang, Mingran Li, Lingru Kong, Huachun Lan, Huijuan Liu, and Jiuhui Qu*

Center for Water and Ecology, State Key Laboratory of Iron and Steel Industry Environmental Protection, School of Environment, Tsinghua University, Beijing 100084, China

*Corresponding author E-mail: hclan@tsinghua.edu.cn (H. Lan)

Table of contents

Experimental procedures....3

1. General information 3

Results and discussion 3

2. Optimization of polymerization conditions....3

3. Synthesis of polymers and compounds....6

4. Precious metal extraction performances....15

5. Extraction mechanism....23

6. Stability and practical metal recovery 25

7. Original ^{1}H and ^{13}C NMR spectra....28

References....44

Experimental procedures

1. General information

All reagents were purchased from commercial sources and used without further purification. Unless otherwise noted, all reactions were conducted in oven- or flame-dried glassware. ^{1}H and ^{13}C NMR spectra were recorded on a Bruker Avance 600 MHz NMR spectrometer using deuterated dimethyl sulfoxide (DMSO-d_6) as solvent and tetramethylsilane (TMS, δ = 0) as internal reference. IR spectra were determined on a Thermo Fisher Nicolet iS50 FT-IR spectrometer. The number (M_n) and weight (M_w) average molecular weights and polydispersity indices ($Đ = M_w/M_n$) of the polymers were determined by a Waters 1515 gel permeation chromatography (GPC) system. DMF/LiBr solution (0.05 M LiBr) was used as eluent at a flow rate of 1 mL/min. A set of monodisperse polystyrenes, covering the M_w range of 10^3–10^7 g/mol, were utilized as standards for molecular weight calibration. Thermogravimetric analysis was carried out on TA TGA Q5000 and DSC Q2000 under a nitrogen atmosphere at a heating rate of 10°C/min. Water contact angles were measured by JY-82C video optical contact angle meter (Dingsheng, China). Powder X-ray diffraction (XRD) patterns were collected on a Bruker D8 Advance X-ray diffractometer with Cu K_α radiation. Transmission electron microscope (TEM) images were obtained on a FEI Talos F200x microscope. Aberration-correction high-angel annular dark field scanning transmission electron microscopy (AC-HAADF-STEM) images, and the mapping were recorded on a Super-X EDS system. X-ray photoelectron spectroscopy (XPS) was performed using a Thermal Fisher Scientific Escalab 250Xi spectrometer with a monochromatic Al K_α X-ray source. Field emission scanning electron microscopy (FESEM) coupled with energy-dispersive spectroscopy (EDS) was conducted on Zeiss GeminiSEM 500. UV–Vis absorbance spectra were recorded on a Hitachi U3900 spectrophotometer. Matrix-assisted laser desorption/ionization time of flight mass spectrometry (MALDI-TOF MS) analysis was performed using Shimadzu AXIMA Performance MALDI-TOF MS. All extraction experiments were conducted with more than three parallel trials. Metal ion concentrations were analyzed by Agilent 5110 inductively coupled plasma-optical emission spectrometry (ICP-OES), while trace concentrations were analyzed by Agilent 7800 inductively coupled plasma-mass spectrometry (ICP-MS).

Results and discussion

2. Optimization of polymerization conditions

Table S1. Screening of bases

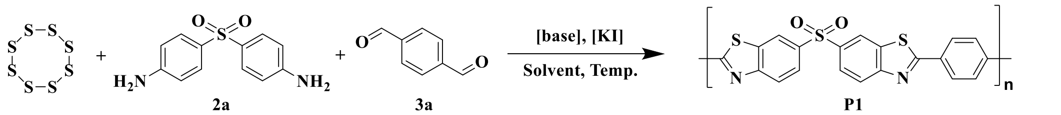

Entry[a]	Base	Yield (%)[b]	M_w (kDa)	$Đ$ (M_w/M_n)
1	-	16	6.6	1.14
2	Na_2CO_3	34	9.8	1.11
3	KOH	57	15.4	1.15
4	K_2CO_3	38	11.3	1.21
5	Cs_2CO_3	46	14.4	1.20
6	KF	50	10.4	1.12
7	CsF	52	12.4	1.17
8	*t*-BuOK	45	12.1	1.15
9	Et_3N	28	7.5	1.17
10	DBU	41	11.2	1.16
11	DABCO	31	8.4	1.10

[a] Conducted at 120°C in DMSO under air for 12 h. [**2a**] = 0.5 M. [KI] = 0.1 M. 1/8[S_8]:[**2a**]:[**3a**] = 2.0:1.0:1.0. [Base] = 0.5 M. [b] Isolated yield was calculated based on the dialdehyde monomers.

Table S2. Screening of KOH concentrations

Entry[a]	Concentration	Yield (%)[b]	M_w (kDa)	$Đ$ (M_w/M_n)
1	0.2 M	34	5.8	1.14
2	0.5 M	57	15.4	1.15
3	1 M	71	16.7	1.38
4	1.5 M	66	10.1	1.17

[a] Conducted at 120°C in DMSO under air for 12 h. [**2a**] = 0.5 M. [KI] = 0.1 M. 1/8[S_8]:[**2a**]:[**3a**] = 2.0:1.0:1.0. [b] Isolated yield was calculated based on the dialdehyde monomers.

Table S3. Screening of solvents

Entry[a]	Solvent	Yield (%)[b]	M_w (kDa)	$Đ$ (M_w/M_n)
1	DMSO	71	16.7	1.38
2	NMP	67	16.9	1.16
3	DMF	62	11.8	1.13
4	DMAc	58	10.6	1.38
5	NMP/DMSO (3:1)[c]	73	20.5	1.02
6	NMP/DMSO (1:1)	69	19.6	1.05
7	NMP/DMSO (1:3)	70	18.9	1.27

[a] Conducted at 120°C under air for 12 h. [**2a**] = 0.5 M. [KI] = 0.1 M. 1/8[S_8]:[**2a**]:[**3a**] = 2.0:1.0:1.0. [KOH] = 1 M. [b] Isolated yield was calculated based on the dialdehyde monomers. [c] NMP/DMSO (v/v) = 3:1.

Table S4. Screening of monomer loading ratios

Entry[a]	1/8[S_8]:[**2a**]:[**3a**]	Yield (%)[b]	M_w (kDa)	$Đ$ (M_w/M_n)
1	2.0: 1.0: 1.0	73	18.9	1.27
2	3.0: 1.0: 1.0	78	21.4	1.14
3	4.0: 1.0: 1.0	81	24.8	1.11
4	4.0: 1.5: 1.0	85	25.6	1.18
5	4.0: 2.0: 1.0	88	27.0	1.16
6	4.0: 3.0: 1.0	86	27.7	1.21
7	3.0: 2.0: 1.0	83	21.8	1.13
8	2.0: 0.5: 1.0	65	19.6	1.26

[a] Conducted at 120°C under air in NMP/DMSO (v/v = 3:1) for 12 h. [**3a**] = 0.5 M. [KI] = 0.1 M. [KOH] = 1 M. [b] Isolated yield was calculated based on the dialdehyde monomers.

Table S5. Screening of monomer concentrations

Entry[a]	[**3a**] (M)	Yield (%)[b]	M_w (kDa)	$Đ$ (M_w/M_n)
1	0.1	39	7.1	1.05
2	0.3	73	11.8	1.13
3	0.5	88	27.0	1.16
4	0.7	86	29.4	1.10
5	1	75	27.5	1.11

[a] Conducted at 120°C under air in NMP/DMSO (v/v = 3:1) for 12 h. 1/8[S_8]:[**2a**]:[**3a**] = 4.0:2.0:1.0. [KI] = 20%[**3a**]. [KOH] = 1 M. [b] Isolated yield was calculated based on the dialdehyde monomers.

Table S6. Screening of reaction times and temperatures

Entry[a]	Temperature (°C)	Time (h)	Yield (%)[b]	M_w (kDa)	$Đ$ (M_w/M_n)
1	120	12	88	27.0	1.16
2	130	12	90	30.5	1.11
3	140	12	89	32.2	1.58
4	130	24	93	37.6	1.18
5	130	36	92	37.2	1.23

[a] Conducted under air in NMP/DMSO (v/v = 3:1). [**3a**] = 0.5 M. 1/8[S_8]:[**2a**]:[**3a**] = 4.0:2.0:1.0. [KI] = 0.1 M. [KOH] = 1 M. [b] Isolated yield was calculated based on the dialdehyde monomers.

3. Synthesis of polymers and compounds

3.1 General synthesis of polybenzothiazoles

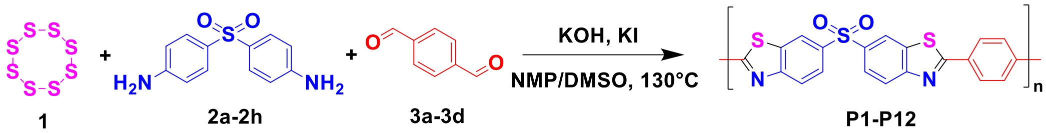

A 10 mL oven-dried reaction vessel was charged with aromatic diamine (2.0 mmol), aromatic dialdehyde (1.0 mmol), S_8 (4.0 mmol), KI (0.2 mmol), KOH (2.0 mmol), NMP (1.5 mL) and DMSO (0.5 mL). The reaction vessel was stirred under air at 130°C for 24 h. After cooling to room temperature, the reaction mixture was precipitated by dropping it into 50 mL of methanol through a cotton filter. The resulting precipitate was collected and washed with methanol (3 × 20 mL). For polymer products with low solubility, the precipitates formed after the reaction were filtered and washed three times with DMF, followed by three washes with methanol. The obtained solids were dried under vacuum at 60°C to a constant weight.

P1. A crimson-red solid was obtained in 93% yield. M_w = 37.6 kDa, $Đ$ = 1.18. IR (KBr disk, cm^{-1}): 553, 575, 619 (C–S), 697, 831, 1116, 1300, 1402, 1501, 1523, 1592, 1625, 1664. ^1H NMR (600 MHz, DMSO-d_6), δ (TMS, ppm): 8.06 – 7.82 (m, 6H), 7.56 – 7.40 (m, 2H), 6.61 – 6.54 (m, 2H). ^{13}C NMR (150 MHz, DMSO-d_6), δ (TMS, ppm): 174.31, 154.16, 153.07, 129.98, 129.05, 127.73, 125.87, 124.70, 120.77, 113.56.

P2. A brown solid was obtained in 79% yield. M_w = 24.3 kDa, $Đ$ = 1.08. IR (KBr disk, cm^{-1}): 570, 619 (C–S), 693, 829, 1010, 1112, 1148, 1300, 1353, 1403, 1499, 1526, 1592, 1622, 1661. ^1H NMR (600 MHz, DMSO-d_6), δ (TMS, ppm): 8.13 – 7.87 (m, 4H), 7.59 – 7.45 (m, 2H), 6.66 – 6.58 (m, 2H). ^{13}C NMR (150 MHz, DMSO-d_6), δ (TMS, ppm): 174.28, 154.23, 143.20, 130.01, 129.03, 128.64, 127.65, 126.35, 125.47, 113.57, 113.33.

P3. A black solid was obtained in 98% yield. M_w = 31.0 kDa, $Đ$ = 1.11. IR (KBr disk, cm^{-1}): 588, 617 (C–S), 646, 688, 731, 1000, 1154, 1319, 1374, 1435, 1517, 1586, 1715. ^1H NMR (600 MHz, DMSO-d_6), δ (TMS, ppm): 8.40 – 8.11 (m, 3H), 8.10 – 7.87 (m, 6H). ^{13}C NMR (150 MHz, DMSO-d_6), δ (TMS, ppm): 174.29, 165.61, 162.60, 162.53, 148.68, 143.03, 140.68, 136.71, 128.96, 126.40, 121.27.

P4. A yellow solid was obtained in 96% yield. M_w = 39.6 kDa, $Đ$ = 1.25. IR (KBr disk, cm^{-1}): 555, 618 (C–S), 695, 829, 1105, 1146, 1299, 1402, 1503, 1593, 1626, 1694. ^1H NMR (600 MHz, DMSO-d_6), δ (TMS, ppm): 8.54 – 8.18 (m, 2H), 8.10 – 7.46 (m, 6H), 6.84 – 6.67 (m, 2H). ^{13}C NMR (150 MHz, DMSO-d_6), δ (TMS, ppm): 174.34, 165.97, 153.56, 135.14, 131.75, 129.76, 128.88, 128.16, 120.88, 113.81.

P5. An orange solid was obtained in 88% yield. M_w = 28.1 kDa, $Đ$ = 1.14. IR (KBr disk, cm^{-1}): 553, 622 (C–S), 677, 771, 853, 929, 1009, 1148, 1282, 1313, 1406, 1515, 1595. ^1H NMR (600 MHz, DMSO-d_6), δ (TMS, ppm): 8.43 – 7.98 (m, 4H), 7.67 (d, J = 8.2 Hz, 2H), 7.59 – 7.41 (m, 4H). ^{13}C NMR (150 MHz, DMSO-d_6), δ (TMS, ppm): 193.02 (C=O), 174.30, 155.55, 154.18,

142.81, 133.07, 132.28, 130.58, 130.01, 129.98, 124.37, 123.69, 113.23, 113.20.

P6. A yellow solid was obtained in 91% yield. M_w =29.4 kDa, $Đ$ = 1.08. IR (KBr disk, cm^{-1}): 620 (C–S), 693, 832, 1116, 1213, 1243, 1449, 1497, 1599, 1618, 1664. ^{1}H NMR (600 MHz, DMSO-d_6), δ (TMS, ppm): 8.21 – 7.56 (m, 4H), 7.23 – 6.64 (m, 6H). ^{13}C NMR (150 MHz, DMSO-d_6), δ (TMS, ppm): 174.28, 167.64, 161.99, 159.85, 153.84, 149.30, 140.40, 134.07, 134.03, 125.71, 123.51, 122.88, 122.51, 121.34, 120.01, 119.56, 119.26, 119.14, 119.02.

P7. A brown solid was obtained in 96% yield. M_w = 27.4 kDa, $Đ$ = 1.15. IR (KBr disk, cm^{-1}): 620 (C–S), 818, 1119, 1303, 1493, 1595, 1662. ^{1}H NMR (600 MHz, DMSO-d_6), δ (TMS, ppm): 8.10 – 7.69 (m, 2H), 7.36 – 7.00 (m, 6H), 6.68 (s, 2H). ^{13}C NMR (150 MHz, DMSO-d_6), δ (TMS, ppm): 174.29, 165.84, 151.94, 150.07, 136.76, 136.29, 136.25, 132.94, 130.18, 130.14, 126.11, 125.17, 123.84, 115.81.

P8. A yellow solid was obtained in 90% yield. M_w = 28.3 kDa, $Đ$ = 1.30. IR (KBr disk, cm^{-1}): 620 (C–S), 696, 810, 921, 1016, 1125, 1182, 1281, 1370, 1512, 1591, 1621, 1665. ^{1}H NMR (600 MHz, DMSO-d_6), δ (TMS, ppm): 8.59 – 7.92 (m, 4H), 7.87 – 7.10 (m, 6H), 4.15 – 3.71 (m, 2H). ^{13}C NMR (150 MHz, DMSO-d_6), δ (TMS, ppm): 174.29, 149.42, 136.58, 126.42, 121.44, 121.38, 119.55, 119.08, 116.88, 116.17.

P9. A crimson-red solid was obtained in 92% yield. M_w = 24.8 kDa, $Đ$ = 1.09. IR (KBr disk, cm^{-1}): 516, 618 (C–S), 674, 828, 963, 1014, 1116, 1251, 1362, 1510, 1599, 1651. ^{1}H NMR (600 MHz, DMSO-d_6), δ (TMS, ppm): 7.89 – 7.76 (m, 4H). ^{13}C NMR (150 MHz, DMSO-d_6), δ (TMS, ppm): 186.31, 174.30, 163.05, 153.84, 137.77, 125.77, 125.38, 122.63, 120.85.

P10. A brown solid was obtained in 81% yield. M_w = 12.0 kDa, $Đ$ = 1.38. IR (KBr disk, cm^{-1}): 618 (C–S), 674, 828, 963, 1014, 1116, 1251, 1362, 1510, 1599, 1651. ^{1}H NMR (600 MHz, DMSO-d_6), δ (TMS, ppm): 8.61 – 7.51 (m, 4H), 7.27 – 6.67 (m, 2H). Solid-state ^{13}C NMR (150 MHz): 167.71, 164.95, 151.79, 146.98, 135.10, 127.68, 115.85, 107.13.

P11. A crimson-red solid was obtained in 73% yield. M_w = 11.0 kDa, $Đ$ = 1.42. IR (KBr disk, cm^{-1}): 520, 624 (C–S), 848, 963, 1014, 1125, 1193, 1304, 1361, 1414, 1488, 1563, 1614, 1694. ^{1}H NMR (600 MHz, DMSO-d_6), δ (TMS, ppm): 8.28 – 7.42 (m, 4H), 7.20 – 6.57 (m, 2H). Solid-state ^{13}C NMR (150 MHz): 167.89, 153.98, 145.42, 127.95, 115.50, 107.49.

P12. A brown solid was obtained in 88% yield. M_w = 49.9 kDa, $Đ$ = 1.96. IR (KBr disk, cm^{-1}): 617 (C–S), 647, 749, 843, 961, 1019, 1124, 1247, 1302, 1403, 1465, 1605, 1668. ^{1}H NMR (600 MHz, DMSO-d_6), δ (TMS, ppm): 8.82 – 8.49 (m, 2H), 8.10 – 7.85 (m, 4H), 7.45 (d, J = 23.3 Hz, 2H), 7.26 – 6.64 (m, 6H). ^{13}C NMR (150 MHz, DMSO-d_6), δ (TMS, ppm): 174.28, 162.06, 160.68, 153.29, 148.56, 137.05, 135.72, 132.21, 130.40, 130.38, 129.92, 128.64, 123.62, 121.96, 121.68, 118.26, 117.84, 117.04, 115.73.

Table S7. Synthesis of diverse polybenzothiazoles

Entry[a]	Polymer	Monomer	Yield (%)[b]	M_w (kDa)	$Đ$ (M_w/M_n)
1	**P1**	**1** + **2a** + **3a**	93	37.6	1.18
2	**P2**	**1** + **2a** + **3b**	79	24.3	1.08
3	**P3**	**1** + **2a** + **3c**	98	31.0	1.11
4	**P4**	**1** + **2a** + **3d**	96	39.6	1.25
5	**P5**	**1** + **2b** + **3d**	88	28.1	1.14
6	**P6**	**1** + **2c** + **3d**	91	29.4	1.08
7	**P7**	**1** + **2d** + **3d**	96	27.4	1.15
8	**P8**	**1** + **2e** + **3d**	90	28.3	1.30
9	**P9**	**1** + **2f** + **3b**	92	24.8	1.09
10[c]	**P10**	**1** + **2f** + **3a**	81	12.0	1.38
11[c]	**P11**	**1** + **2g** + **3a**	73	11.0	1.42
12	**P12**	**1** + **2h** + **3a**	88	49.9	1.96

[a] Conducted at 130°C under air in NMP/DMSO (v/v = 3:1) for 24 h. [**3a**–**3d**] = 0.5 M. 1/8[S_8]:[**2a**–**2h**]:[**3a**–**3d**] = 4.0:2.0:1.0. [KI] = 0.1 M. [KOH] = 1 M. [b] Isolated yield was calculated based on the dialdehyde monomers. [c] [KOH] = 1.5 M.

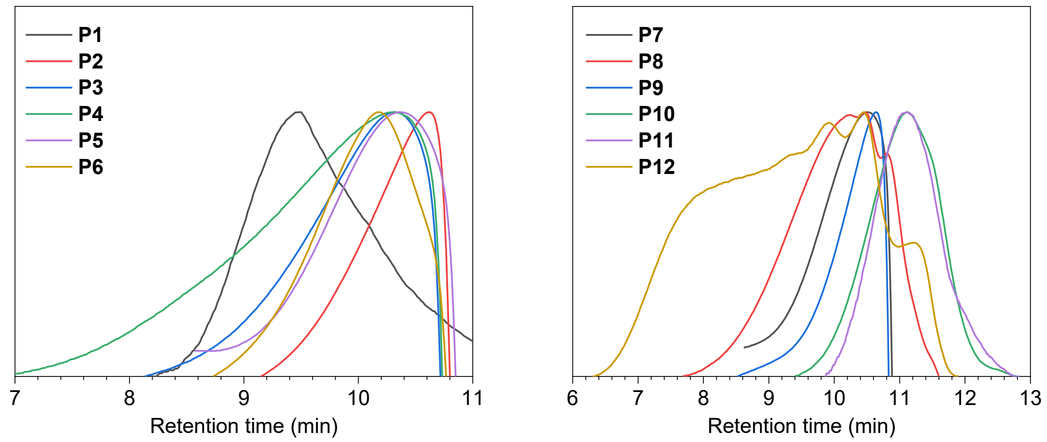

Figure S1. GPC curves of polybenzothiazoles.

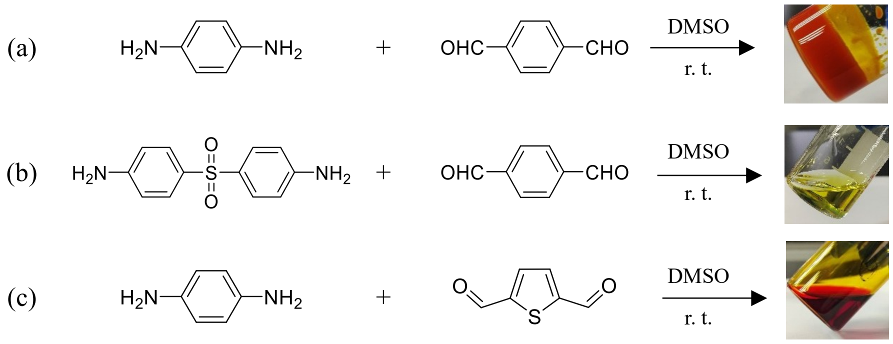

Figure S2. Generation of Schiff-base intermediates from aromatic diamines and dialdehydes. (a) Reaction of **2f** and **3a** in DMSO forms insoluble polyimines. (b) Reaction of **2a** and **3a** in DMSO. (c) Reaction of **2f** and **3b** in DMSO.

3.2 Synthesis of model compound M1

A 10 mL oven-dried reaction vessel was charged with benzaldehyde (1.0 mmol), aniline (1.2 mmol), S_8 (4.5 mmol), KOH (1.0 mmol), KI (0.2 mmol), NMP (1.5 mL) and DMSO (0.5 mL). The reaction vessel was stirred under air at 130°C for 24 h. After cooling to room temperature, the volatiles were removed under reduced pressure. The residue was purified by column chromatography on silica gel (petroleum ether/EtOAc = 300:1, v/v) to afford the desired product **M1** as an off-white solid (154.1 mg, 73% yield). IR (KBr disk, cm^{-1}): 551, 623 (C–S), 687, 728, 767, 914, 963, 998, 1028, 1071, 1159, 1225, 1258, 1284, 1314, 1433, 1457, 1479, 1509, 1555. ^{1}H NMR (600 MHz, DMSO-d_6), δ (TMS, ppm): 8.08 (d, J = 7.9 Hz, 1H), 8.06 – 7.99 (m, 3H), 7.54 – 7.46 (m, 4H), 7.41 (t, J = 7.5 Hz, 1H). ^{13}C NMR (150 MHz, DMSO-d_6), δ (TMS, ppm): 167.80, 154.10, 134.99, 133.39, 131.91, 130.01, 129.90, 127.71, 127.17, 126.05, 123.42, 122.85. MALDI-TOF MS: m/z 212.0567 (M + H^+, calcd. 212.0528).

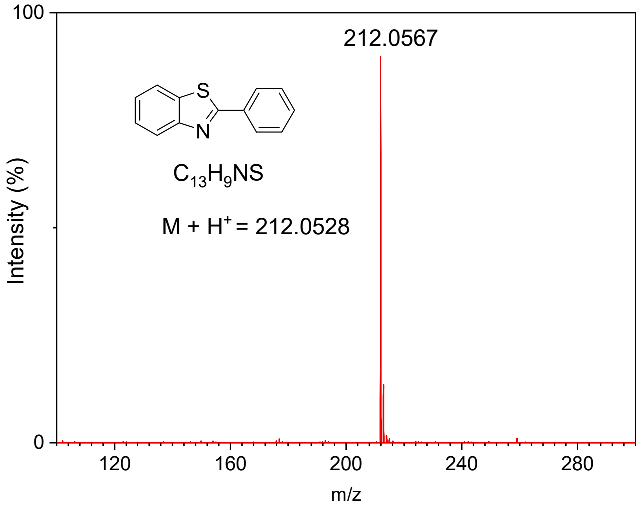

Figure S3. MALDI-TOF MS spectrum of **M1**.

3.3 Synthesis of polythioamide (PTA)

Sublimed sulfur (**1**, 4.0 mmol), 4,4′-diaminodiphenyl sulfone (**2a**, 2.0 mmol), terephthalaldehyde (**3a**, 1.0 mmol), and KOH (1 mmol) were reacted in 2 mL of DMF under nitrogen at 110°C for 8 h in a 10 mL Schlenk tube equipped with a magnetic stir bar. After cooling to room temperature, the reaction mixture was diluted with 2 mL of DMF and precipitated by dropping it into 50 mL of methanol through a cotton filter. The resulting precipitates were filtered and washed three times with methanol (3 × 20 mL). A yellow solid was obtained after drying under vacuum at 60°C to a constant weight. IR (KBr disk, cm^{-1}): 573, 695, 726, 825, 889, 1011, 1070, 1107, 1200, 1143, 1296, 1406, 1506, 1590, 1626, 1692, 3369. ^{1}H NMR (600 MHz, DMSO-d_6), δ (TMS, ppm): 11.85 (C=S, d, J = 18.5 Hz, 2H), 8.27 – 7.95 (m, 2H), 7.87 – 7.54 (m, 4H), 7.31 (dd, J = 54.7, 8.4 Hz, 4H), 6.92 (d, J = 8.4 Hz, 2H). ^{13}C NMR (150 MHz, DMSO-d_6), δ (TMS, ppm): 193.22 (C=S), 153.20, 137.23, 135.19, 130.67, 129.93, 129.04, 128.64, 128.43, 122.33, 113.55, 113.35.

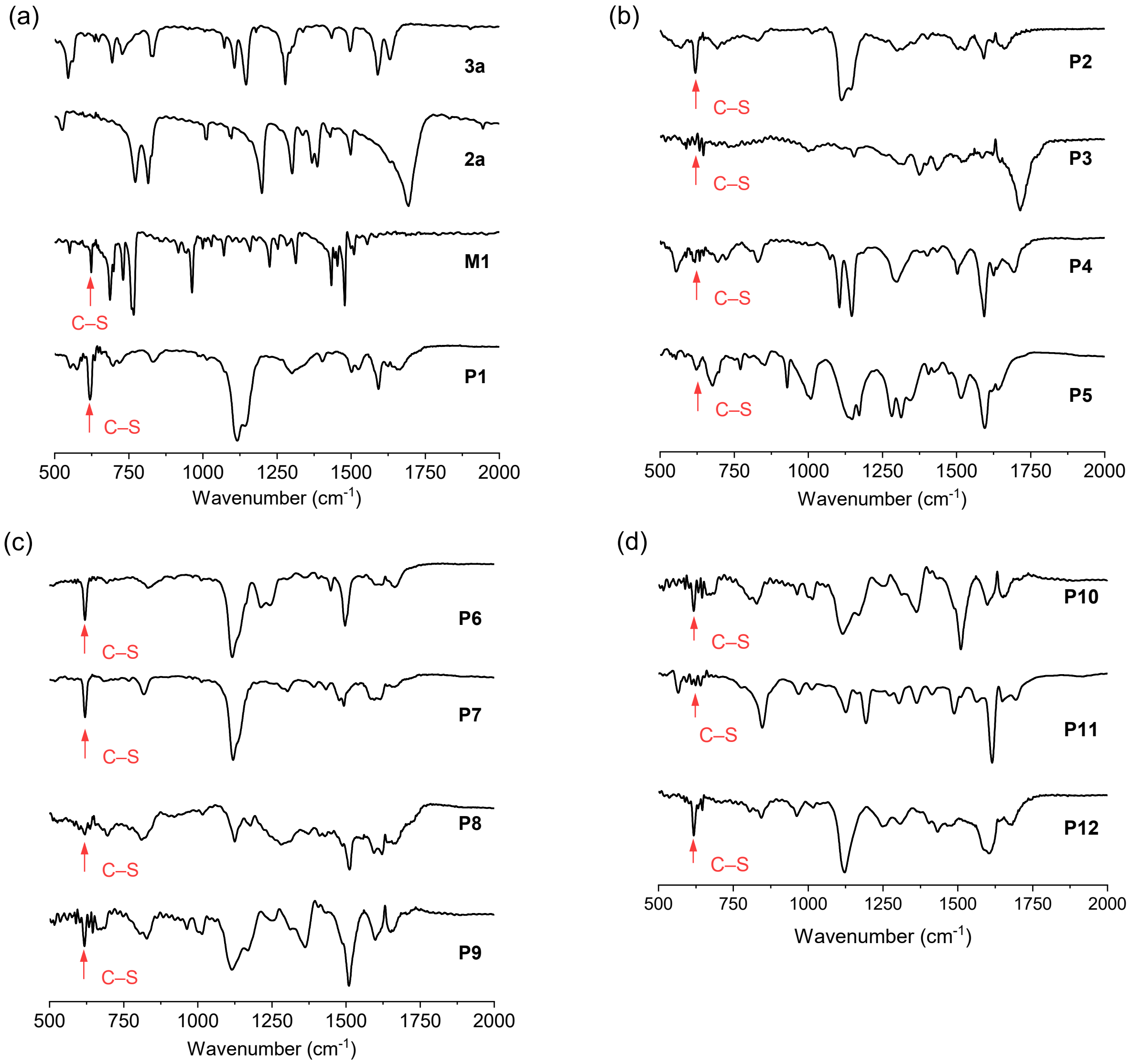

Figure S4. FT-IR spectra of (a) **3a**, **2a**, **M1** and **P1**, (b)–(d) **P2**–**P12**.

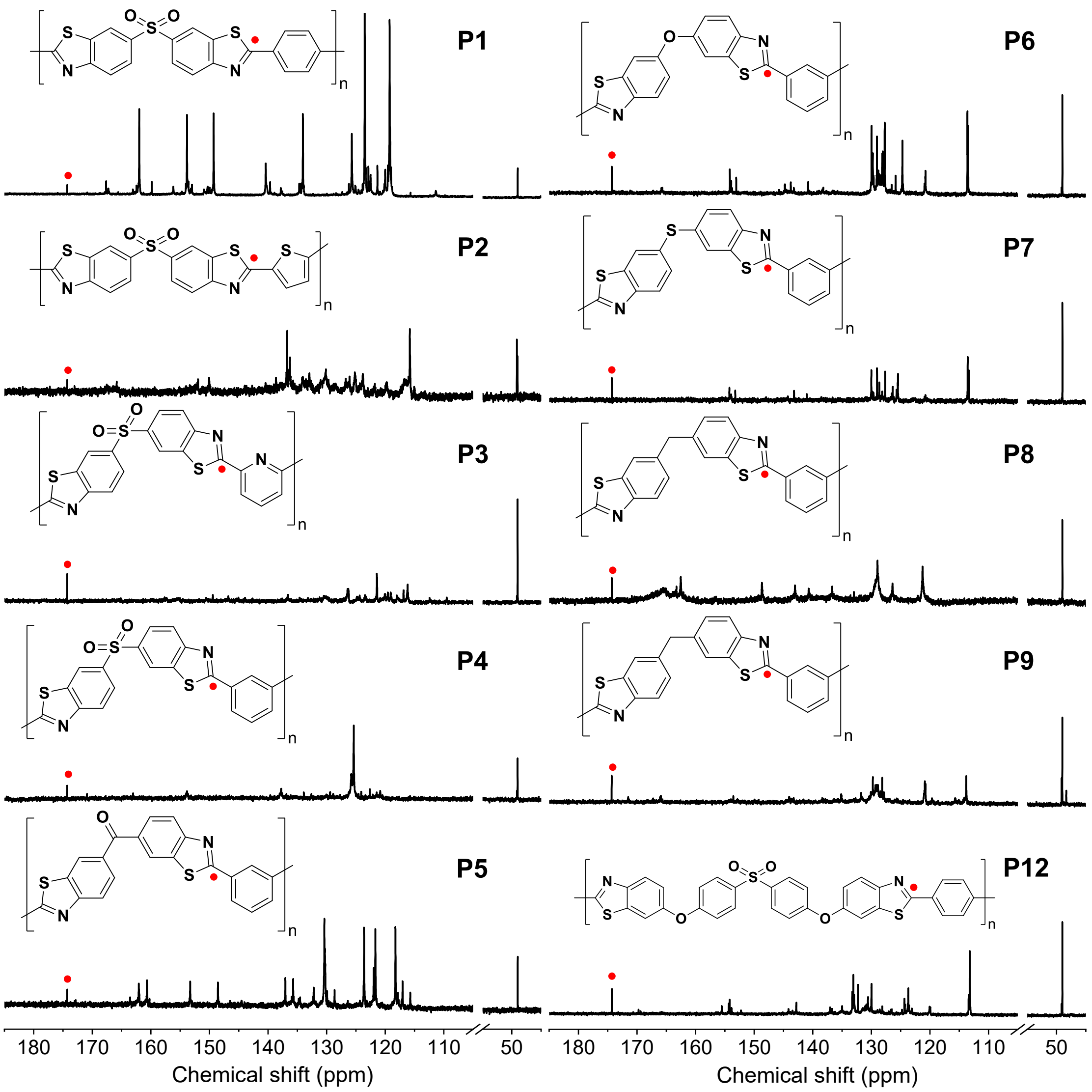

Figure S5. ^{13}C NMR spectra of **P1**−**P9** and **P12** in DMSO-d_6.

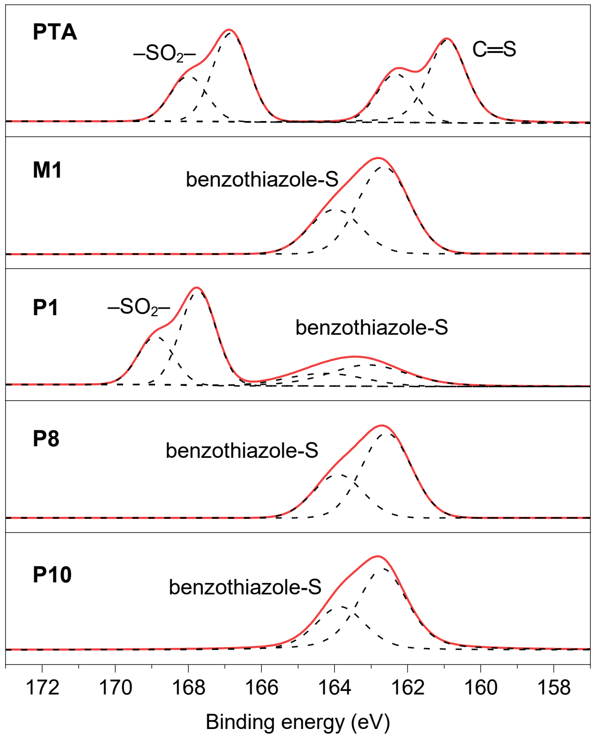

Figure S6. XPS S 2p spectra of polythioamide (PTA), **M1**, **P1**, **P8**, and **P10**.

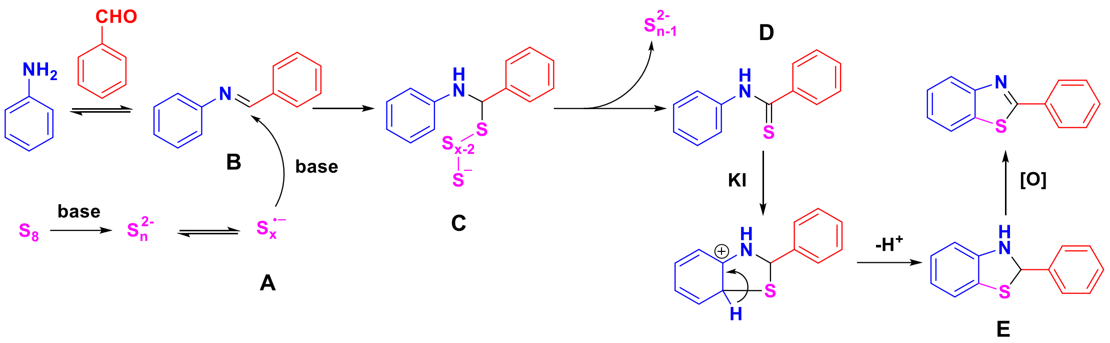

Scheme S1. Proposed mechanism for benzothiazole formation via base-mediated multicomponent polymerization.

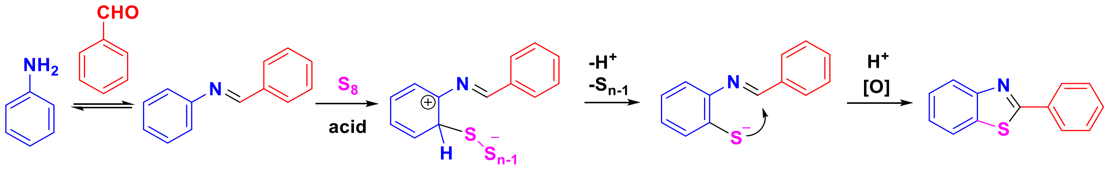

Scheme S2. S_n-mediated electrophilic substitution at aniline *ortho* C–H for benzothiazole formation.

Table S8. Cost estimation of scale-up production of **P1**

Ingredient	Consumption[a]	Price ($/kg)[b]	Cost ($)	Supplier
Sublimed sulfur	0.34 kg	7.43	2.53	Aladdin
Terephthalaldehyde	0.35 kg	33.72	11.81	Aladdin
4,4′-Diaminodiphenyl sulfone	1.30 kg	30.97	40.26	Aladdin
KOH	0.15 kg	9.22	1.39	Aladdin
KI	0.087 kg	189.26	16.46	Sigma-Aldrich
NMP	3.9 L	8.40	32.74	Aladdin
DMSO	1.3 L	13.35	17.36	Aladdin
Total cost: $122.6/kg				

[a] Reference to 1-mol scale synthesis for 1 kg **P1** production. [b] Material prices (analytical grade) were obtained from the market.

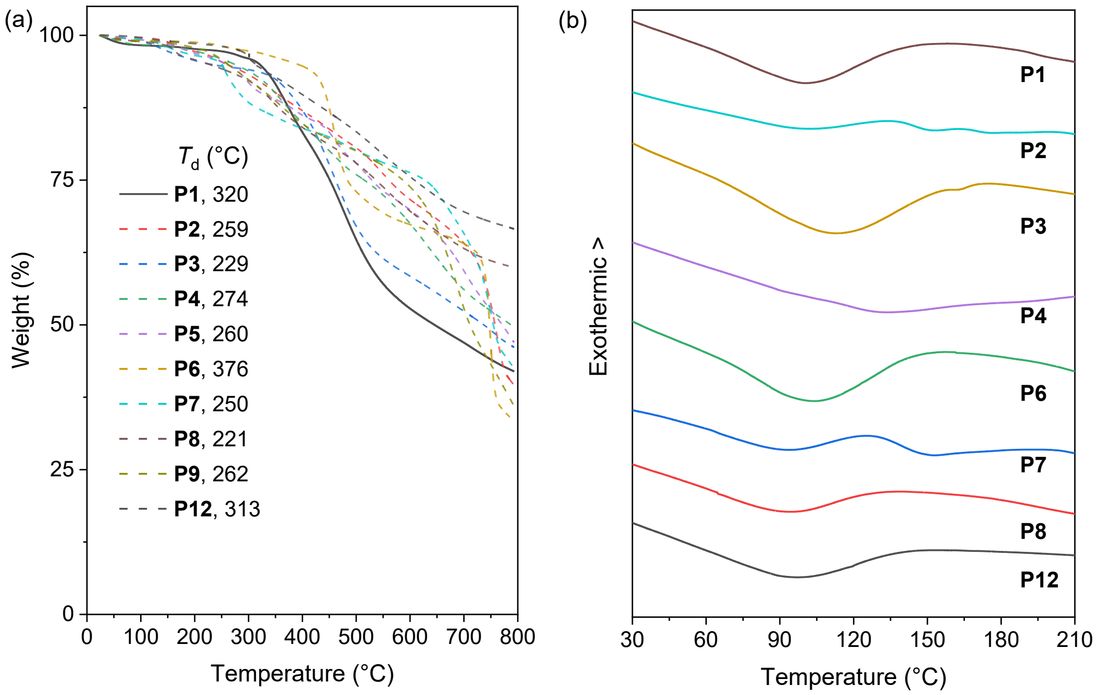

Figure S7. (a) TGA and (b) DSC curves of polybenzothiazoles with a heating rate of 10°C/min under nitrogen.

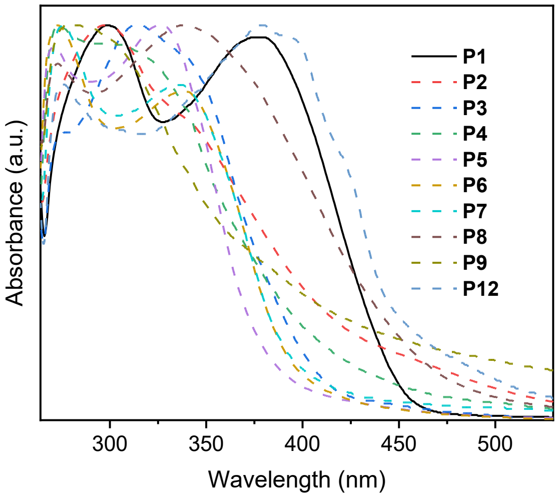

Figure S8. UV–Vis spectra of polybenzothiazoles dissolved in DMF (0.01 g/L).

4. Precious metal extraction performances

4.1 Solubility test of polybenzothiazoles in aqueous solutions

The aqueous solubility of polymer **P1** was systematically investigated under varying thermal and chemical conditions. To evaluate thermal effects, 10 mg of **P1** was dispersed in 10 mL deionized water, heated at 100°C with continuous stirring for 4 h, and then filtered through a 0.22 μm membrane. Chemical stability was evaluated by treating 10 mg of **P1** with 12 M HCl or 12 M NaOH solutions at 25°C for 4 h. UV–Vis analysis of all filtrates (Figure S9a), including controls (untreated **P1**/H_2O suspension at 25°C and 0.01 g/L DMF solution), revealed no detectable dissolution in aqueous media under these extreme conditions.

All synthesized polymers (**P1–P14**) were tested for aqueous solubility by measuring total organic carbon (TOC) of the filtrates (in triplicate, by OI Analytical Aurora 1030W TOC analyzer). After dispersing each polymer in deionized water (1 g/L, 4-h stirring) and filtering through a 0.22 μm membrane, dissolved organic carbon was quantified. TOC values for all samples remained consistently below 0.5 mg/L (Figure S9b), matching ultrapure water background levels and confirming the water-insoluble nature of these polybenzothiazoles.

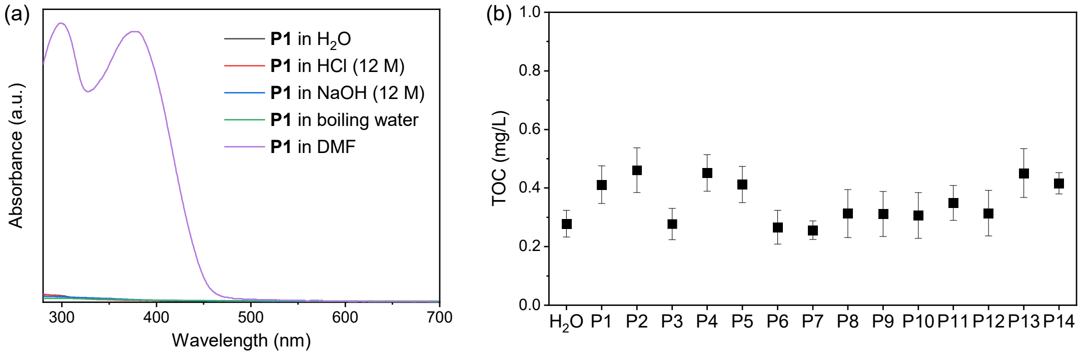

Figure S9. Aqueous solubility assessment. (a) UV–Vis spectra comparisons of **P1**-treated solutions. (b) Total organic carbon (TOC) for **P1–P14** aqueous suspensions.

4.2 Extraction kinetics

Into 100 mL aqueous solutions of $AuCl_3$, $PdCl_2$ and $PtCl_4$ ($[M^{n+}]$ = 100 mg/L, with 2 wt% HNO_3) was added 10 mg of **P1**, **P8**, or **P10**, respectively. After the mixtures were stirred at room temperature for 1 min, 5 min, 10 min, 30 min, 1 h, and 2 h, samples were filtered by a membrane with an aperture of 0.22 μm. The metal concentrations were measured by ICP-OES. The extraction efficiency was calculated to be $\eta = ([M^{n+}]_0 - [M^{n+}])/ [M^{n+}]_0 \times 100\%$, where $[M^{n+}]_0$ (mg/L) is the initial concentration of metal ions, and $[M^{n+}]$ (mg/L) is the remaining concentration of metal ions. The extraction capacity q_t (mg/g) at time t (min) was calculated as $V \times [M^{n+}]_0 \times \eta / m$, where V (L) is the volume of the feeding solution and m (g) is the weight of the polymer.

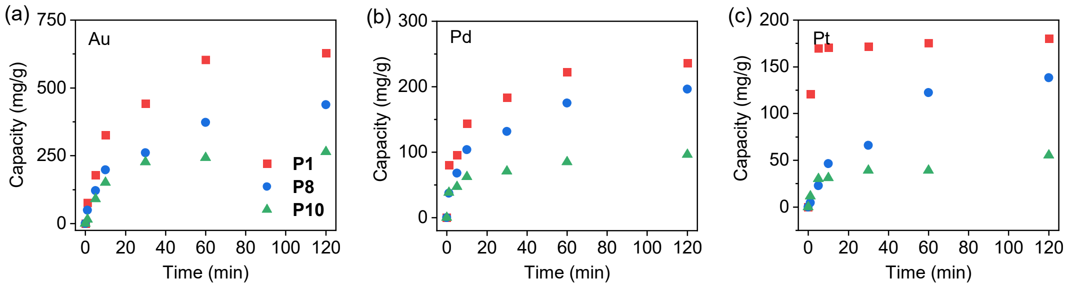

Figure S10. Time-dependent extraction capacity of (a) Au, (b) Pd, and (c) Pt (100 ppm) by **P1**, **P8**, and **P10** (0.1 g/L).

Table S9. Kinetic model fitting parameters of precious metal extraction by **P1**, **P8**, and **P10**

Metal	Polymer	Pseudo-first-order model[a]			Pseudo-second-order model[b]		
		q_e (mg/g)	k_1	R^2	q_e (mg/g)	k_2	R^2
Au	**P1**	605	0.06	0.97	702	1.1×10^{-4}	0.99
	**P8**	378	0.09	0.99	479	1.8×10^{-4}	0.98
	**P10**	268	0.05	0.95	289	3.5×10^{-4}	0.99
Pd	**P1**	216	0.12	0.94	237	7.2×10^{-4}	0.94
	**P8**	178	0.08	0.93	202	9.7×10^{-4}	0.97
	**P10**	82	0.19	0.85	88	7.6×10^{-3}	0.91
Pt	**P1**	174	1.19	0.99	179	1.2×10^{-2}	0.99
	**P8**	146	0.03	0.98	188	2.7×10^{-4}	0.98
	**P10**	55	0.19	0.89	60	2.1×10^{-2}	0.93

[a]Pseudo-first-order model $q_t = q_e\ (1 - \exp(-k_1 \times t))$, where q_e (mg/g) is the equilibrium extraction capacity, k_1 is the model rate constant; [b]Pseudo-second-order model $q_t = (q_e^2 \times k_2 \times t) / (1 + q_e \times k_2 \times t)$, where k_2 is the model rate constant.

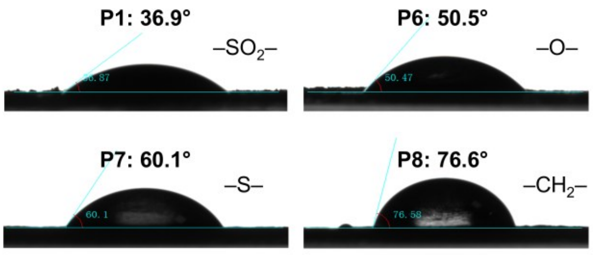

Figure S11. Water contact angles of **P1** (sulfone) and **P6–P8** with different motifs.

4.3 Isotherm studies

Into 100 mL aqueous solutions of $AuCl_3$, $PdCl_2$ and $PtCl_4$ ($[M^{n+}]_0$ = 1−200 mg/L, with 2 wt% HNO_3) was added 10 mg of **P1**. After the mixtures were stirred at room temperature for 2 h, samples were filtered by a membrane with an aperture of 0.22 μm. The metal concentrations were measured by ICP-OES. The extraction data were fitted by Langmuir isotherm model: $q_e = q_m \times K_L \times C_e / (1 + K_L \times C_e)$, where q_e (mg/g) is the equilibrium capacity, q_m (mg/g) is the maximum capacity, K_L (L/mg) is the Langmuir isotherm constant, C_e (mg/L) is the equilibrium concentration; and Freundlich isotherm model: $q_e = K_F \times C_e^{1/n}$, where n is the Freundlich exponent, K_F (mg/g) is the Freundlich isotherm constant.

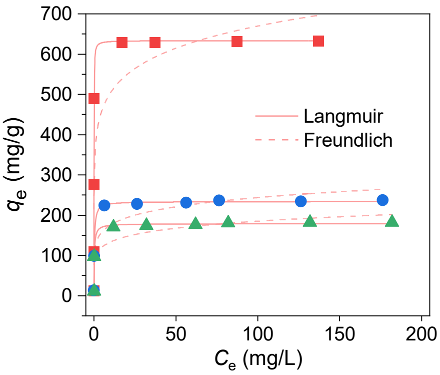

Figure S12. Adsorption isotherms of **P1** for precious metals.

Table S10. Isotherm model fitting parameters of precious metal extraction by **P1**

Precious metals	Langmuir			Freundlich		
	q_m (mg/g)	K_L (L/mg)	R^2	$1/n$	K_F (mg/g)	R^2
Au	634	24.5	0.81	378.9	0.124	0.71
Pd	234	3.9	0.99	126.5	0.142	0.81
Pt	179	4.1	0.98	96.2	0.142	0.81

Table S11. Comparison of maximum extraction capacity (q_m) of **P1** with reported materials

Materials	q_m (mg/g)			References
	Au	Pd	Pt	
Polybenzothiazole **P1**	634	234	179	**This work**
1,4,7,10-tetraazacyclododecane-modified mesoporous silica		162		[1]
2,6-Aminopyridine-grafted 3-formyl-4-hydroxybenzoic acid-modified metal-organic framework (MOF)	403	193		[2]
Cellulose modified with 2-aminothiophenol		163	20	
Cellulose modified with 2-mercaptobenzothiazole		27	5	[3]
Cellulose modified with 2-mercaptopyridine		93	9	
Silica-gel modified with 3-aminopropyl		48		[4]
Amberlite XAD-1180 resin modified with 2-mercaptobenzothiazole		50		[5]
Amberlite XAD-7 resin modified with *p*-diethylphosphonomethylthiacalix[6]arene		19		[6]
Amberlite XAD-7 resin modified with *p*-tert-butylthiacalix[4/6]arenes		60		[7]
Amine-rich polymeric capsule	576			[8]
Ammonium-functionalized Zr-based MOF		119	243	[9]
Bismuth carbonate supported over carbon black	13			[10]
Boron-nitrogen co-doped walnut shell biochar	461	134	47	[11]
Chitosan modified with dibenzo-18-crown-6-ether		19	23	[12]
Chitosan modified with ethylenediamine		138	171	[13]
Cyano-activated cobalt hexacyanoferrate			25	[14]
Dialdehyde carboxymethyl cellulose		89	1	[15]
Dimethylamine-modified lignophenol		66	121	[16]
Dimethylaniline-modified paper	906	224	176	[17]
Fe_3O_4 nanoparticle		11	13	[18]
Glycine-modified crosslinked chitosan resin	170	120	122	[19]
Graphene oxide	108	81	71	[20]
Graphene oxide-tetraoctylammonium bromide		93		[21]
Graphene oxide/calcium alginate hydrogel	82			[22]
Reductive hydrazide-functionalized polymer	2847	1078	714	[23]
Leaf biomass *T.* catappa L.		42	23	[24]
Macrocyclic polyether-modified tetraethoxysilane		34		[25]
Magnetic alginate polymer-imprinted diatomite composite		60		[26]

Magnetic nanoparticle modified with third-generation dendrimer	4	3		[27]
Amberlite XAD-7 modified with 1,3-bis(2-(octylthio)propan-2-yl)benzene		44		[28]
Mesoporous carbon	492	64	78	[29]
MIL-101(Cr)-NH_2		278	141	[30]
Modified $CoFe_2O_4$	120			[31]
Modified polyacrylonitrile		15	10	[32]
Nanometer-sized titanium dioxide	23	12		[33]
Pd^{2+} ion-imprinted Cr-based MOF		193		[34]
Polyetherimide-incorporated algal bead		136	115	[35]
Plant tannin-immobilized Fe_3O_4@SiO_2 microsphere	917	196		[36]
Polyacryloyl hydrazide-brushed CNT membrane	292	187	267	[37]
Polyamine chelating resin			162	[38]
Polyamine modified beads/nanofibers		31	50	[39]
Polysaccharide-derived mesoporous carbon		156	246	[40]
SiO_2 modified with 2,6-bis(5,6-dialkyl-1,2,4-triazin-3-yl)pyridine		75		[41]
SiO_2 modified with 2-vinylpyridine and ethylene glycol dimethacrylate		38		[42]
SiO_2 modified with bis-(4,7,7-trimethyl-3-oxobicyclo[2.2.1]heptan-2-ylidene)-1,10-phenanthroline-2,9-bis(carbohydrazonamide)		35		[43]
Sulfur modified crown ether		178	66	[44]
Tannic acid-coated porous microsphere	52			[45]
Tannin with tetraethylenepentamine		187	289	[46]
Thiol-modified mesoporous silica		10	18	[47]
Thiourea-modified chitosan microsphere		42	57	[48]
Thiourea-modified Zr-based MOF	326			[49]
Zr(IV)-based MOF	280	120	166	[50]
Zr-based MOF modified with 2,6-bis(allylsulfanyl) terephthalic acid		45		[51]
Zr-based MOF modified by 2-aminoterephthalic acid		168		[52]

4.4 Metal selectivity

Selectivity test with mixed metal ions. Into 10 mL aqueous solutions of NaCl, $MgCl_2$, $AlCl_3$, KCl, $Ca(NO_3)_2{\cdot}4H_2O$, $CrCl_3{\cdot}6H_2O$, $MnCl_2$, $FeCl_3$, $NiCl_2{\cdot}6H_2O$, $CoCl_2$, $CuCl_2$, $ZnCl_2$, $CdCl_2$, $SnCl_4$, $SbCl_3$, $CeCl_3{\cdot}7H_2O$, $Pb(NO_3)_2$, $AuCl_3$, $PdCl_2$, and $PtCl_4$ ($[M^{n+}]_0$ = 10 ppm or 0.1 ppm, with 5 wt% HCl and trace HNO_3) was added 10 mg of **P1**. After the mixtures were stirred at room temperature for 1 h, samples were filtered by a membrane with an aperture of 0.22 μm. The metal concentrations were measured by ICP-OES.

Selectivity test with single metal ion. Into 10 mL aqueous solutions of $CuCl_2$, $CoCl_2$, $NiCl_2{\cdot}6H_2O$, $CrCl_3{\cdot}6H_2O$, $FeCl_3$, $AuCl_3$, $PdCl_2$, and $PtCl_4$ ($[M^{n+}]_0$ = 100 ppm, with 5 wt% HCl) was added 20 mg of **P1**, respectively. After the mixtures were stirred at room temperature for 1 h, samples were filtered by a membrane with an aperture of 0.22 μm. The metal concentrations were measured by ICP-OES.

Ultra-trace metal extraction from spiked surface water. Natural surface water was collected from the Yangtze River, Shanghai, China, which was then spiked with 10 ppm of Cu^{2+} ($CuCl_2$), Fe^{3+} ($FeCl_3$) and Ni^{2+} ($NiCl_2{\cdot}6H_2O$) and 1 ppb of Au^{3+} ($AuCl_3$), Pd^{2+} ($PdCl_2$), and Pt^{4+} ($PtCl_4$). **P1** (1 g/L) was added to the spiked water, which was stirred at 500 rpm for 1 h. Water samples were collected after filtration through 0.22 μm filter membranes and then subjected to measurements using ICP-MS.

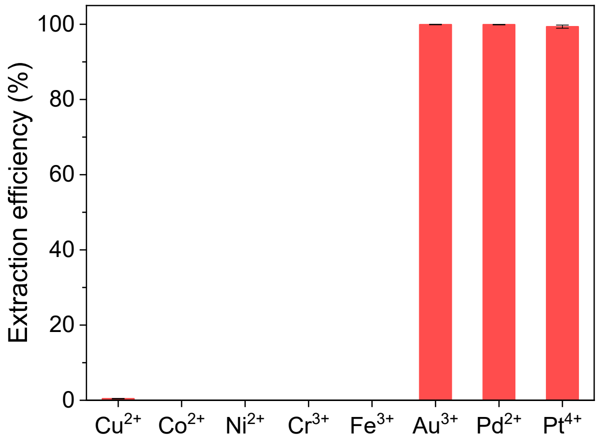

Figure S13. Selective extraction of precious metals in single metal ion solutions (100 ppm) using **P1** (2 g/L).

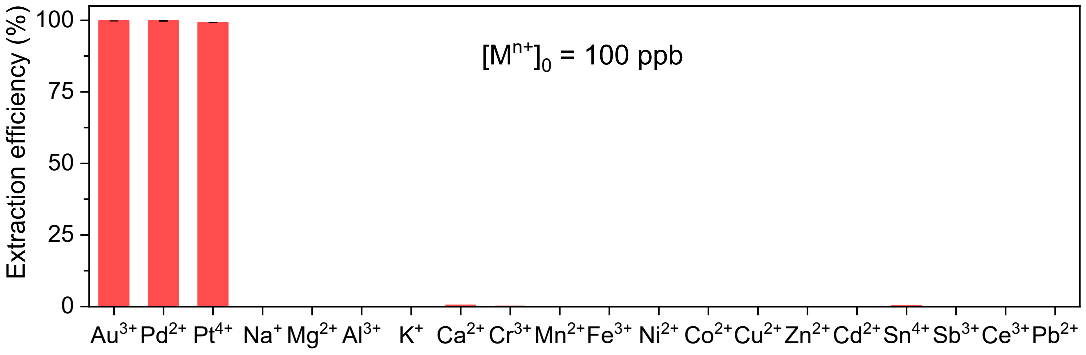

Figure S14. Selective extraction of trace precious metals by **P1** (1g/L) in an aqueous solution with mixed metal ions.

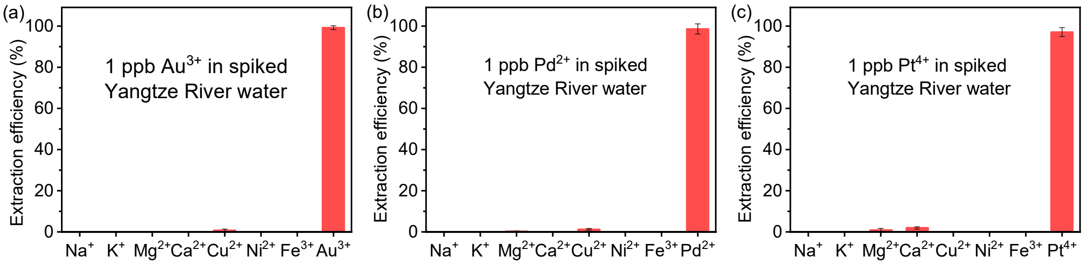

Figure S15. Selective extraction of ultra-trace (1 ppb) precious metals by **P1** (1 g/L) in spiked Yangtze River water with 10 ppm of Cu^{2+}, Fe^{3+} and Ni^{2+}.

4.5 Influence of pH and zeta potential analysis

Aqueous solutions with different pH values of 0.1–9.0 were prepared by mixing HCl solution and NaOH solution with different proportions. Into 10 mL of each solution, 10 μL aqueous solutions of $AuCl_3$, $PdCl_2$, and $PtCl_4$ was added, respectively ($[M^{n+}]$ = 10 ppm). 10 mg of **P1** was then added to each solution and the mixtures were stirred at room temperature for 1 h. Water samples were filtered by a membrane with an aperture of 0.22 μm, and the metal concentrations were measured by ICP-OES.

Zeta potentials of **P1** powder at varying pH were analyzed by a nanoparticle size and zeta potential analyzer (DLS, Malvern Zetasizer Nano ZS90).

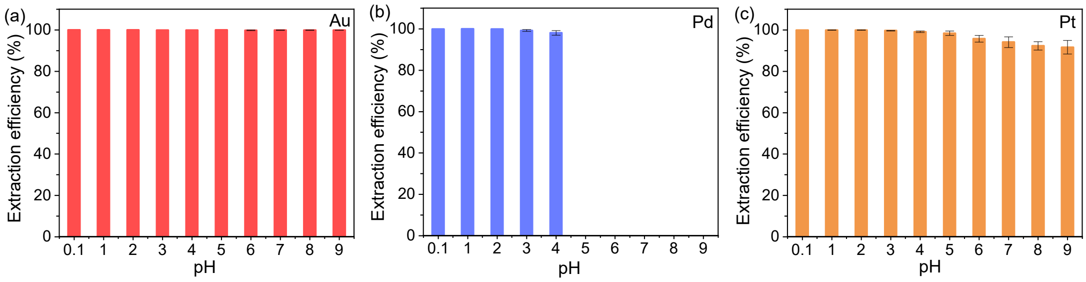

Figure S16. Extraction of (a) Au^{3+}, (b) Pd^{2+}, and (c) Pt^{4+} (10 ppm) by **P1** (1 g/L) at varying pH values.

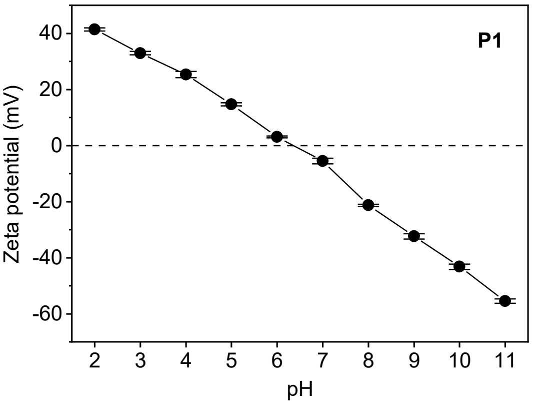

Figure S17. Zeta potentials of P1 in water at pH 2–11.

4.6 Membrane fabrication and evaluation

Fabrication. P1 (4.0 g, M_w = 37.6 kDa), polyethersulfone (PES, 16.0 g, M_w = 58.0 kDa), and polyvinylpyrrolidone (PVP, 4.0 g, M_w = 10.0–16.0 kDa) were dissolved in DMAc to prepare a casting mixture totaling 100.0 g. The mixture was magnetically stirred for 12 h and then deaerated statically for 24 h. The solution was scraped onto a clean glass plate using a scraper knife to form an even film. This film was immediately immersed in pure water for a phase inversion process for 30 min, forming a yellow membrane, which was then carefully peeled off. The membrane was rinsed several times with pure water and soaked in water for at least 24 h before use. The membrane was then cut into a proper size (Φ = 2.5 cm) and compacted with water at a pressure of 1 bar for 1 h.

Characterization. The surface and cross-sectional morphologies of the porous membrane were observed by SEM. The pore size distribution and porosity were analyzed by mercury intrusion porosimetry (MIP) using a Micromeritics AutoPore V 9620 analyzer, with mercury intrusion pressures ranging from 0.1 to 61,000 psi and a contact angle of 130°.

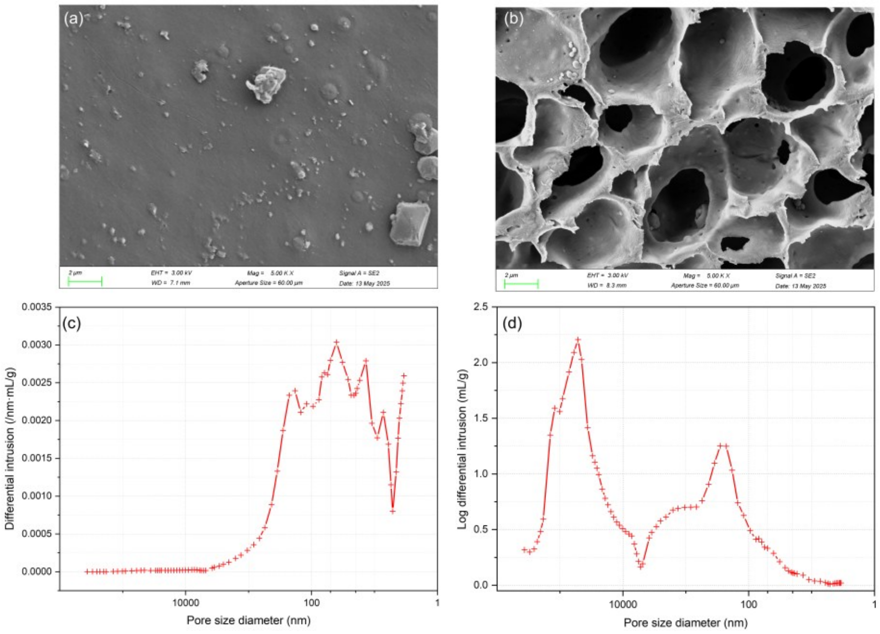

Figure S18. Membrane characterization. SEM of (a) surface and (b) cross-section of **P1**-derived porous membrane. (c) Pore size distribution by mercury intrusion porosimetry (MIP). (d) Dominant pore region analysis by MIP.

Filtration experiments. Filtration experiments were conducted in a single-pass dead-end filtration setup with varied feed concentrations (0.1, 1, and 10 ppm), transmembrane pressures (0.3, 0.5, and 1 bar) and durations (100–600 min). The flux F (L m^{-2} h^{-1} bar^{-1}) is calculated to be $F = \Delta V / (A \times \Delta t \times P)$, where ΔV (L) is the volume of permeated water, A (m^2) is the effective membrane area, Δt (h) is the filtration time, and P (bar) is the operational transmembrane pressure. The metal concentrations in the permeate were measured by ICP-OES.

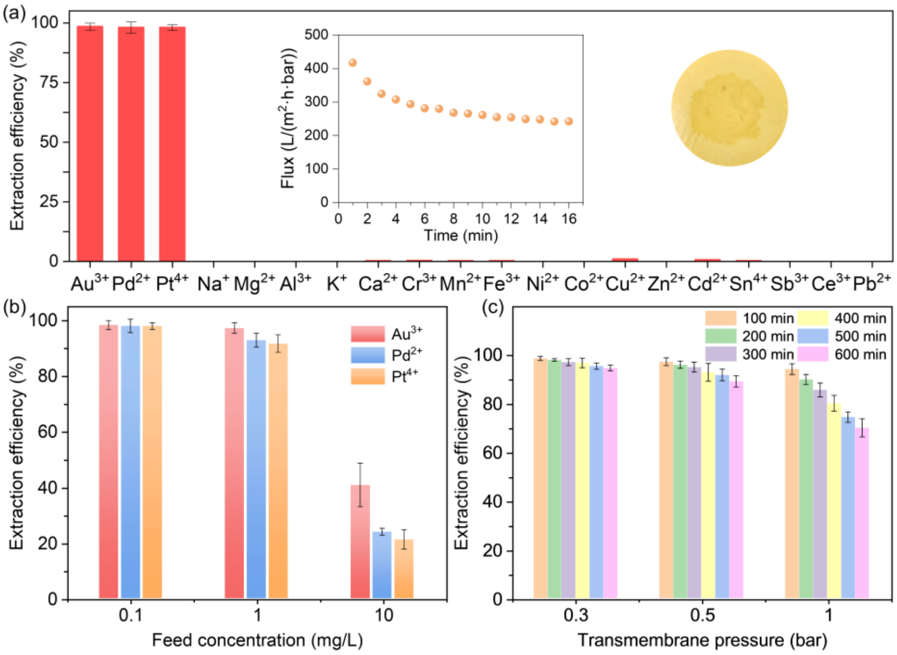

Figure S19. Membrane performance evaluation. (a) Selective extraction of trace precious metals (100 ppb) using the **P1**-based membrane in a single-pass dead-end filtration setup (transmembrane pressure at 0.5 bar, flux at 240 L m^{-2} h^{-1} bar^{-1}). (b) Extraction efficiency of precious metals under different feed concentrations in a single-pass filtration (transmembrane pressure at 0.5 bar). (c) Time-dependent extraction efficiency of Au^{3+} (1 ppm) under different transmembrane pressures of 0.3, 0.5, and 1 bar.

5. Extraction mechanism

5.1 Synthesis of M1-metal complexes

M1 (211 mg, 1.0 mmol) was dissolved in 16 mL of methanol and $AuCl_3$ (304 mg, 1 mmol), $PdCl_2$ (177 mg, 1 mmol), and $PtCl_4$ (337 mg, 1 mmol) were dissolved in 4 mL of H_2O, respectively. The aqueous solutions were added dropwise to the methanol solution and stirred at room temperature for 1 h. After the reaction, the precipitates were removed by centrifugation. The solvents from the supernatants were then removed under reduced pressure to obtain solids of **M1**-metal complexes.

M1-Au. ^{1}H NMR (600 MHz, DMSO-d_6), δ (TMS, ppm): 9.85 (s, 1H), 8.16 (d, J = 7.9 Hz, 1H), 8.14 – 8.08 (m, 3H), 7.62 – 7.54 (m, 5H), 7.49 (t, J = 7.3 Hz, 1H). ^{13}C NMR (150 MHz, DMSO-d_6), δ (TMS, ppm): 167.82, 154.02, 134.95, 133.34, 131.92, 129.89, 127.71, 127.17, 126.05, 123.38, 122.84.

M1-Pd. ^{1}H NMR (600 MHz, DMSO-d_6), δ (TMS, ppm): 10.26 (s, 1H), 7.99 – 7.94 (m, 2H), 7.82 – 7.77 (m, 3H), 7.62 – 7.23 (m, 6H), 7.10 (t, J = 7.4 Hz, 1H). ^{13}C NMR (150 MHz, DMSO-d_6), δ (TMS, ppm): 167.96, 153.87, 134.86, 133.19, 131.95, 129.89, 127.66, 127.20, 126.09, 123.27, 122.78.

M1-Pt. ^{1}H NMR (600 MHz, DMSO-d_6), δ (TMS, ppm): 10.25 (s, 1H), 7.99 – 7.94 (m, 3H), 7.81 – 7.76 (m, 2H), 7.59 (dd, J = 5.7, 1.9 Hz, 3H), 7.55 – 7.49 (m, 3H), 7.36 (dd, J = 8.5, 7.3 Hz, 2H). ^{13}C NMR (150 MHz, DMSO-d_6), δ (TMS, ppm): 167.99, 153.83, 134.83, 133.17, 132.03, 129.98, 127.69, 127.28, 126.16, 123.27, 122.89.

5.2 DFT calculation methods

DFT theoretical calculations were carried out using the Gaussian 16 program package.[53] The PBE0 density functional method with the D3BJ dispersion correction was employed to perform the computations.[54-55] The def2-SVP basis set was used for the atoms in geometry optimizations using the polarizable continuum model (PCM) with water as the solvent.[56-57] Vibrational frequency analyses at the same level of theory were performed to characterize stationary points as local minima without any imaginary frequencies. Single-point energy calculations were carried out using the def2-TZVP basis set to provide better energy correction.[58] **M1** molecule was used as the model unit to calculate the binding energies of metal species including Au^{3+}, Pd^{2+}, Pt^{4+}, Au^0, and Pt^{2+} on different potential sites as $\Delta E = E_{\text{configuration}} - E_{\text{M1}} - E_{\text{metal}}$, where $E_{\text{configuration}}$, E_{M1}, and E_{metal} (eV) represent the energy of **M1** bonded with metals, **M1**, and metal species in optimized geometries, respectively.

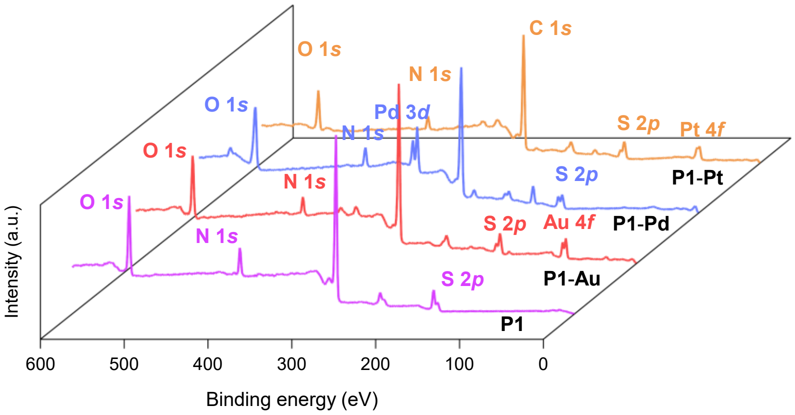

Figure S20. XPS survey of **P1**, **P1**-Au, **P1**-Pd, and **P1**-Pt.

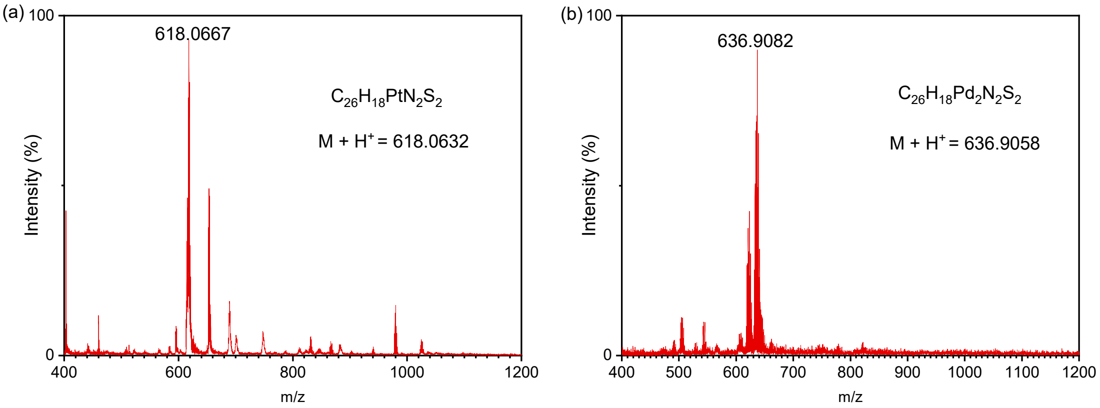

Figure S21. MALDI-TOF MS spectra of (a) **M1**-Pt and (b) **M1**-Pd.

6. Stability and practical metal recovery

Metal elution and regeneration of P1. After metal extraction, the **P1** solids were collected and added to 20 mL of 0.05 M thiourea (with 5 wt% HCl) for metal elution. The mixture was ultrasonicated at room temperature for 4 h, after which the suspension was centrifuged at 10,000 rpm for 10 min. The precipitate was filtered and washed with pure water. The regenerated solids were then used in subsequent phases of extraction process.

Precious metal recovery from precious metal-bearing wastes. Precious metal bearing wastes including discarded CPUs, spent Pd-containing three-way catalysts, and spent Pt/Al_2O_3 catalysts were immersed in aqua regia for 4 h at room temperature, respectively. The solid surface was then rinsed properly and the leachates were filtered to remove any insoluble residues. 10 mg of **P1** was added to 20 mL of the leachates. The mixtures were stirred at room temperature for 30 min. Water samples were filtered using a 0.22 μm membrane, and the metal concentrations were measured by ICP-OES before and after extraction.

Pyrolysis. The **P1**-metal complexes were pyrolyzed in air at 600°C for 3 h, followed by 3 h at 1,000°C with a heating rate of 10°C/min. The **P1**-Au complex afforded a golden product with an approximate gold content of 91.2 wt%, as measured by SEM-EDS. The **P1**-Pd complex afforded a black product with an approximate palladium content of 90.2 wt%, and the **P1**-Pt complex afforded a black product with an approximate platinum content of 94.4 wt%.

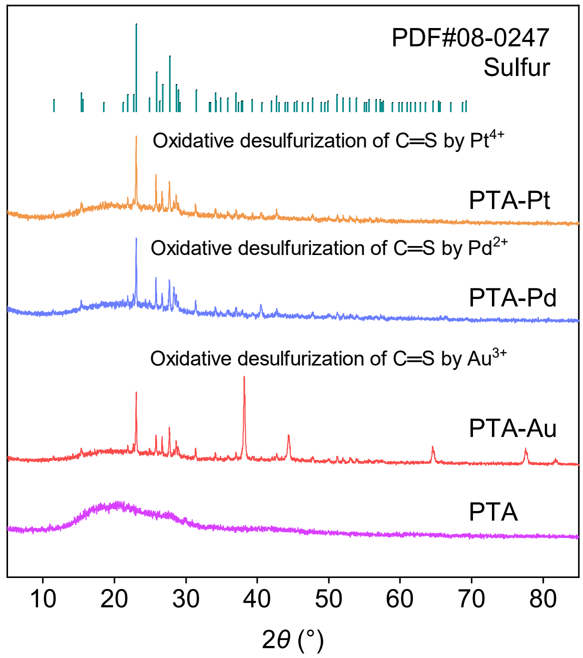

Figure S22. Oxidative desulfurization of a less stable polythioamide [NH–C(=S)] (structural analogue to **P1**) after treatment with aqueous solutions containing 3 g/L of Au^{3+}, Pd^{2+}, and Pt^{4+}, respectively.

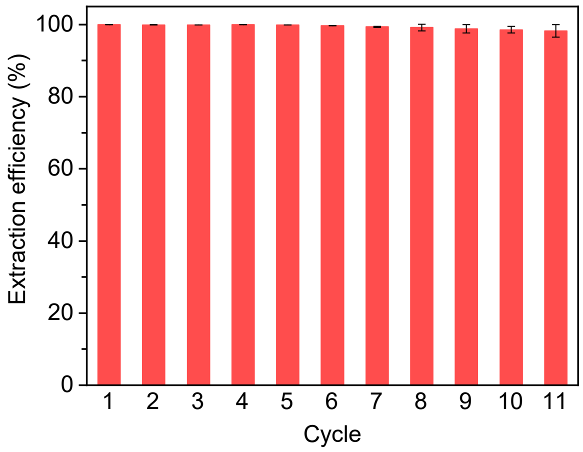

Figure S23. Extraction-elution cyclability of **P1** in Au^{3+} aqueous solution (100 ppm), using an acidic thiourea elution solution containing 0.05 M thiourea with 5 wt% HCl.

Table S12. Distribution coefficients (K_d) of **P1** for metals in real waste leachates

	Coexisting metals				
CPU	Cu^{2+}	Fe^{3+}	Ni^{2+}	Zn^{2+}	Au^{3+}
K_d (L/g)[a]	0.015	0.024	0.017	0.007	1,167.6
Three-way catalyst	Al^{3+}	Ca^{2+}	Mg^{2+}	Pd^{2+}	
K_d (L/g)	0.010	0.012	0.009	807.7	
Pt/Al_2O_3	Al^{3+}	Pt^{4+}			
K_d (L/g)	0.014	690.0			

[a] the distribution coefficient is calculated to be $K_d = (C_0 - C_e)/C_e \times (V/m)$, where C_0 and C_e (mg/L) are the initial and equilibrium metal concentrations, V (L) is the solution volume, and m (g) is the mass of the polymer. The selectivity coefficient between two metal ions, M_1/ M_2, is expressed as the ratio of their respective K_d values.

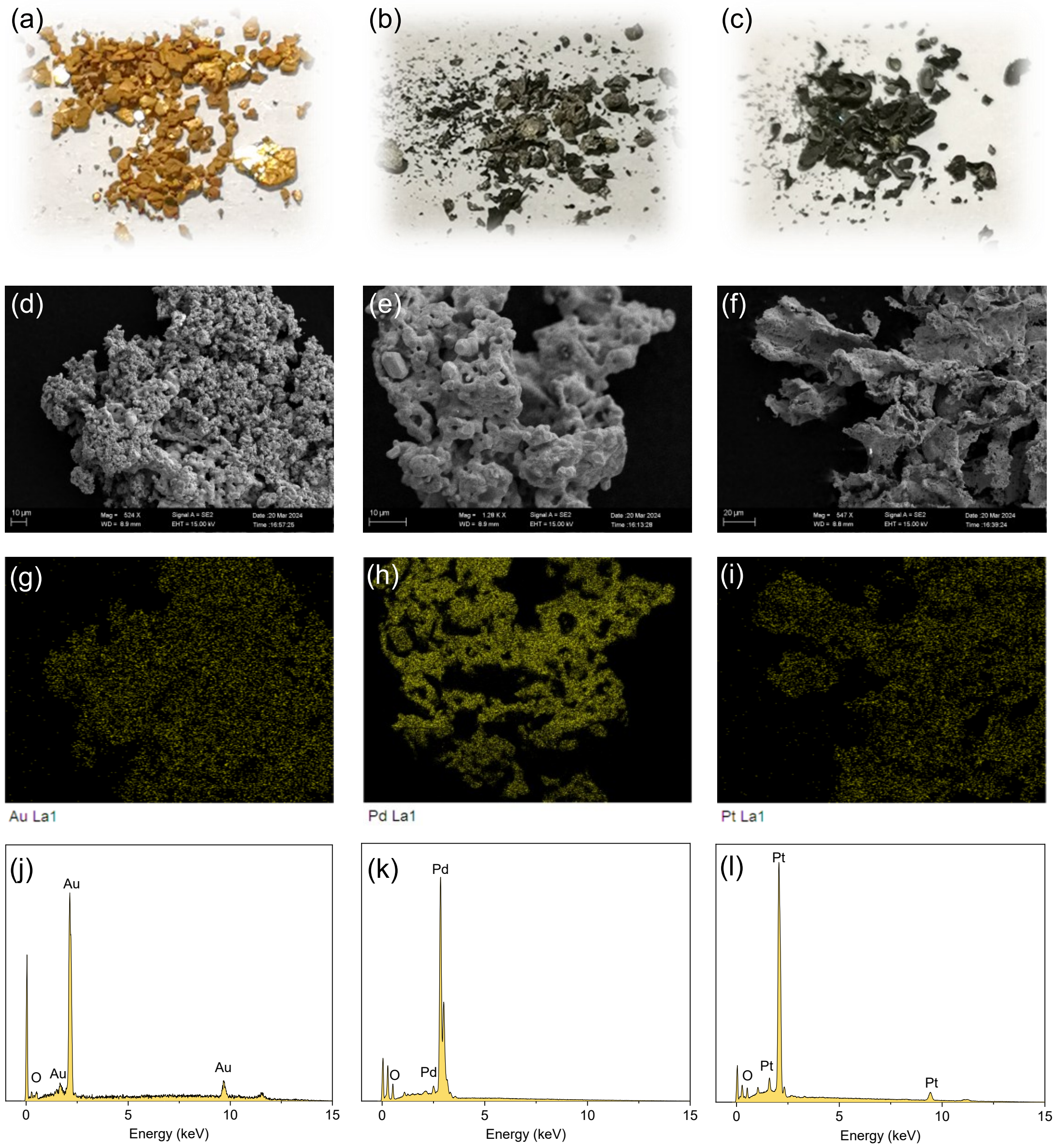

Figure S24. Pyrolyzed solids of (a) **P1**-Au, (b) **P1**-Pd, and (c) **P1**-Pt. (d–l) Corresponding SEM-EDS analysis.

7. Original ^{1}H and ^{13}C NMR spectra

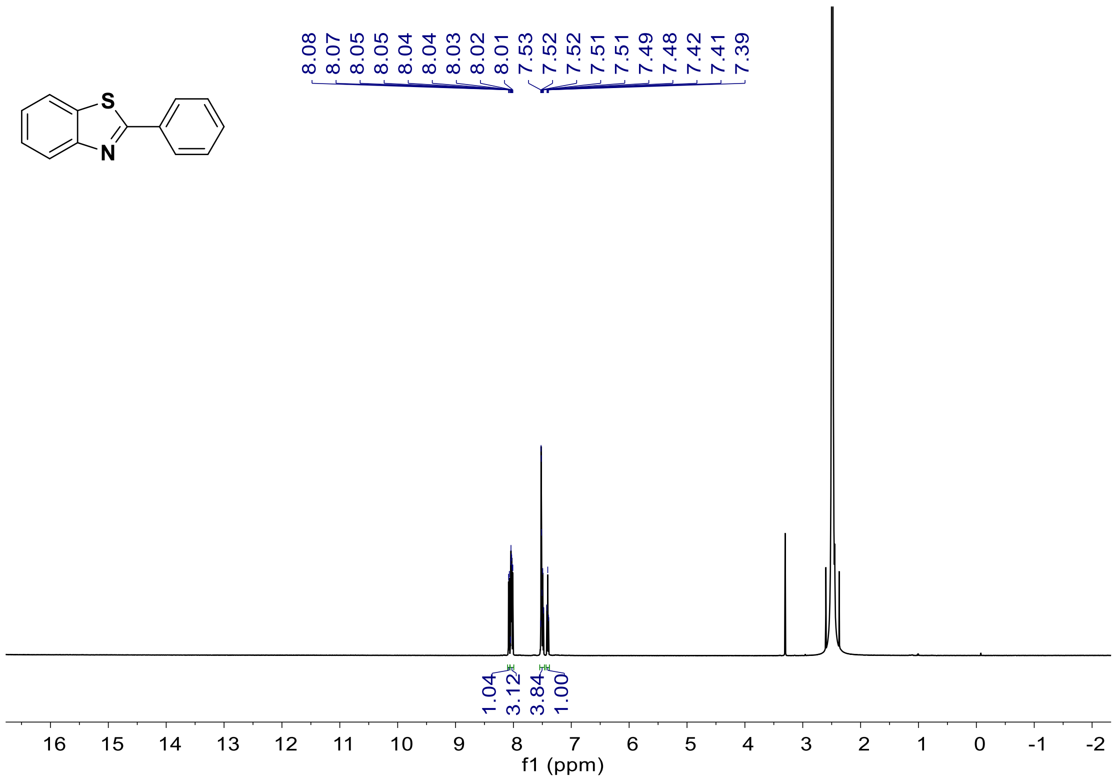

Figure S25. ^{1}H NMR spectrum of **M1** in DMSO-d_6.

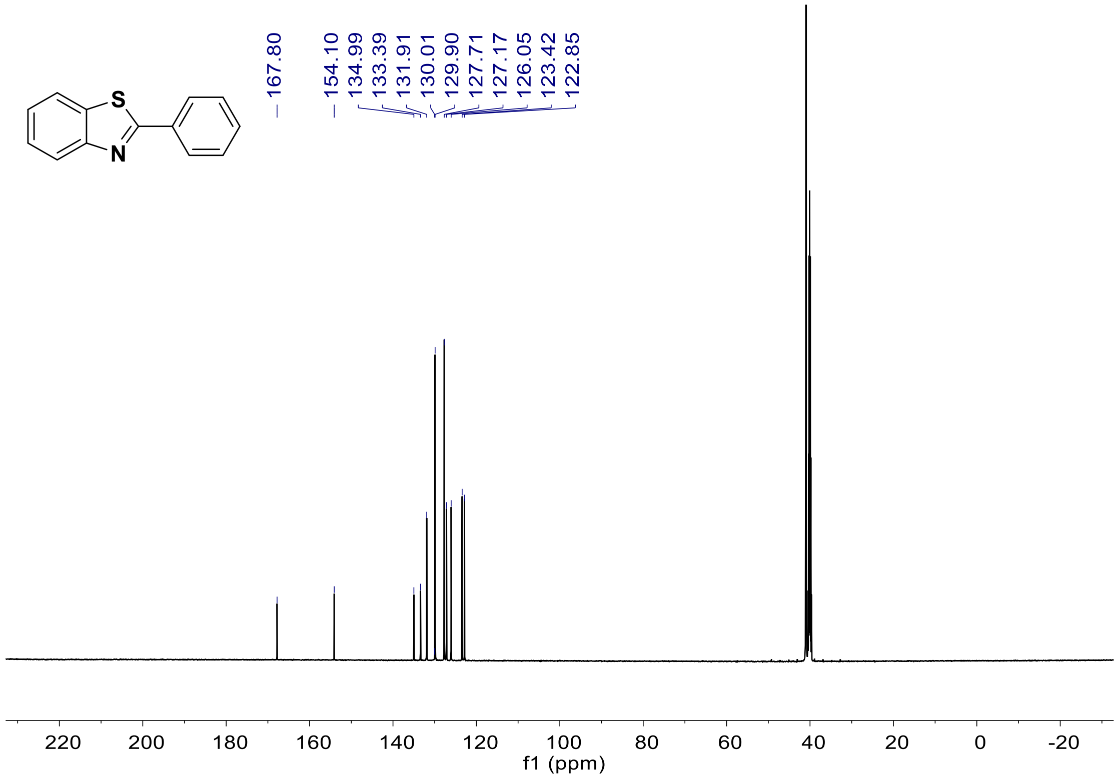

Figure S26. ^{13}C NMR spectrum of **M1** in DMSO-d_6.

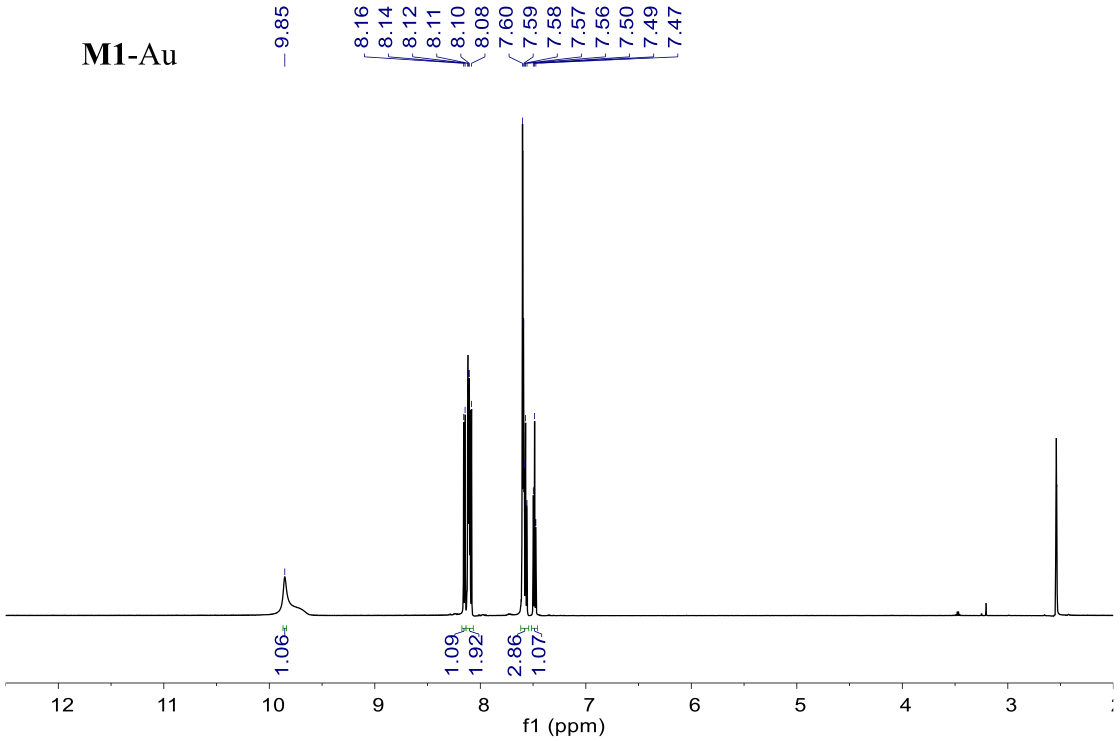

Figure S27. ^{1}H NMR spectrum of **M1**-Au in DMSO-d_6.

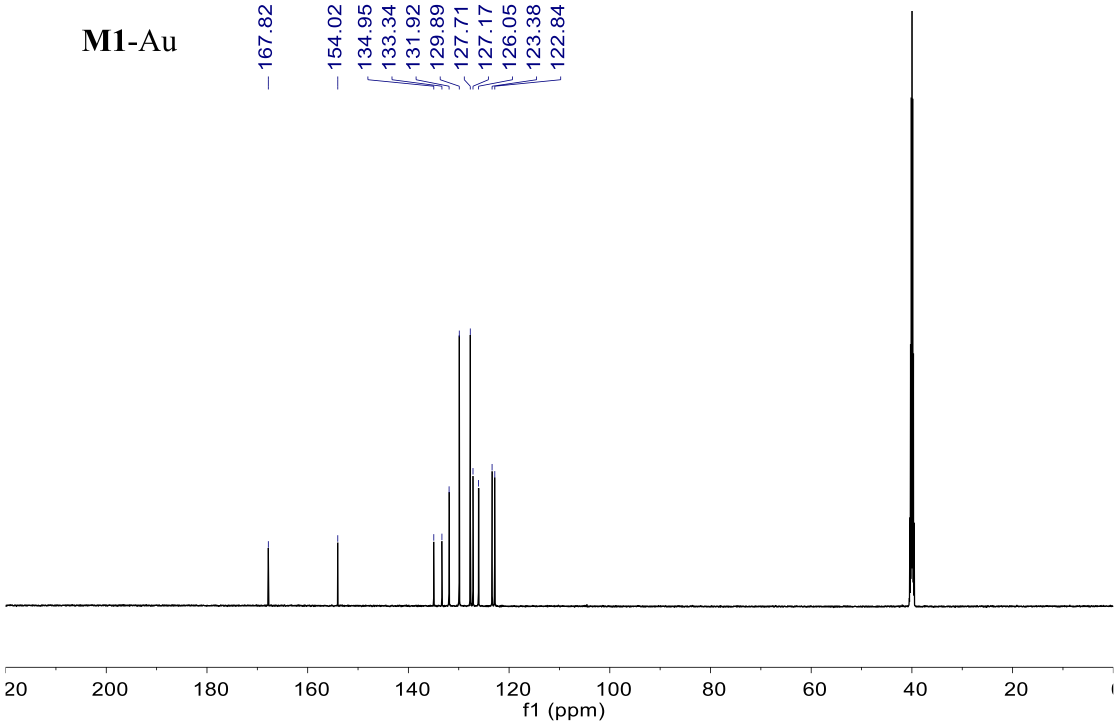

Figure S28. ^{13}C NMR spectrum of **M1**-Au in DMSO-d_6.

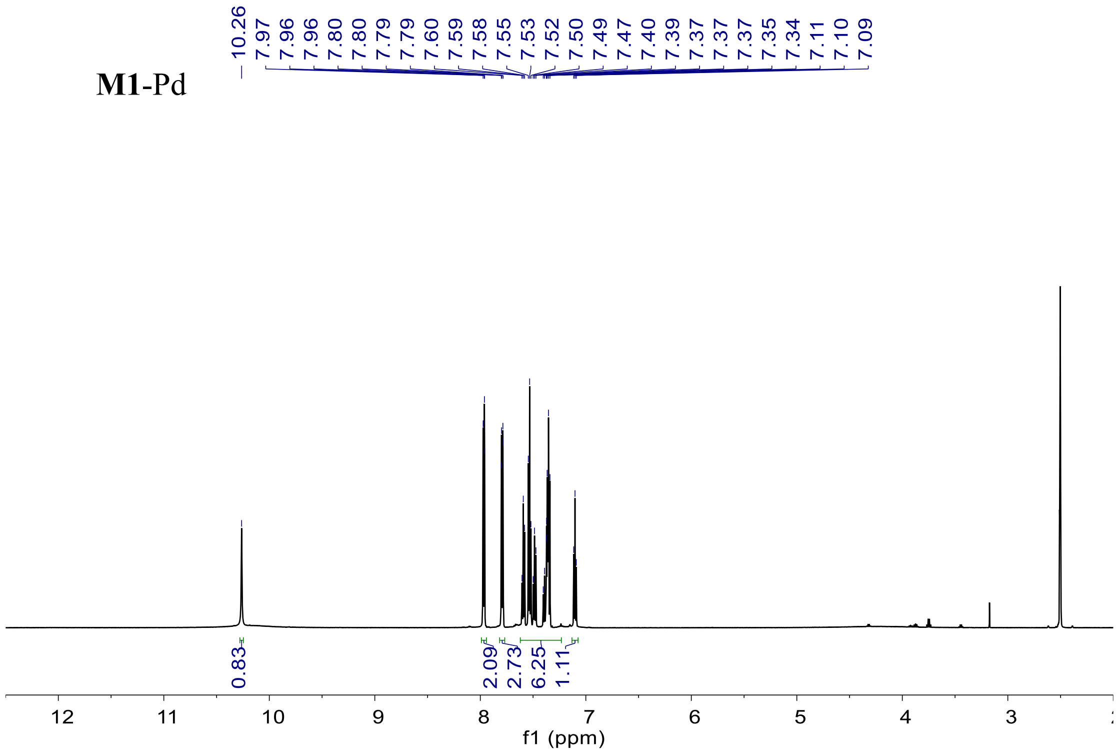

Figure S29. ^{1}H NMR spectrum of **M1**-Pd in DMSO-d_6.

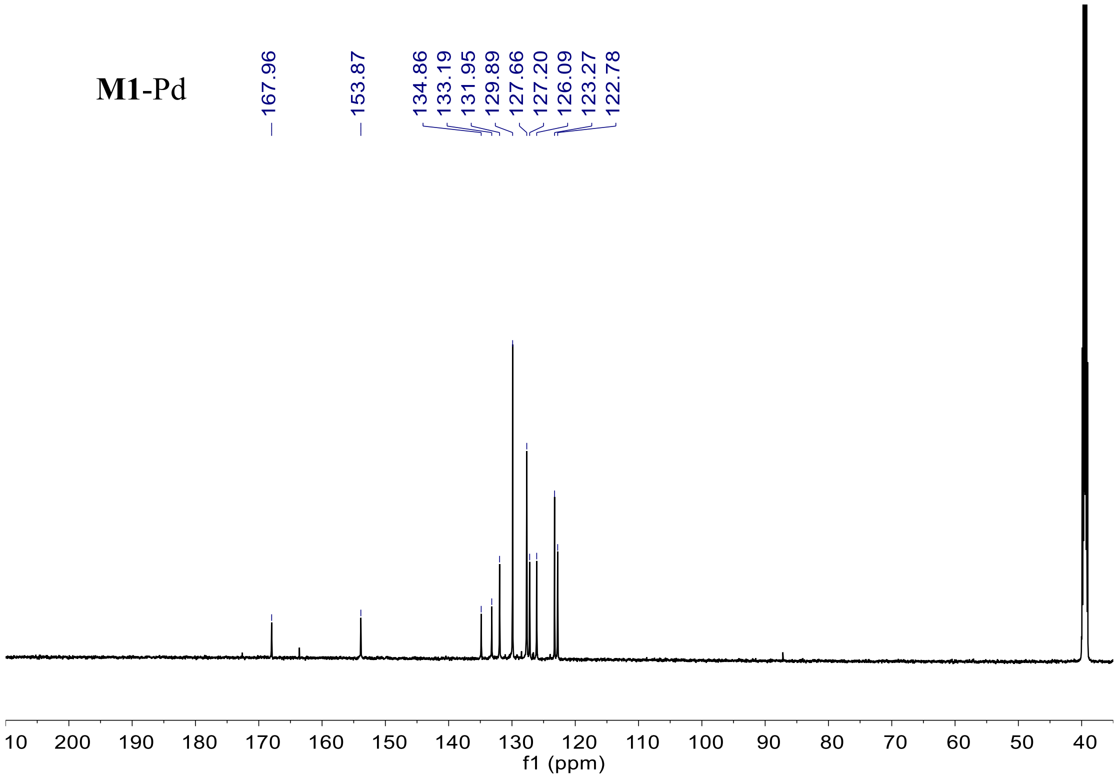

Figure S30. ^{13}C NMR spectrum of **M1**-Pd in DMSO-d_6.

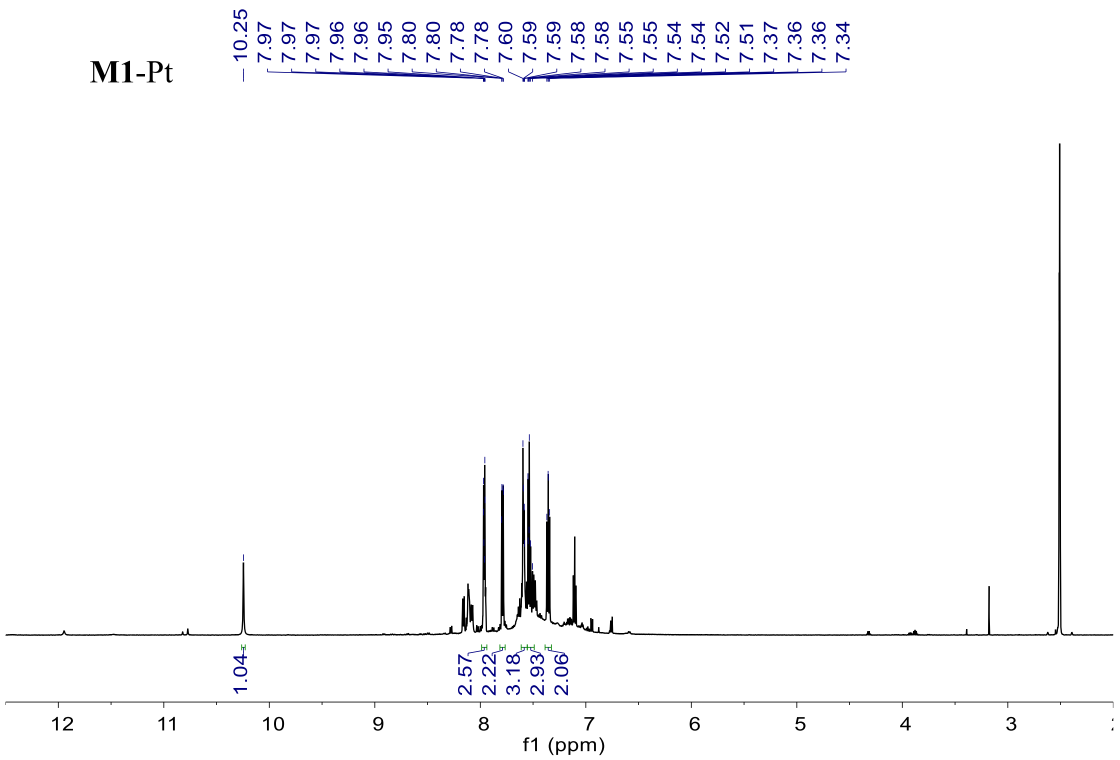

Figure S31. ^{1}H NMR spectrum of **M1**-Pt in DMSO-d_6.

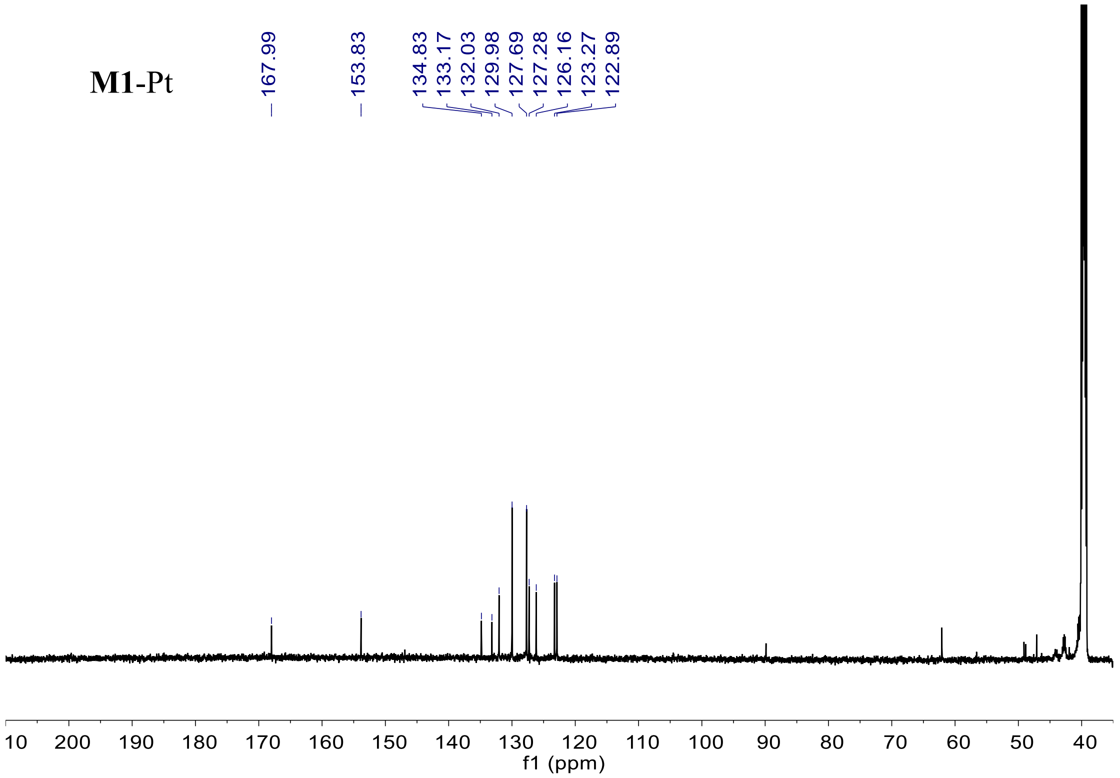

Figure S32. ^{13}C NMR spectrum of **M1**-Pt in DMSO-d_6.

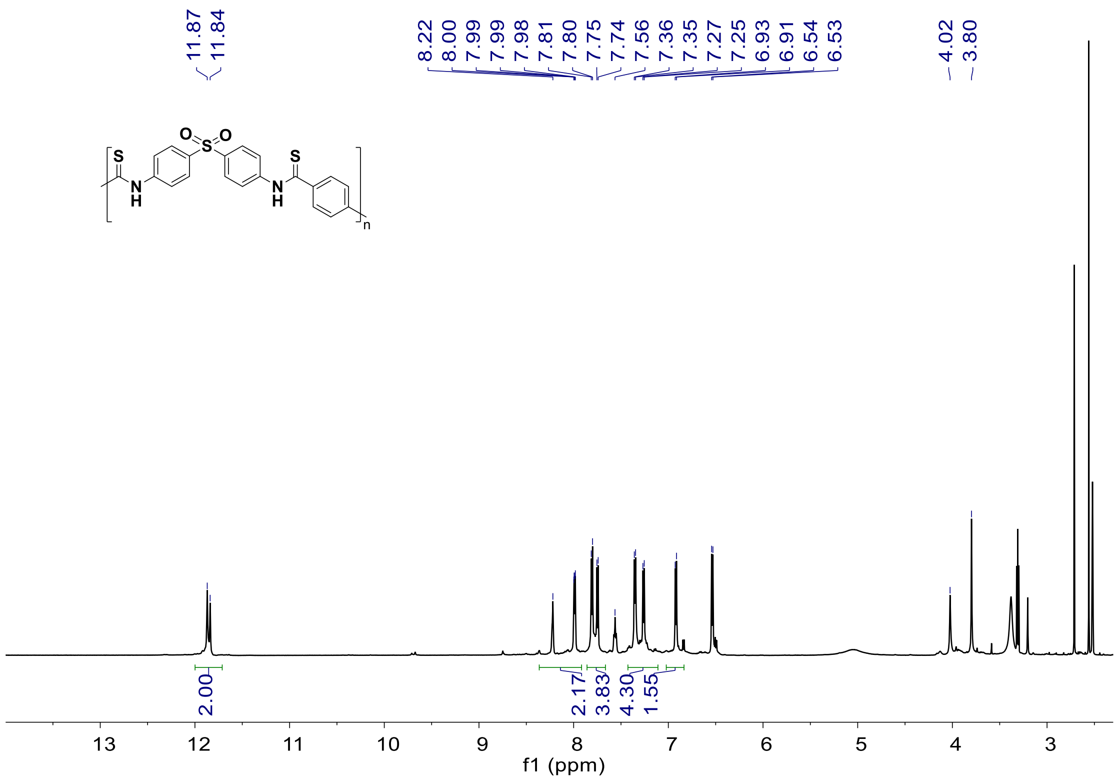

Figure S33. ^{1}H NMR spectrum of the synthesized polythioamide in DMSO-d_6.

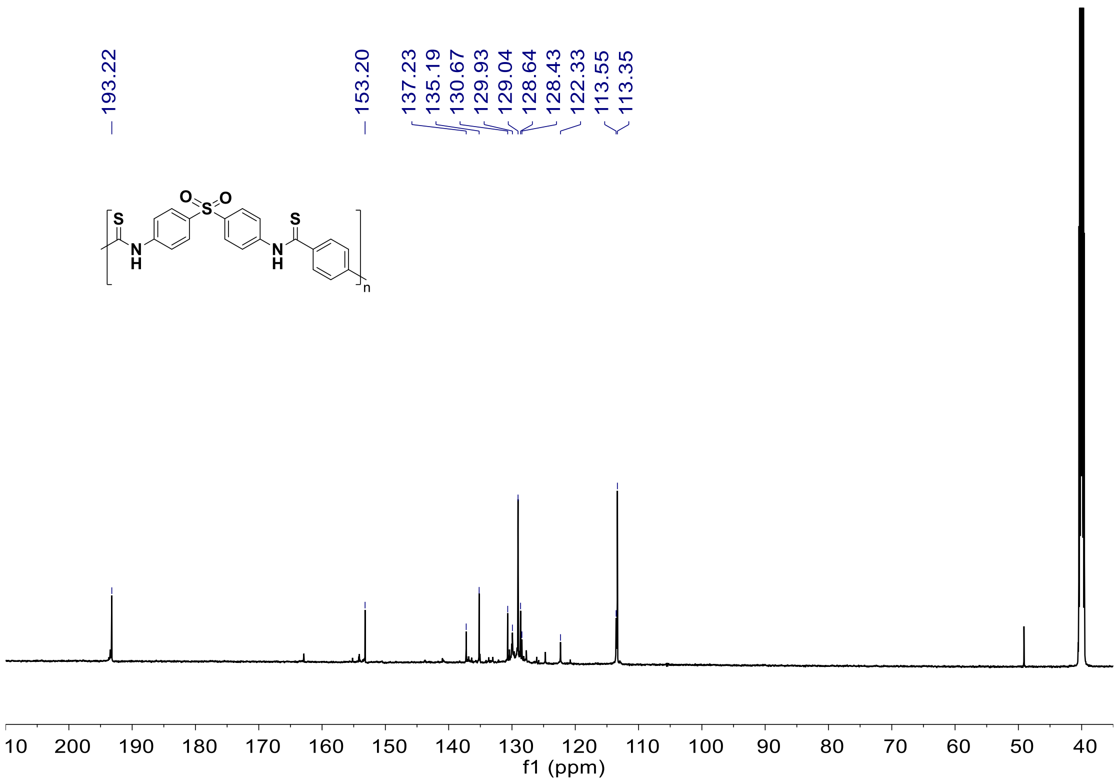

Figure S34. ^{13}C NMR spectrum of the synthesized polythioamide in DMSO-d_6.

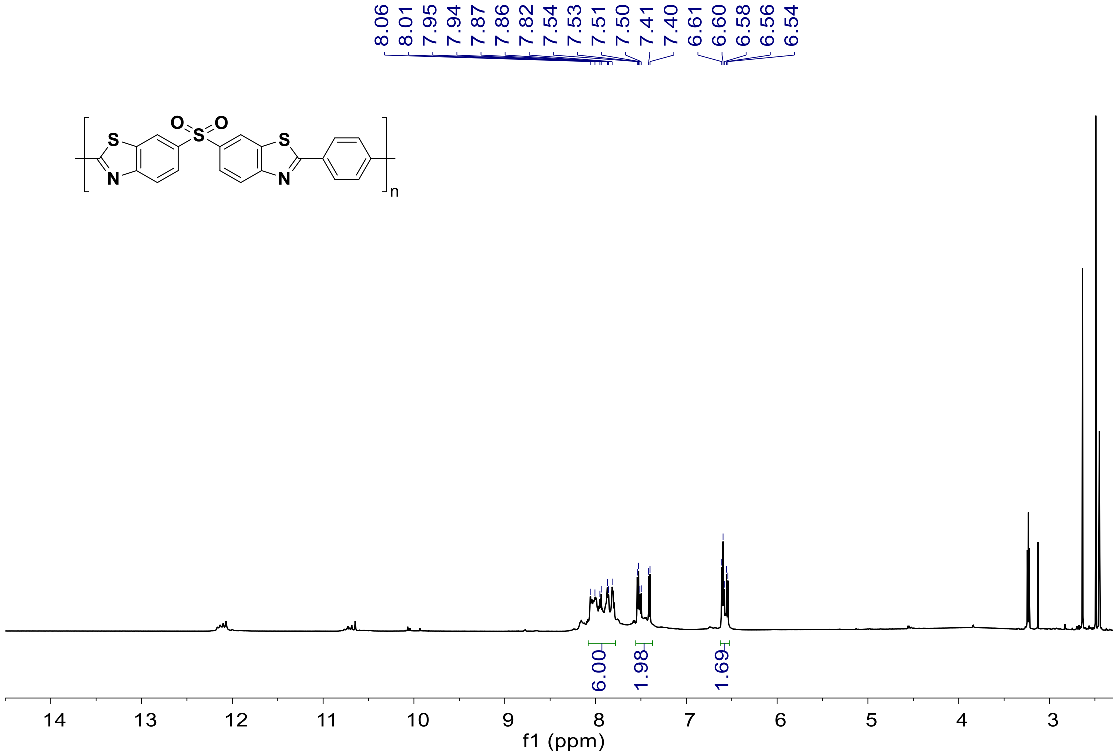

Figure S35. ^{1}H NMR spectrum of **P1** in DMSO-d_6.

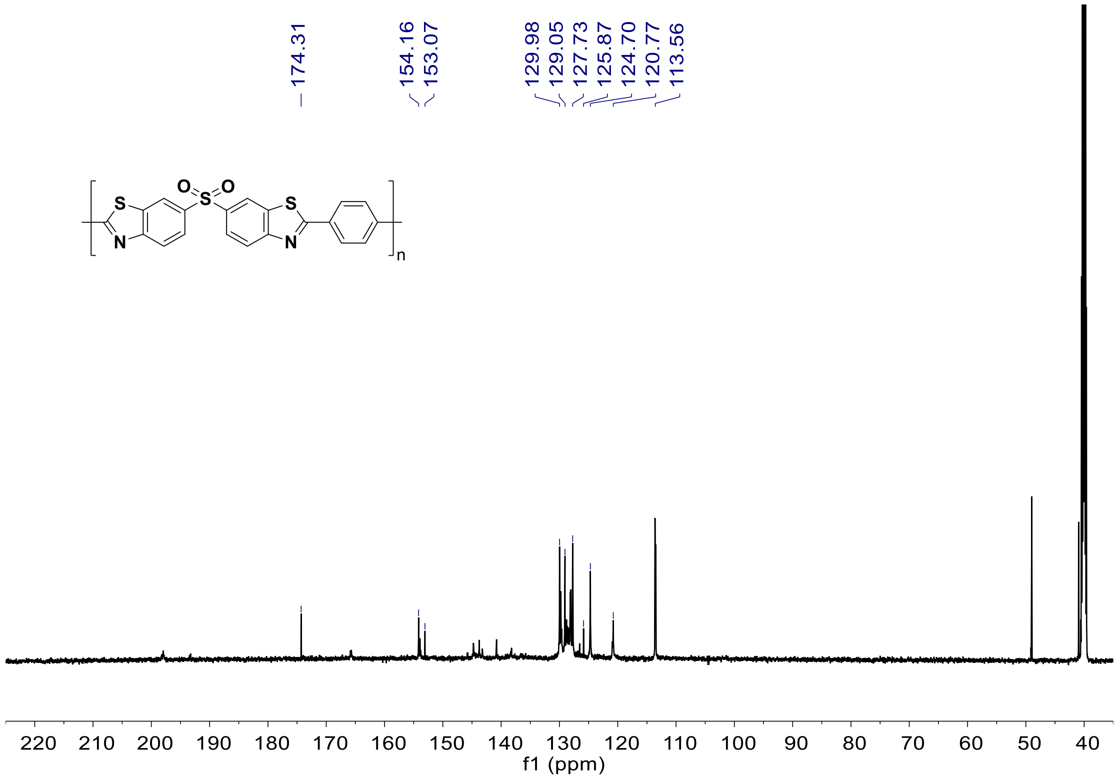

Figure S36. ^{13}C NMR spectrum of **P1** in DMSO-d_6.

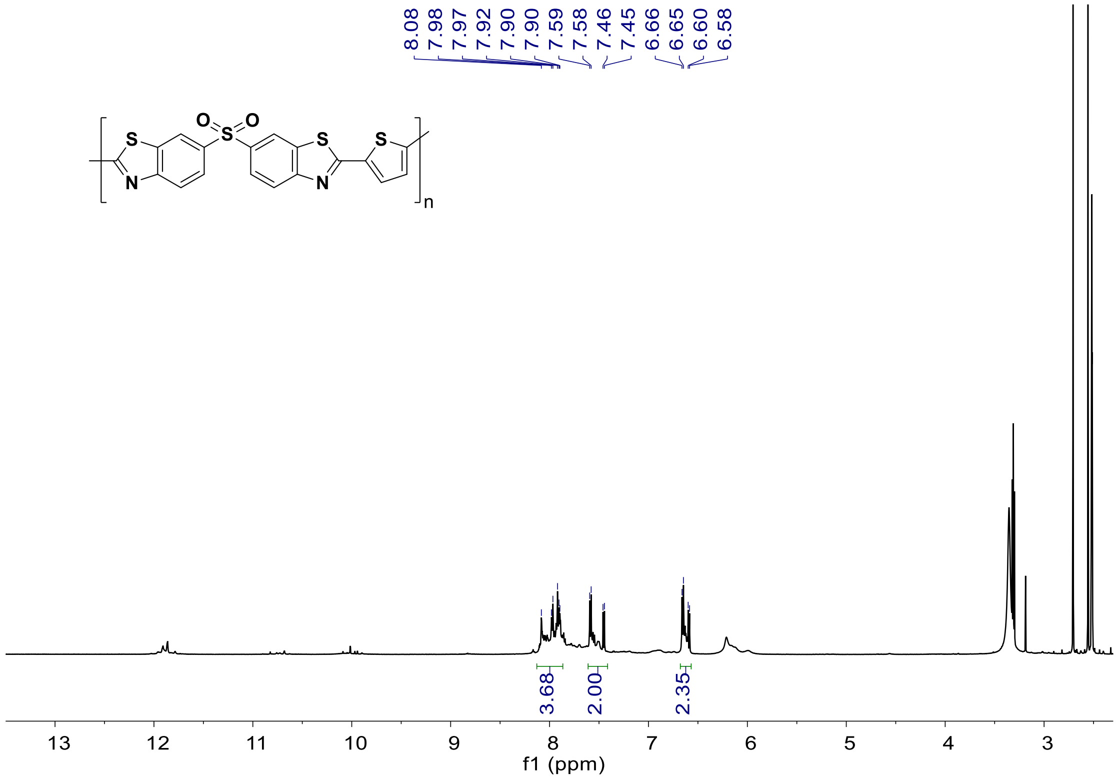

Figure S37. ^{1}H NMR spectrum of **P2** in DMSO-d_6.

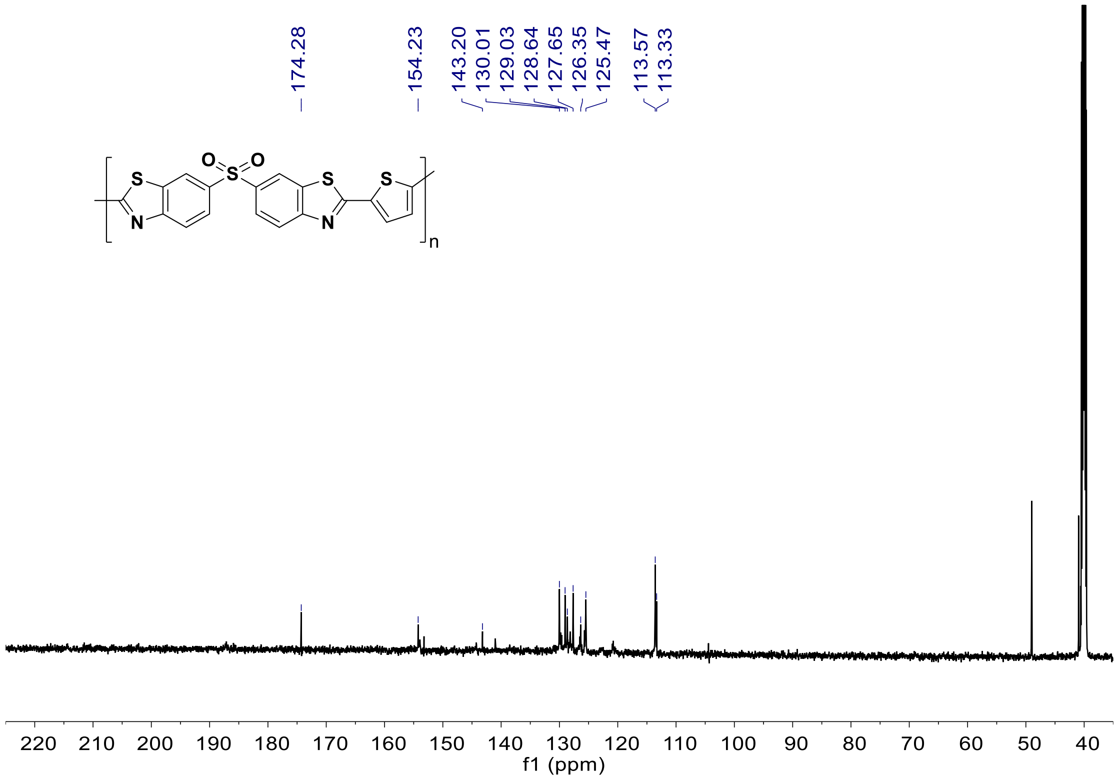

Figure S38. ^{13}C NMR spectrum of **P2** in DMSO-d_6.

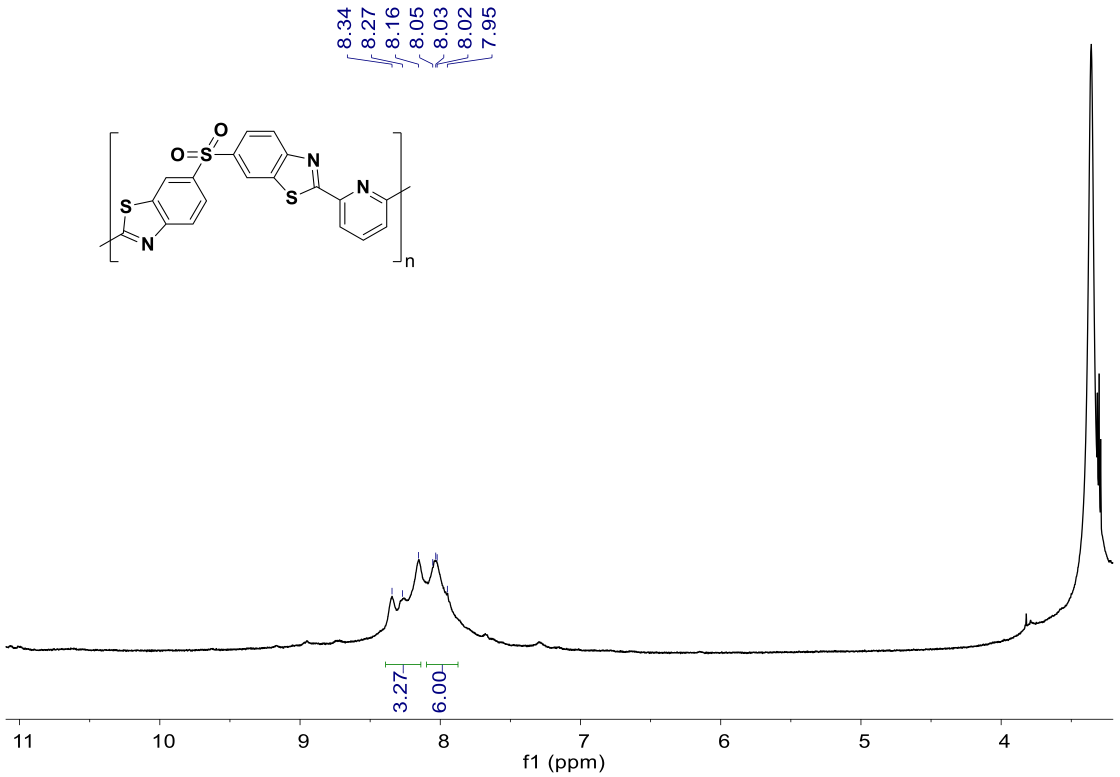

Figure S39. ^{1}H NMR spectrum of **P3** in DMSO-d_6.

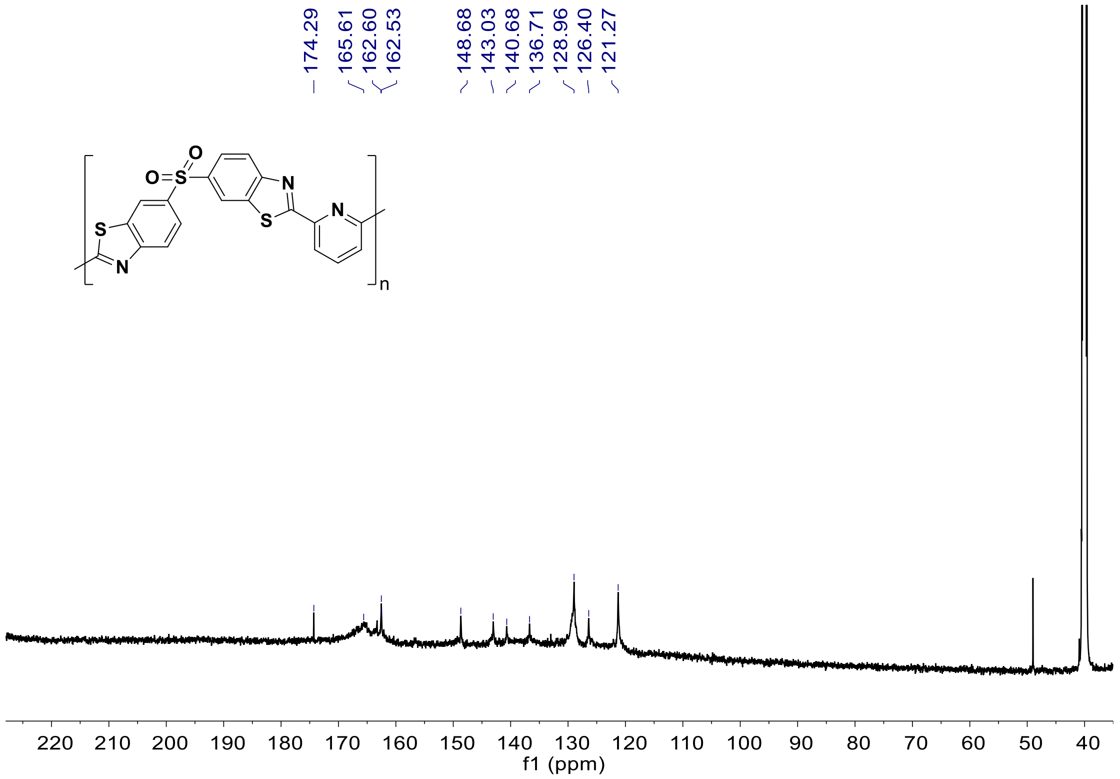

Figure S40. ^{13}C NMR spectrum of **P3** in DMSO-d_6.

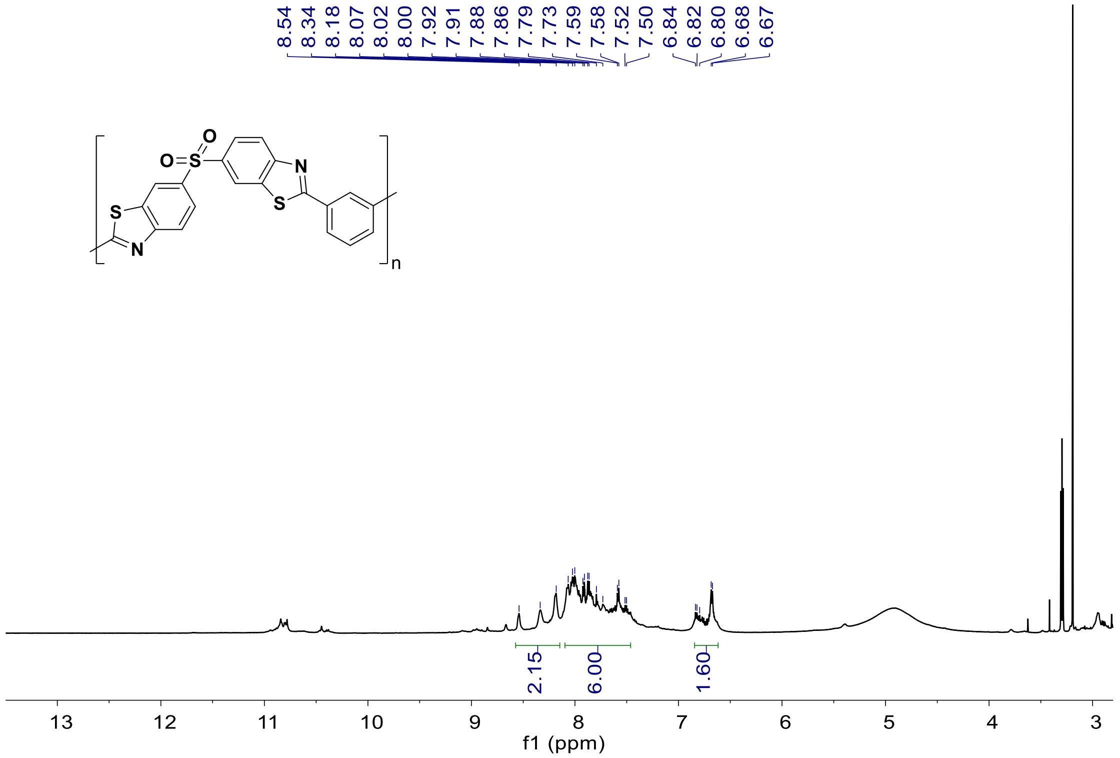

Figure S41. ^{1}H NMR spectrum of **P4** in DMSO-d_6.

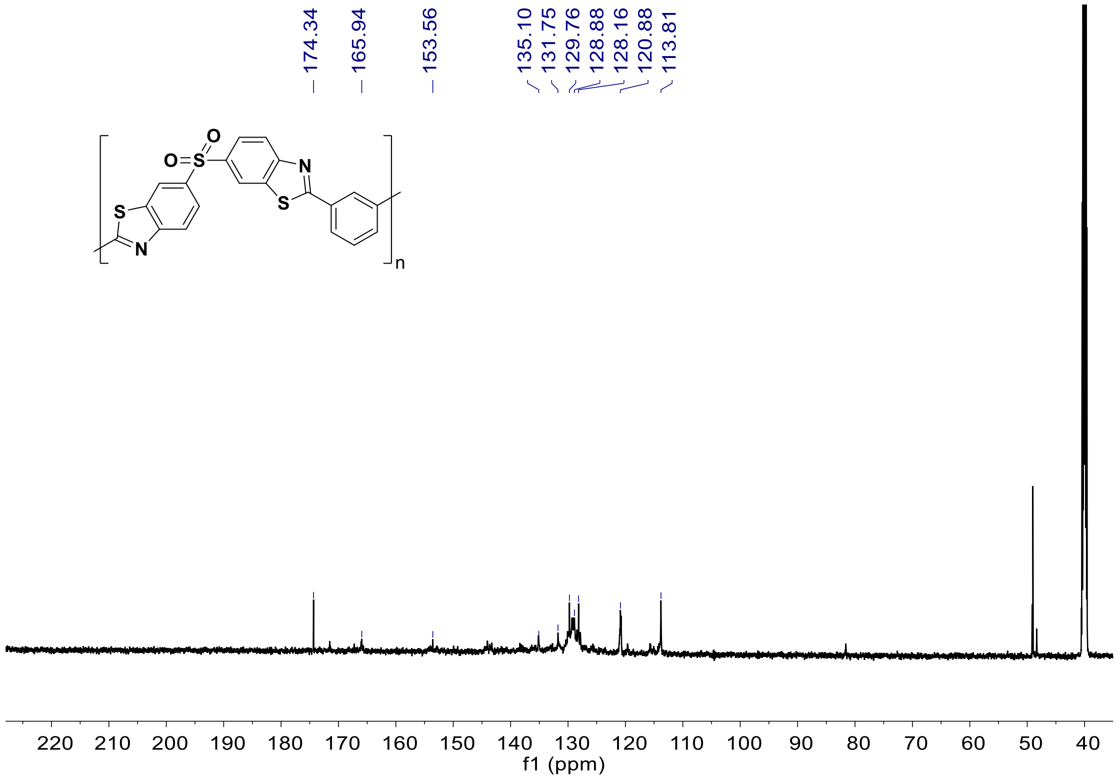

Figure S42. ^{13}C NMR spectrum of **P4** in DMSO-d_6.

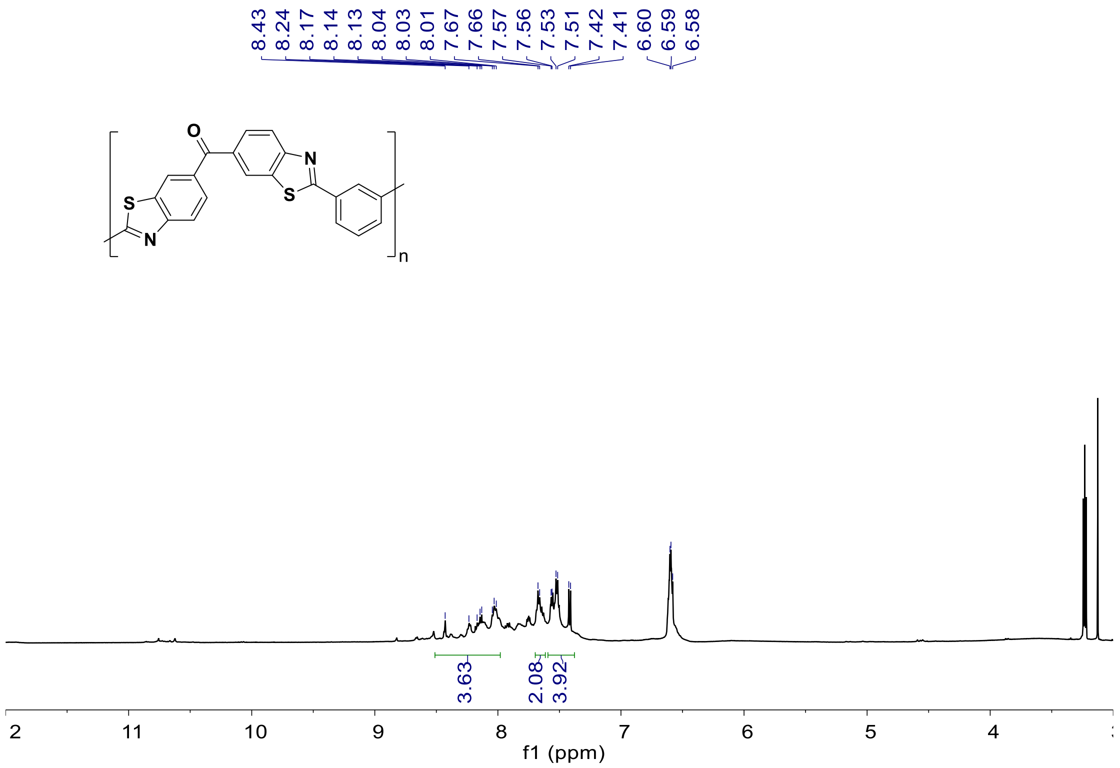

Figure S43. ^{1}H NMR spectrum of **P5** in DMSO-d_6.

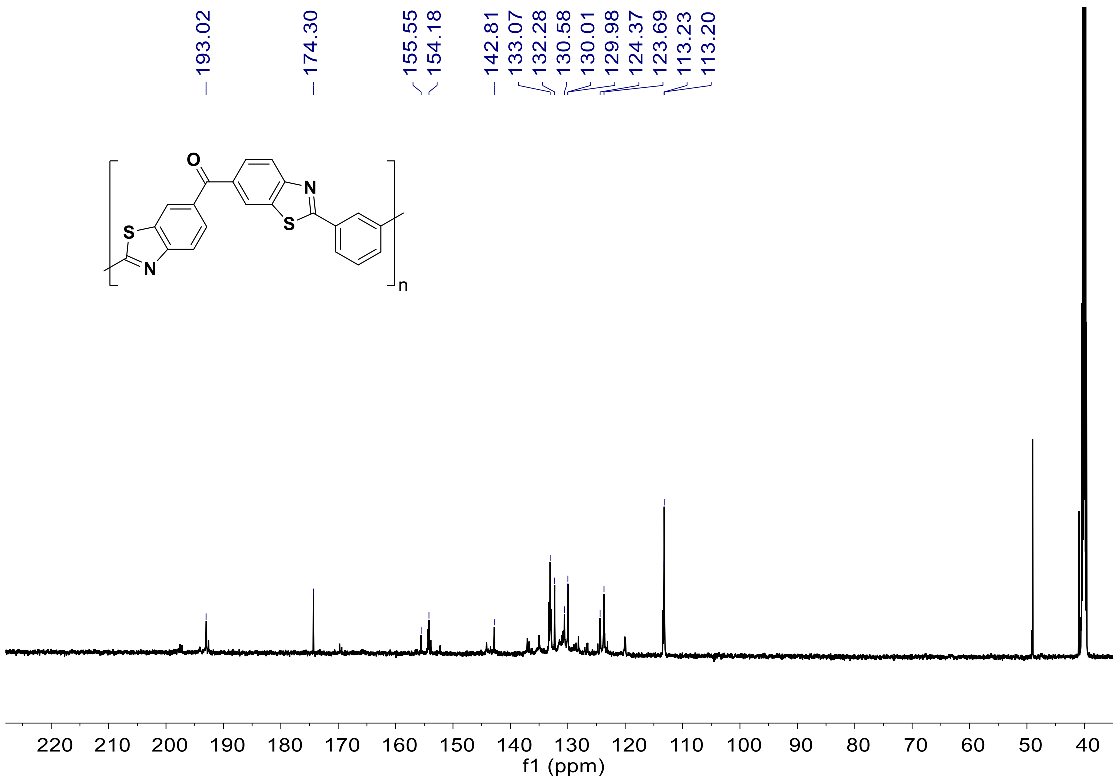

Figure S44. ^{13}C NMR spectrum of **P5** in DMSO-d_6.

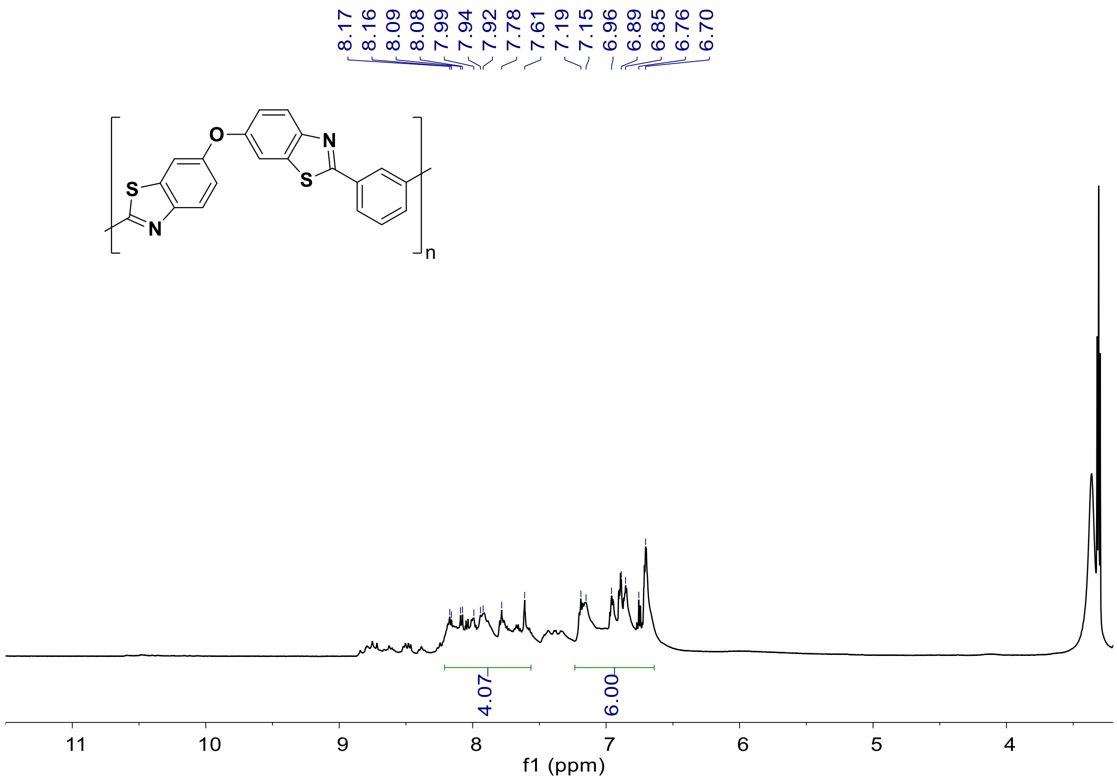

Figure S45. ^{1}H NMR spectrum of **P6** in DMSO-d_6.

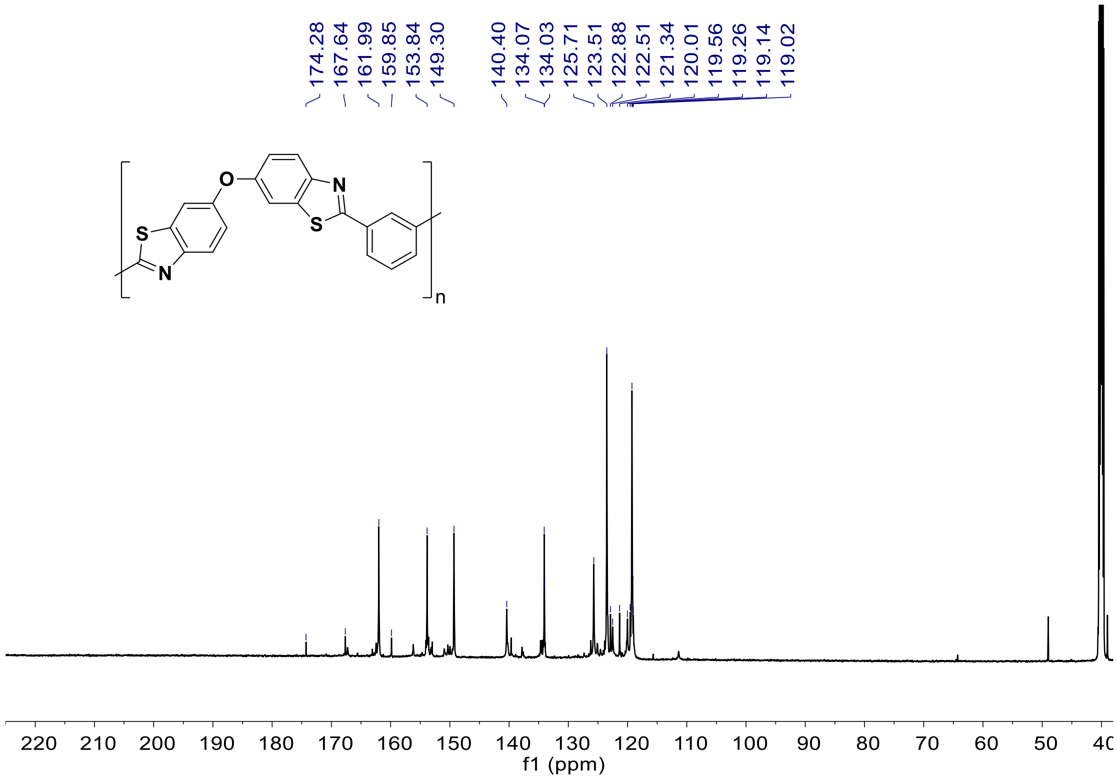

Figure S46. ^{13}C NMR spectrum of **P6** in DMSO-d_6.

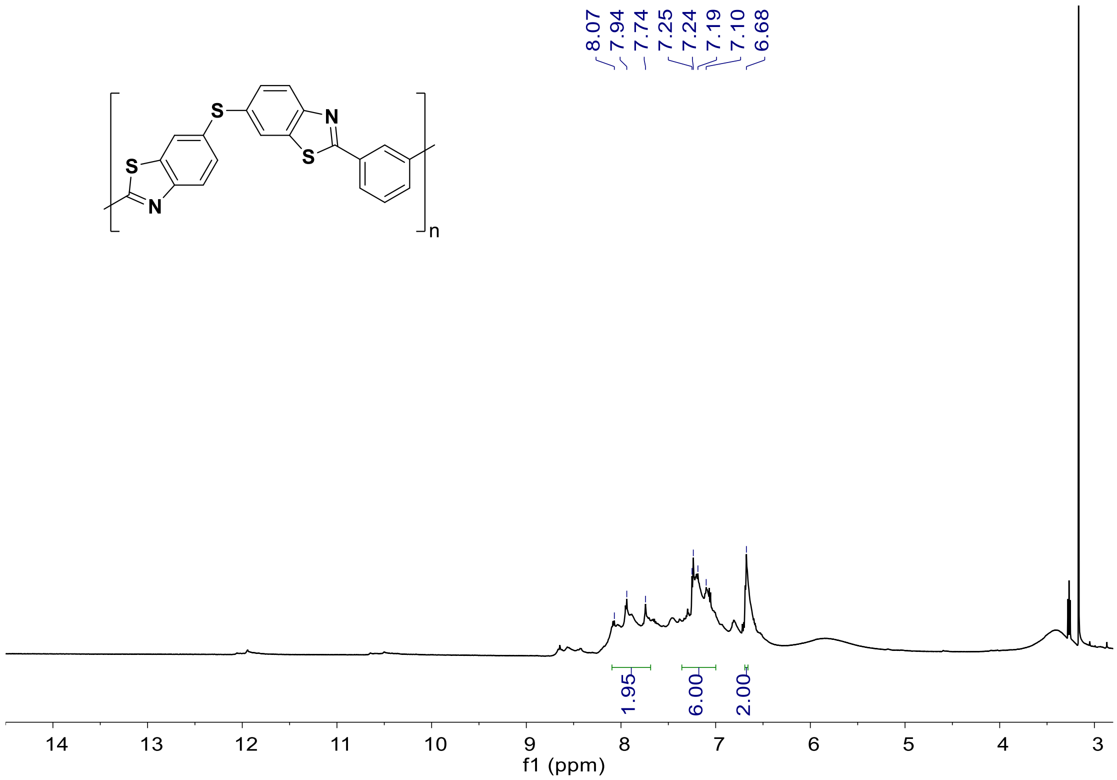

Figure S47. ^{1}H NMR spectrum of **P7** in DMSO-d_6.

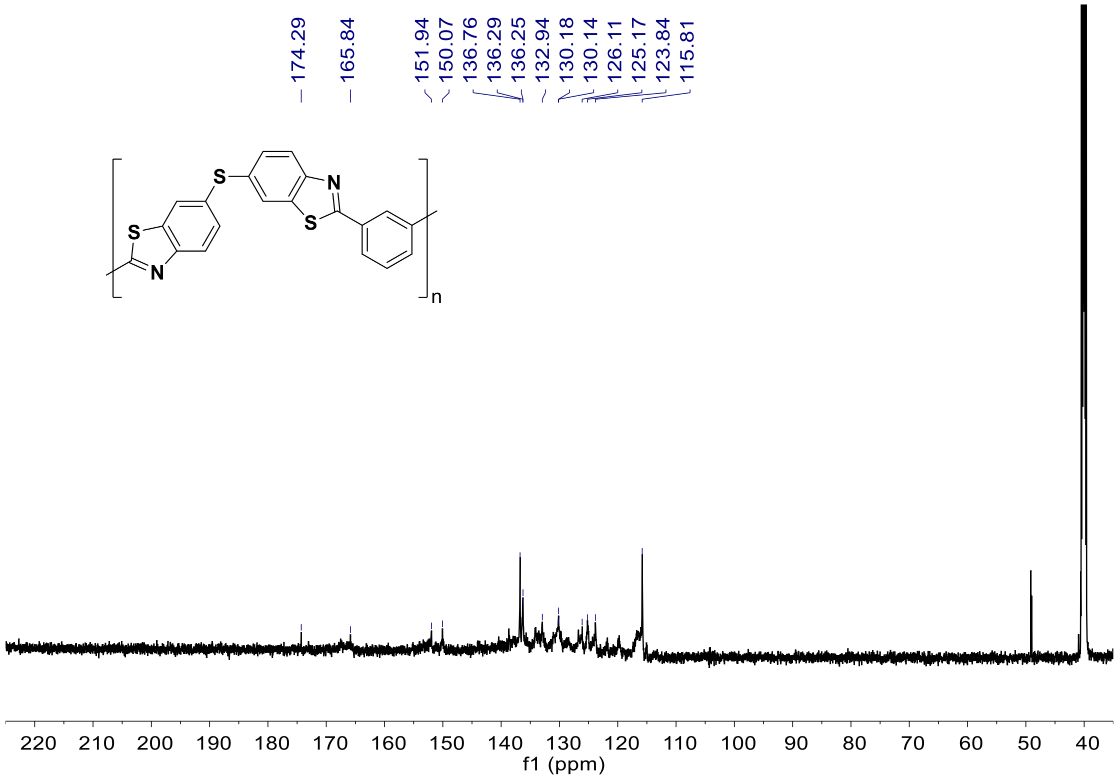

Figure S48. ^{13}C NMR spectrum of **P7** in DMSO-d_6.

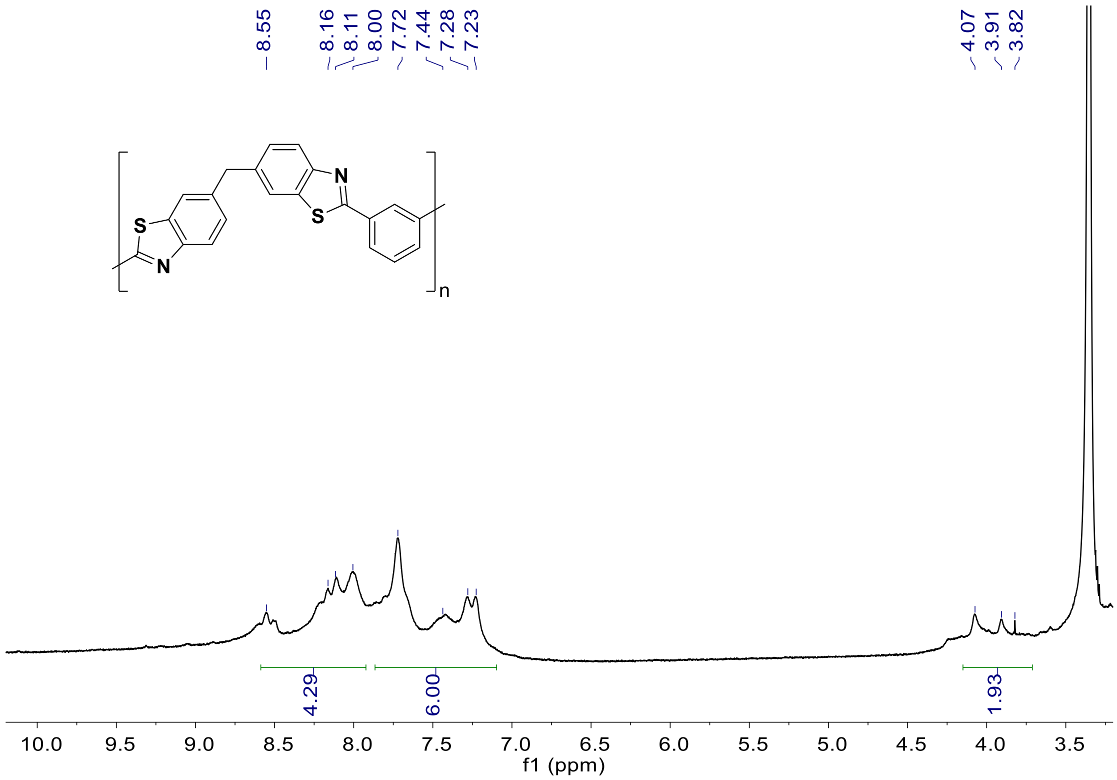

Figure S49. ^{1}H NMR spectrum of **P8** in DMSO-d_6.

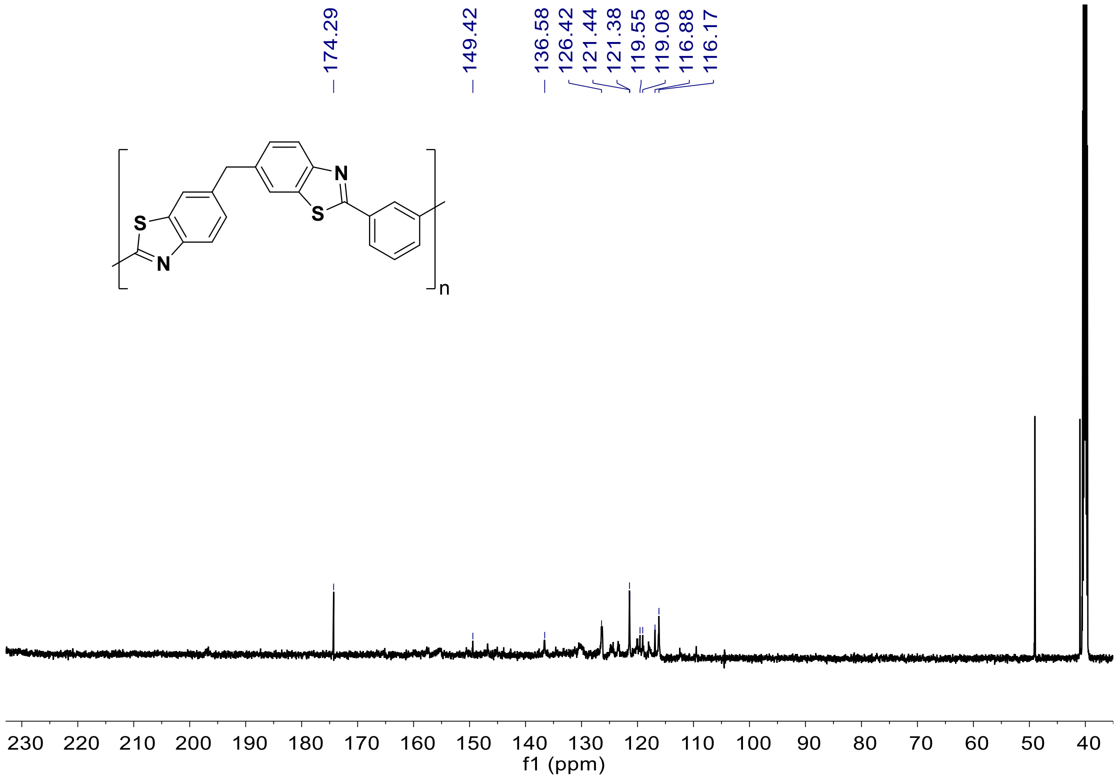

Figure S50. ^{13}C NMR spectrum of **P8** in DMSO-d_6.

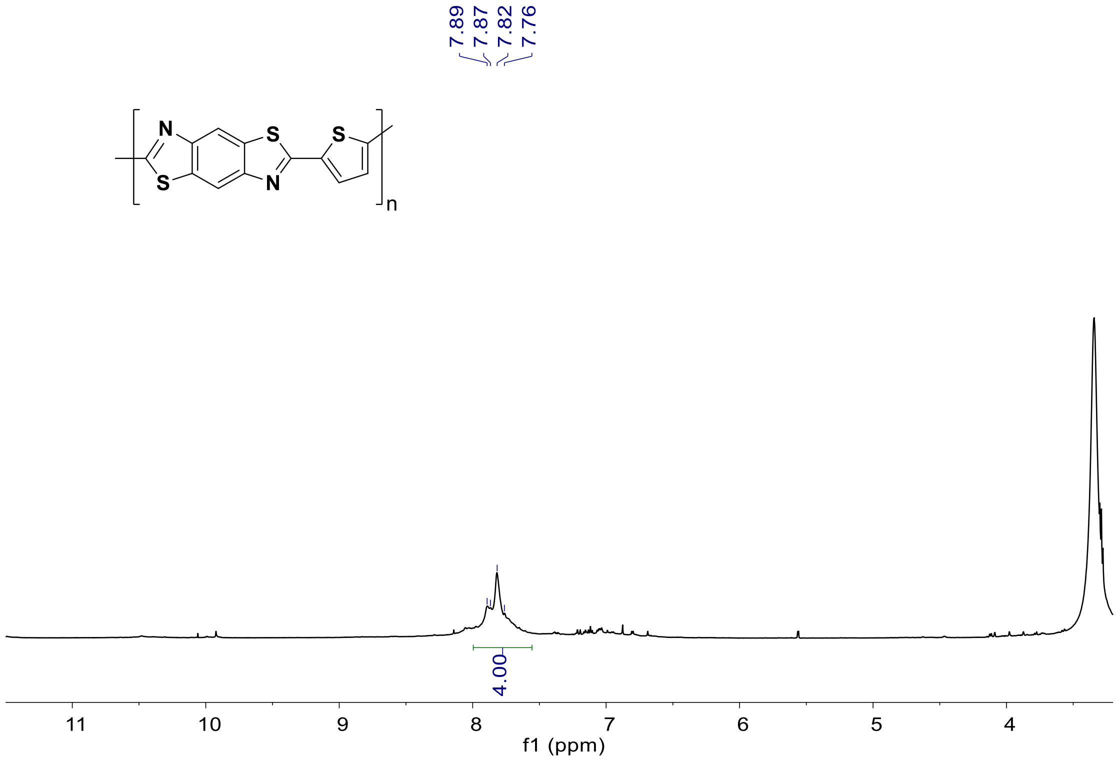

Figure S51. ^{1}H NMR spectrum of **P9** in DMSO-d_6.

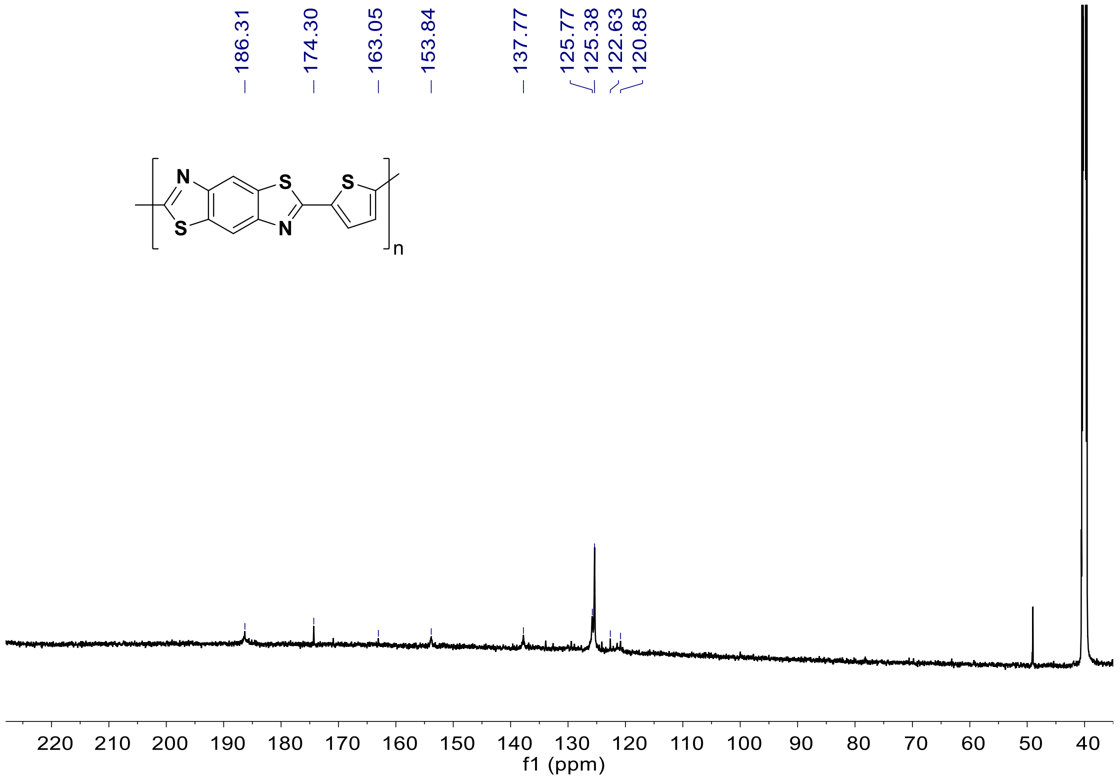

Figure S52. ^{13}C NMR spectrum of **P9** in DMSO-d_6.

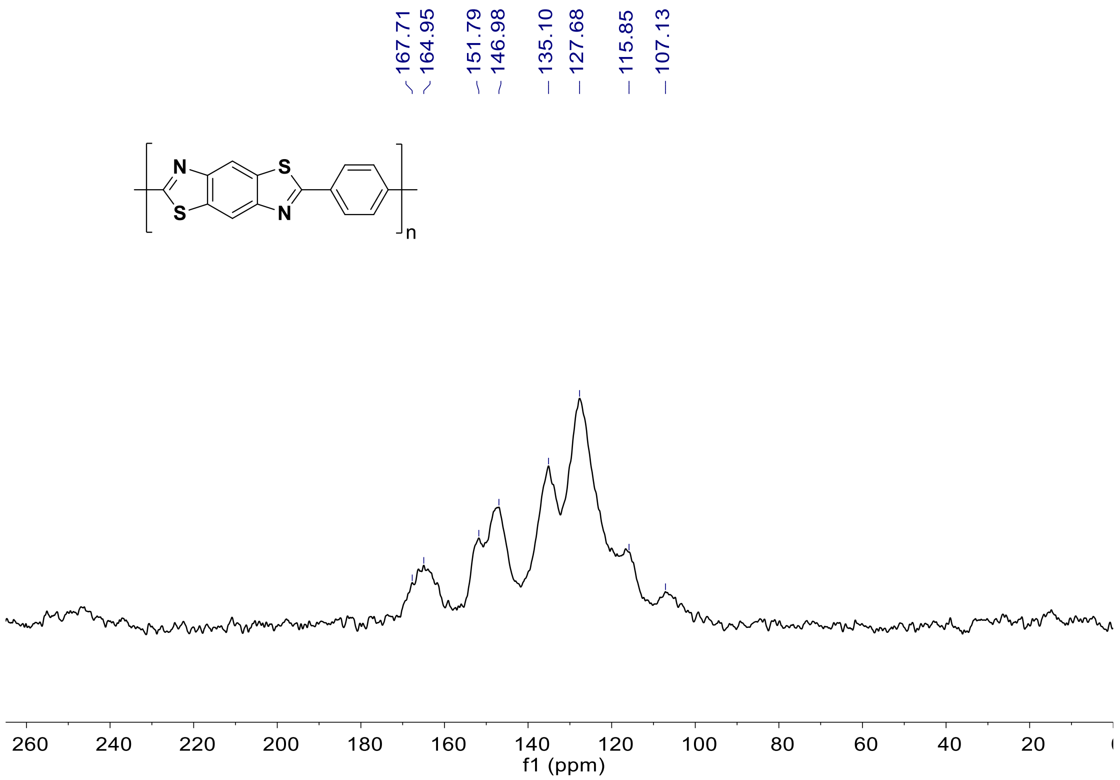

Figure S53. ^{13}C solid-state NMR spectrum of **P10**.

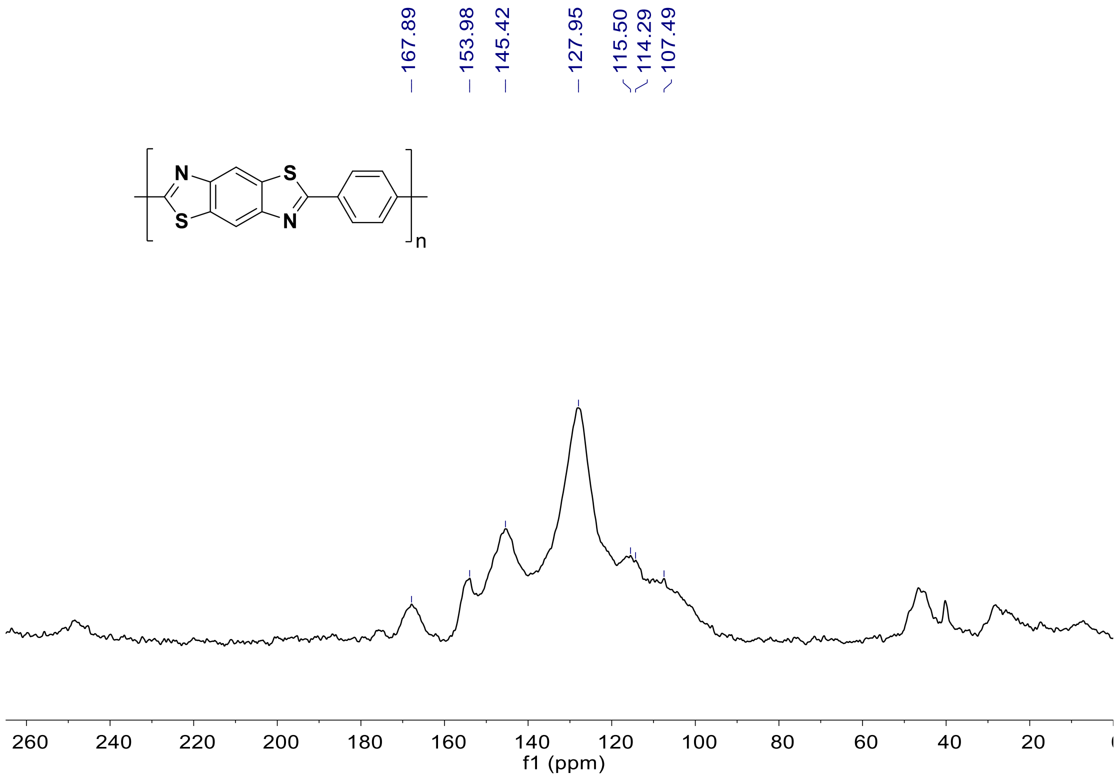

Figure S54. ^{13}C solid-state NMR spectrum of **P11**.

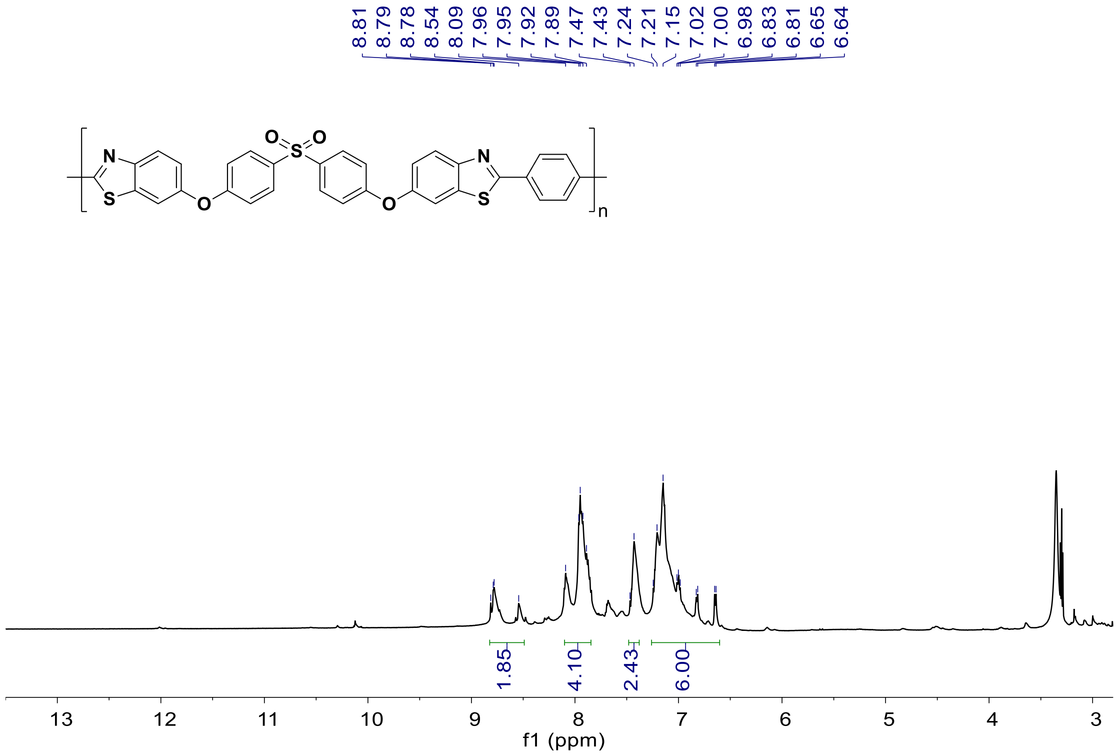

Figure S55. ^{1}H NMR spectrum of **P12** in DMSO-d_6.

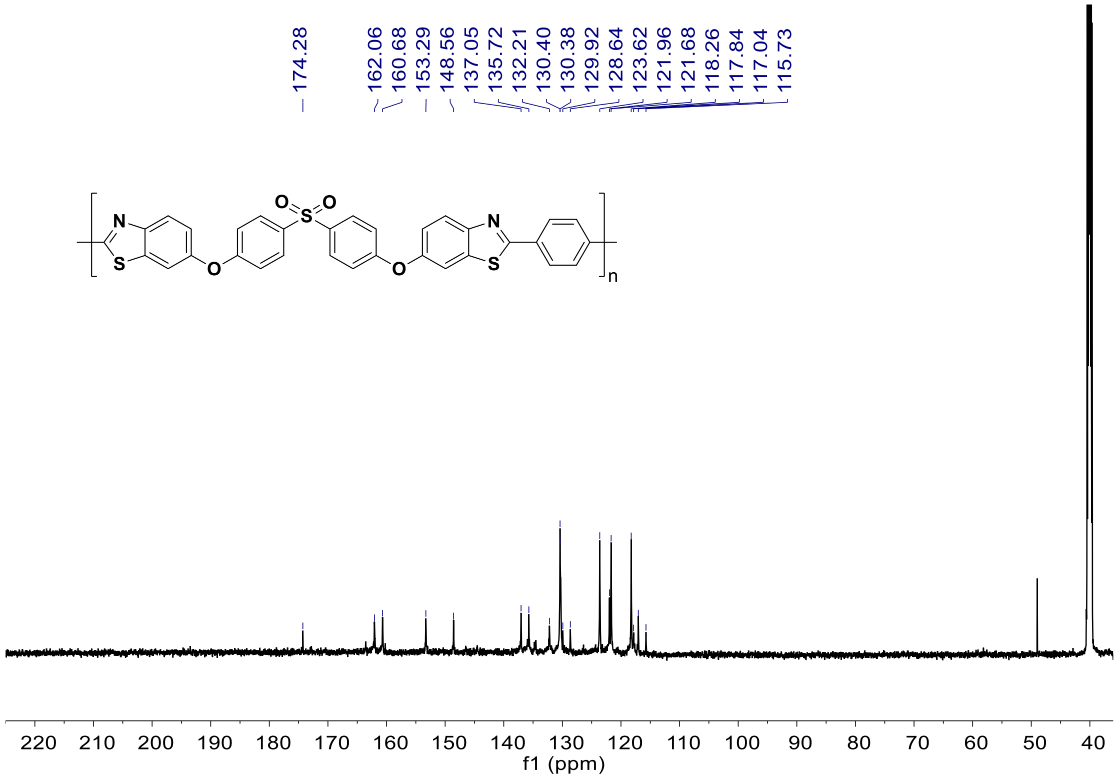

Figure S56. ^{13}C NMR spectrum of **P12** in DMSO-d_6.

References

[1] Zeng, C., Liu, P., Xiao, Z., Li, Y., Song, L., Cao, Z., Wu, D., et al., Highly Selective Adsorption and Recovery of Palladium from Spent Catalyst Wastewater by 1,4,7,10-Tetraazacyclododecane-Modified Mesoporous Silica, 2022, ACS Sustain. Chem. Eng., 10, 1103, 10.1021/acssuschemeng.1c05915

[2] Tang, J., Chen, Y., Wang, S., Kong, D., Zhang, L., Highly Efficient Metal-Organic Frameworks Adsorbent for Pd(II) and Au(III) Recovery from Solutions: Experiment and Mechanism, 2022, Environ. Res., 210, 112870, 10.1016/j.envres.2022.112870

[3] Zhang, D., MacDonald, L., Raj, P., Karamalidis, A. K., Thiol-Functionalized Cellulose Adsorbents for Highly Selective Separation of Palladium over Platinum in Acidic Aqueous Solutions, 2024, Chem. Eng. J., 494, 152948, 10.1016/j.cej.2024.152948

[4] Wu, H., Kim, S.-Y., Ito, T., Miwa, M., Matsuyama, S., One-Pot Synthesis of Silica-Gel-Based Adsorbent with Schiff Base Group for the Recovery of Palladium Ions from Simulated High-Level Liquid Waste, 2022, Nuc. Eng. Technol., 54, 3641, 10.1016/j.net.2022.04.024

[5] Sharma, S., Rajesh, N., Augmenting the Adsorption of Palladium from Spent Catalyst Using a Thiazole Ligand Tethered on an Amine Functionalized Polymeric Resin, 2016, Chem. Eng. J., 283, 999, 10.1016/j.cej.2015.08.061

[6] Kimuro, T., Gandhi, M. R., Kunda, U. M. R., Hamada, F., Yamada, M., Palladium(II) Sorption of a Diethylphosphate-Modified Thiacalix[6]Arene Immobilized on Amberlite Resin, 2017, Hydrometallurgy, 171, 254, 10.1016/j.hydromet.2017.05.022

[7] Yamada, M., Gandhi, M. R., Kondo, Y., Haga, K., Shibayama, A., Hamada, F., Selective Sorption of Palladium by Thiocarbamoyl-Substituted Thiacalix[N]Arene Derivatives Immobilized on Amberlite Resin: Application to Leach Liquors of Automotive Catalysts, 2015, RSC Adv., 5, 60506, 10.1039/C5RA07921G

[8] Jung, Y., Do, T., Su Choi, U., Jung, K.-W., Choi, J.-W., Cage-Like Amine-Rich Polymeric Capsule with Internal 3d Center-Radial Channels for Efficient and Selective Gold Recovery, 2022, Chem. Eng. J., 438, 135618, 10.1016/j.cej.2022.135618

[9] Chen, M., Li, S., Jin, C., Shao, M., Huang, Z., Xie, X., Removal of Metal-Cyanide Complexes and Recovery of Pt(II) and Pd(II) from Wastewater Using an Alkali–Tolerant Metal-Organic Resin, 2021, J. Hazard. Mater., 406, 124315, 10.1016/j.jhazmat.2020.124315

[10] Ianăşi, C., Svera , P., Popa, A., Lazău, R., Negrea, A., Negrea, P., Duteanu, N., et al., Adsorbent Material Based on Carbon Black and Bismuth with Tunable Properties for Gold Recovery, 2023, Materials, 16, 2837, 10.3390/ma16072837

[11] Wang, Z., Xu, X., Ma, S., Wang, H., Zhao, H., Wang, Y., Tong, S., et al., The Superior Adsorption Capacity of Boron-Nitrogen Co-Doping Walnut Shell Biochar Powder for Au(III), Pt(IV), and Pd(II), 2021, J. Environ. Chem. Eng., 9, 106288, 10.1016/j.jece.2021.106288

[12] Grad, O., Ciopec, M., Negrea, A., Duțeanu, N., Vlase, G., Negrea, P., Dumitrescu, C., et al., Precious Metals Recovery from Aqueous Solutions Using a New Adsorbent Material, 2021, Sci. Rep., 11, 2016, 10.1038/s41598-021-81680-z

[13] Zhou, L., Xu, J., Liang, X., Liu, Z., Adsorption of Platinum(IV) and Palladium(II) from Aqueous Solution by Magnetic Cross-Linking Chitosan Nanoparticles Modified with Ethylenediamine, 2010, J. Hazard. Mater., 182, 518, 10.1016/j.jhazmat.2010.06.062

[14] Liu, M., Long, X., Li, X., Du, Y., Zhao, Y., Huang, Z., Chen, Y., et al., Enrichment and Activation of Cyano in Cobalt Hexacyanoferrate for Specific Recovery of Ultra-Low Concentrations of Platinum, 2024, Sep. Purif. Technol., 328, 124925, 10.1016/j.seppur.2023.124925

[15] Asere, T. G., Mincke, S., Folens, K., Vanden Bussche, F., Lapeire, L., Verbeken, K., Van Der Voort, P., et al., Dialdehyde Carboxymethyl Cellulose Cross-Linked Chitosan for the Recovery of Palladium and Platinum from Aqueous Solution, 2019, React. Funct. Polym., 141, 145, 10.1016/j.reactfunctpolym.2019.05.008

[16] Parajuli, D., Khunathai, K., Adhikari, C. R., Inoue, K., Ohto, K., Kawakita, H., Funaoka, M., et al., Total Recovery of Gold, Palladium, and Platinum Using Lignophenol Derivative, 2009, Miner. Eng., 22, 1173, 10.1016/j.mineng.2009.06.003

[17] Adhikari, C. R., Parajuli, D., Kawakita, H., Inoue, K., Ohto, K., Harada, H., Dimethylamine-Modified Waste Paper for the Recovery of Precious Metals, 2008, Environ. Sci. Technol., 42, 5486, 10.1021/es800155x

[18] Uheida, A., Mònica, I., Clàudia, F., Yu, Z., and Muhammed, M., Adsorption Behavior of Platinum Group Metals (Pd, Pt, Rh) on Nonylthiourea-Coated Fe_3O_4 Nanoparticles, 2006, Sep. Sci. Technol., 41, 909,

10.1080/01496390600588952

[19] Ramesh, A., Hasegawa, H., Sugimoto, W., Maki, T., Ueda, K., Adsorption of Gold(III), Platinum(IV) and Palladium(II) onto Glycine Modified Crosslinked Chitosan Resin, 2008, Bioresour. Technol., 99, 3801, 10.1016/j.biortech.2007.07.008

[20] Liu, L., Liu, S., Zhang, Q., Li, C., Bao, C., Liu, X., Xiao, P., Adsorption of Au(III), Pd(II), and Pt(IV) from Aqueous Solution onto Graphene Oxide, 2013, J. Chem. Eng. Data, 58, 209, 10.1021/je300551c

[21] Sharma, S., Rajesh, N., Synergistic Influence of Graphene Oxide and Tetraoctylammonium Bromide (Frozen Ionic Liquid) for the Enhanced Adsorption and Recovery of Palladium from an Industrial Catalyst, 2016, J. Environ. Chem. Eng., 4, 4287, 10.1016/j.jece.2016.09.028

[22] Saha, S., Venkatesh, M., Basu, H., Pimple, M. V., Singhal, R. K., Recovery of Gold Using Graphene Oxide/Calcium Alginate Hydrogel Beads from a Scrap Solid State Detector, 2019, J. Environ. Chem. Eng., 7, 103134, 10.1016/j.jece.2019.103134

[23] Shin, S. S., Jung, Y., Jeon, S., Park, S.-J., Yoon, S.-J., Jung, K.-W., Choi, J.-W., et al., Efficient Recovery and Recycling/Upcycling of Precious Metals Using Hydrazide-Functionalized Star-Shaped Polymers, 2024, Nat. Comm., 15, 3889, 10.1038/s41467-024-48090-x

[24] Ramakul, P., Yanachawakul, Y., Leepipatpiboon, N., Sunsandee, N., Biosorption of Palladium(II) and Platinum(IV) from Aqueous Solution Using Tannin from Indian Almond (Terminalia Catappa L.) Leaf Biomass: Kinetic and Equilibrium Studies, 2012, Chem. Eng. J., 193-194, 102, 10.1016/j.cej.2012.04.035

[25] Bai, F., Ye, G., Chen, G., Wei, J., Wang, J., Chen, J., Highly Selective Recovery of Palladium by a New Silica-Based Adsorbent Functionalized with Macrocyclic Ligand, 2013, Sep. Purif. Technol., 106, 38, 10.1016/j.seppur.2012.12.021

[26] Rasoulzadeh, H., Sheikhmohammadi, A., Abtahi, M., Roshan, B., Jokar, R., Eco-Friendly Rapid Removal of Palladium from Aqueous Solutions Using Alginate-Diatomite Magnano Composite, 2021, J. Environ. Chem. Eng., 9, 105954, 10.1016/j.jece.2021.105954

[27] Yen, C.-H., Lien, H.-L., Chung, J.-S., Yeh, H.-D., Adsorption of Precious Metals in Water by Dendrimer Modified Magnetic Nanoparticles, 2017, J. Hazard. Mater., 322, 215, 10.1016/j.jhazmat.2016.02.029

[28] Yamada, M., Kimura, S., Rajiv Gandhi, M., Shibayama, A., Environmentally Friendly Pd(II) Recovery from Spent Automotive Catalysts Using Resins Impregnated with a Pincer-Type Extractant, 2021, Sci. Rep., 11, 365, 10.1038/s41598-020-79614-2

[29] Zalupski, P. R., McDowell, R., Dutech, G., The Adsorption of Gold, Palladium, and Platinum from Acidic Chloride Solutions on Mesoporous Carbons, 2014, Solvent Extr. Ion Exch., 32, 737, 10.1080/07366299.2014.951278

[30] Lim, C.-R., Lin, S., Yun, Y.-S., Highly Efficient and Acid-Resistant Metal-Organic Frameworks of MIL-101(Cr)-NH_2 for Pd(II) and Pt(IV) Recovery from Acidic Solutions: Adsorption Experiments, Spectroscopic Analyses, and Theoretical Computations, 2020, J. Hazard. Mater., 387, 121689, 10.1016/j.jhazmat.2019.121689

[31] Kraus, A., Jainae, K., Unob, F., Sukpirom, N., Synthesis of MPTS-Modified Cobalt Ferrite Nanoparticles and Their Adsorption Properties in Relation to Au(III), 2009, J. Colloid. Interface Sci., 338, 359, 10.1016/j.jcis.2009.06.045

[32] Morcali, M. H., Zeytuncu, B., Investigation of Adsorption Parameters for Platinum and Palladium onto a Modified Polyacrylonitrile-Based Sorbent, 2015, Int. J. Miner. Process., 137, 52, 10.1016/j.minpro.2015.02.011

[33] Qing, Y., Hang, Y., Wanjaul, R., Jiang, Z., Hu, B., Adsorption Behavior of Noble Metal Ions (Au, Ag, Pd) on Nanometer-Size Titanium Dioxide with ICP-AES, 2003, Anal. Sci., 19, 1417, 10.2116/analsci.19.1417

[34] Maponya, T. C., Modibane, K. D., Somo, T. R., Makgopa, K., Selective Adsorption of Palladium Ions from Wastewater by Ion-Imprinted MIL-101(Cr) Derived from Waste Polyethylene Terephthalate: Isotherms and Kinetics, 2023, Sep. Purif. Technol., 307, 122767, 10.1016/j.seppur.2022.122767

[35] Wang, S., Vincent, T., Roux, J.-C., Faur, C., Guibal, E., Pd(II) and Pt(IV) Sorption Using Alginate and Algal-Based Beads, 2017, Chem. Eng. J., 313, 567, 10.1016/j.cej.2016.12.039

[36] Fan, R., Min, H., Hong, X., Yi, Q., Liu, W., Zhang, Q., Luo, Z., Plant Tannin Immobilized Fe_3O_4@SiO_2 Microspheres: A Novel and Green Magnetic Bio-Sorbent with Superior Adsorption Capacities for Gold and Palladium, 2019, J. Hazard. Mater., 364, 780, 10.1016/j.jhazmat.2018.05.061

[37] Zhang, L., Zha, X., Zhang, G., Gu, J., Zhang, W., Huang, Y., Zhang, J., et al., Designing a Reductive Hybrid Membrane to Selectively Capture Noble Metallic Ions During Oil/Water Emulsion Separation with Further Function Enhancement, 2018, J. Mater. Chem. A, 6, 10217, 10.1039/C8TA01864B

[38] Li, B., Xiong, W., Cao, Y., Zhou, X., Zhu, H., Li, M., Yang, L., et al., Targeting of Platinum Capture under 1+1 Aqua Regia Using Robust and Recyclable Polymeric Polyamine Resin: Adsorption Performance and Mechanism, 2023, Environ. Res., 227, 115814, 10.1016/j.envres.2023.115814

[39] Fayemi, O. E., Ogunlaja, A. S., Kempgens, P. F. M., Antunes, E., Torto, N., Nyokong, T., Tshentu, Z. R., Adsorption and Separation of Platinum and Palladium by Polyamine Functionalized Polystyrene-Based Beads and Nanofibers, 2013, Miner. Eng., 53, 256, 10.1016/j.mineng.2013.06.006

[40] Garland, N., Gordon, R., Hopkins, I., Ward, E., McElroy, C. R., MacQuarrie, D., Parkin, A., Polysaccharide-Derived Sulfur-Containing Mesoporous Carbon Materials for Platinum Group Metal Recovery, 2025, Carbon, 239, 120309, 10.1016/j.carbon.2025.120309

[41] Ning, S., Zhang, S., Zhang, W., Zhou, J., Wang, S., Wang, X., Wei, Y., Separation and Recovery of Rh, Ru and Pd from Nitrate Solution with a Silica-Based isoBu-BTP/SiO_2-P Adsorbent, 2020, Hydrometallurgy, 191, 105207, 10.1016/j.hydromet.2019.105207

[42] Liu, H., Ning, S., Li, Z., Zhang, S., Chen, L., Yin, X., Fujita, T., et al., Preparation of a Novel Silica-Based N-Donor Ligand Functional Adsorbent for Efficient Separation of Palladium from High Level Liquid Waste, 2022, Sep. Purif. Technol., 296, 121373, 10.1016/j.seppur.2022.121373

[43] Xu, L., Zhang, A., Pu, N., Xu, C., Chen, J., Development of Two Novel Silica Based Symmetric Triazine-Ring Opening N-Donor Ligands Functional Adsorbents for Highly Efficient Separation of Palladium from HNO_3 Solution, 2019, J. Hazard. Mater., 376, 188, 10.1016/j.jhazmat.2019.05.028

[44] Torrejos, R. E. C., Escobar, E. C., Han, J. W., Min, S. H., Yook, H., Parohinog, K. J., Koo, S., et al., Multidentate Thia-Crown Ethers as Hyper-Crosslinked Macroporous Adsorbent Resins for the Efficient Pd/Pt Recovery and Separation from Highly Acidic Spent Automotive Catalyst Leachate, 2021, Chem. Eng. J., 424, 130379, 10.1016/j.cej.2021.130379

[45] Kim, J., Kim, K. R., Hong, Y., Choi, S., Yavuz, C. T., Kim, J. W., Nam, Y. S., Photochemically Enhanced Selective Adsorption of Gold Ions on Tannin-Coated Porous Polymer Microspheres, 2019, ACS Appl. Mater. Interfaces, 11, 21915, 10.1021/acsami.9b05197

[46] Gurung, M., Adhikari, B. B., Alam, S., Kawakita, H., Ohto, K., Inoue, K., Persimmon Tannin-Based New Sorption Material for Resource Recycling and Recovery of Precious Metals, 2013, Chem. Eng. J., 228, 405, 10.1016/j.cej.2013.05.011

[47] Kang, T., Park, Y., Yi, J., Highly Selective Adsorption of Pt^{2+} and Pd^{2+} Using Thiol-Functionalized Mesoporous Silica, 2004, Ind. Eng. Chem. Res., 43, 1478, 10.1021/ie030590k

[48] Zhou, L., Liu, J., Liu, Z., Adsorption of Platinum(IV) and Palladium(II) from Aqueous Solution by Thiourea-Modified Chitosan Microspheres, 2009, J. Hazard. Mater., 172, 439, 10.1016/j.jhazmat.2009.07.030

[49] Wu, C., Zhu, X., Wang, Z., Yang, J., Li, Y., Gu, J., Specific Recovery and in Situ Reduction of Precious Metals from Waste to Create MOF Composites with Immobilized Nanoclusters, 2017, Ind. Eng. Chem. Res., 56, 13975, 10.1021/acs.iecr.7b02839

[50] Lin, S., Kumar Reddy, D. H., Bediako, J. K., Song, M.-H., Wei, W., Kim, J.-A., Yun, Y.-S., Effective Adsorption of Pd(II), Pt(IV) and Au(III) by Zr(IV)-Based Metal–Organic Frameworks from Strongly Acidic Solutions, 2017, J. Mater. Chem. A, 5, 13557, 10.1039/C7TA02518A

[51] Zha, M., Liu, J., Wong, Y.-L., Xu, Z., Extraction of Palladium from Nuclear Waste-Like Acidic Solutions by a Metal–Organic Framework with Sulfur and Alkene Functions, 2015, J. Mater. Chem. A, 3, 3928, 10.1039/C4TA06678B

[52] Lin, S., Bediako, J. K., Cho, C.-W., Song, M.-H., Zhao, Y., Kim, J.-A., Choi, J.-W., et al., Selective Adsorption of Pd(II) over Interfering Metal Ions (Co(II), Ni(II), Pt(IV)) from Acidic Aqueous Phase by Metal-Organic Frameworks, 2018, Chem. Eng. J., 345, 337, 10.1016/j.cej.2018.03.173

[53] Frisch, M. J., Trucks, G. W., Schlegel, H. B., Scuseria, G. E., Robb, M. A., Cheeseman, J. R., Scalmani, G., et al., Wallingford, CT, **2016**

[54] Vetere, V., Adamo, C., Maldivi, P., Performance of the 'Parameter Free' PBE0 Functional for the Modeling of Molecular Properties of Heavy Metals, 2000, Chem. Phys. Lett., 325, 99, 10.1016/S0009-2614(00)00657-6

[55] Grimme, S., Ehrlich, S., Goerigk, L., Effect of the Damping Function in Dispersion Corrected Density Functional Theory, 2011, J. Comput. Chem., 32, 1456, 10.1002/jcc.21759

[56] Weigend, F., Ahlrichs, R., Balanced Basis Sets of Split Valence, Triple Zeta Valence and Quadruple Zeta Valence Quality for H to Rn: Design and Assessment of Accuracy, 2005, Phys. Chem. Chem. Phys., 7, 3297, 10.1039/B508541A

[57] Miertuš, S., Scrocco, E., Tomasi, J., Electrostatic Interaction of a Solute with a Continuum. A Direct Utilizaion of Ab Initio Molecular Potentials for the Prevision of Solvent Effects, 1981, Chem. Phys., 55, 117, 10.1016/0301-0104(81)85090-2

[58] Schäfer, A., Huber, C., Ahlrichs, R., Fully Optimized Contracted Gaussian Basis Sets of Triple Zeta Valence Quality for Atoms Li to Kr, 1994, J. Chem. Phys., 100, 5829, 10.1063/1.467146
